# Supplemental paste composition data from the Manialtepec Basin, Oaxaca

**DOI:** 10.1016/j.dib.2019.103805

**Published:** 2019-03-08

**Authors:** Sarah B. Barber, Daniel E. Pierce

**Affiliations:** a University of Central Florida, USA; b University of Missouri Research Reactor, USA

## Abstract

This data article contains archaeological context information and paste compositional data from 66 pottery sherds collected at seven archaeological sites in the Manialtepec Basin on the Pacific coast of Oaxaca, Mexico. The data include maps showing collection locations, a drawing of one archaeological profile, photographs of sherds, and compositional data produced by Instrumental Neutron Activation Analysis at the University of Missouri Research Reactor (MURR). The NAA data include a tabulation of principal components, data from log-based cluster analyses and compositional group defining discriminant analyses. The data also include bootstrapped Mahalanobis distance calculations. For data interpretation, refer to “Ceramic Production and Consumption in an In-Between Place: Instrumental Neutron Activation Analysis of Ceramics from the Manialtepec Basin of Oaxaca” [1].

**Table:** Specifications table

Subject area	*Archaeology*
More specific subject area	*Archaeometry*
Type of data	*Maps, tables, charts, photographs*
How data was acquired	*Instrumental Neutron Activation Analysis; Elemental data was collected on high-resolution germanium detector and analyzed with Microsoft Excel and GAUSS 8.0 statistical analysis software*
Data format	*Raw and analyzed*
Experimental factors	*Sherds were cut with a Dremel, cleaned, dried, and crushed into powder for INAA*
Experimental features	*Compositional data for each ceramic sherd was collected through three gamma counts (720 seconds, 7 days, and 4 weeks).*
Data source location	*Archaeological sites in the Manialtepec Basin, Municipios of Villa de Tututepec de Melchor Ocampo and Mixtepec Distrito 22, Oaxaca, Mexico and MURR*
Data accessibility	*Data is with this article.* *Data can be downloaded from the MURR Archaeometry Laboratory Database at the following URL:*http://archaeometry.missouri.edu/datasets/datasets.html*.* *Data are organized by year (2019) and by the title of the original publication**[1]**.*
Related research article	*2019 Barber, Sarah B. and Daniel E. Pierce. “Ceramic Production and Consumption in an In-Between Place: Instrumental Neutron Activation Analysis of Ceramics from the Manialtepec Basin of Oaxaca, Mexico.” Journal of Archaeological Science:Reports 23:868-880.**[1]*

**Table:** 

**Value of the data** • Data can be used to benchmark against other coastal Oaxacan ceramics for the Classic and Early Postclassic periods to identify regional patterns of production and consumption. • Data can be compared to compositional data from other Oaxacan regions to identify clay procurement areas, ceramic production locations and practices, and local and long-distance exchange of pottery from the Late Formative to Late Postclassic periods. • Data can be used to develop further research on ceramic production in ancient Mesoamerican complex societies • Data can be compared to similar data from other world regions to reconstruct economic practices in interstitial places.

## 1 Data

These data include a map of the Manialtepec Basin showing the locations from which individual samples were collected (Fig. 1), a drawing of the stratigraphic profile from which samples obtained from an anthropogenic cut were collected (Fig. 2), photographs of sherds (Figs. A1–A70), and compositional data produced by Instrumental Neutron Activation Analysis (INAA) at the University of Missouri Research Reactor (MURR). The INAA data include a tabulation of principal components (Fig. 3; Table 1), data from log-based cluster analyses (Fig. 4), and compositional group defining discriminant analyses (Fig. 5, Fig. 6; Table 2). The data also includes bootstrapped Mahalanobis distance calculations (Appendix B).

**Fig. 1:**
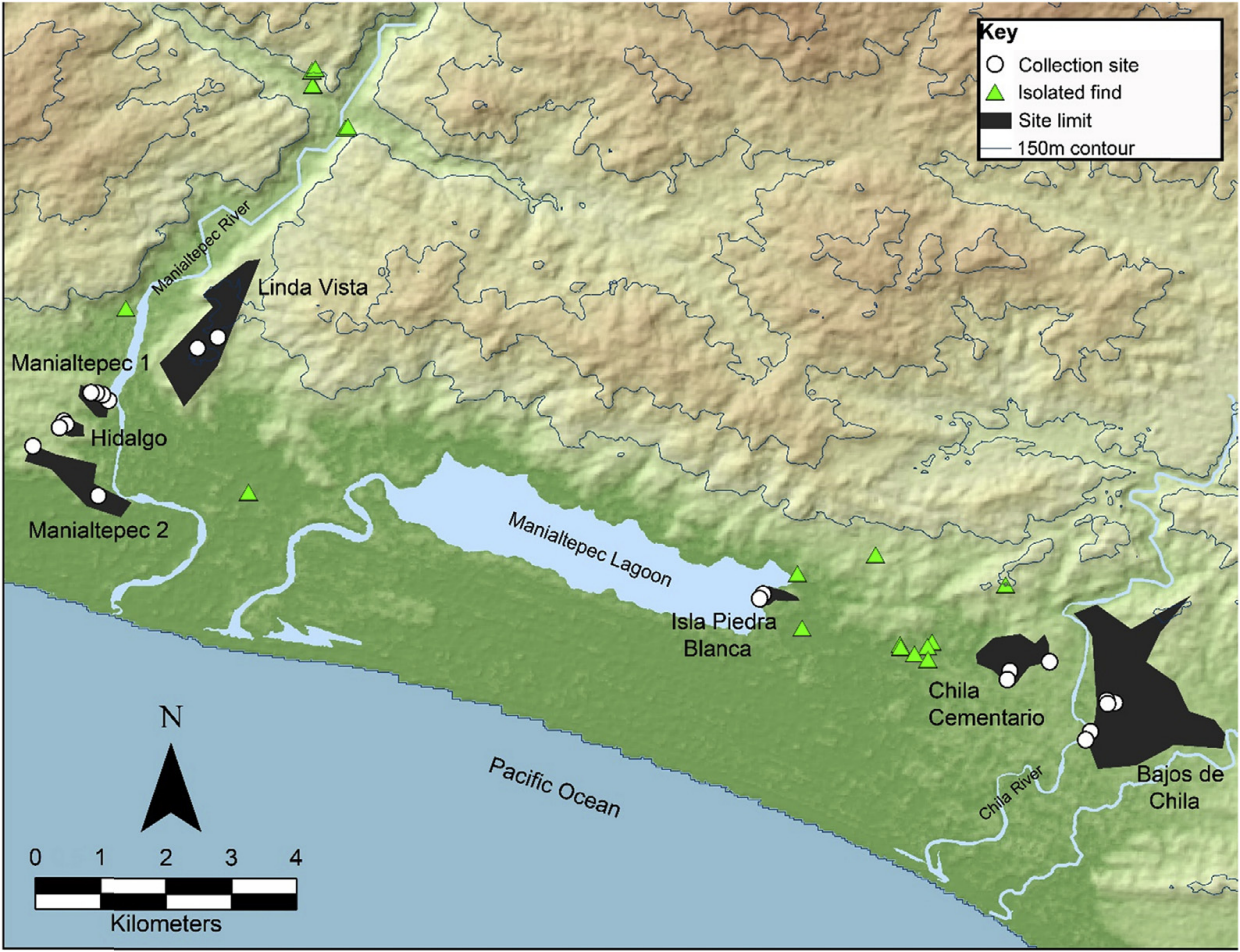
Map of the Manialtepec Basin showing the location of sites, isolated finds, and collection points for the sherds in the INAA sample.

**Fig. 2:**
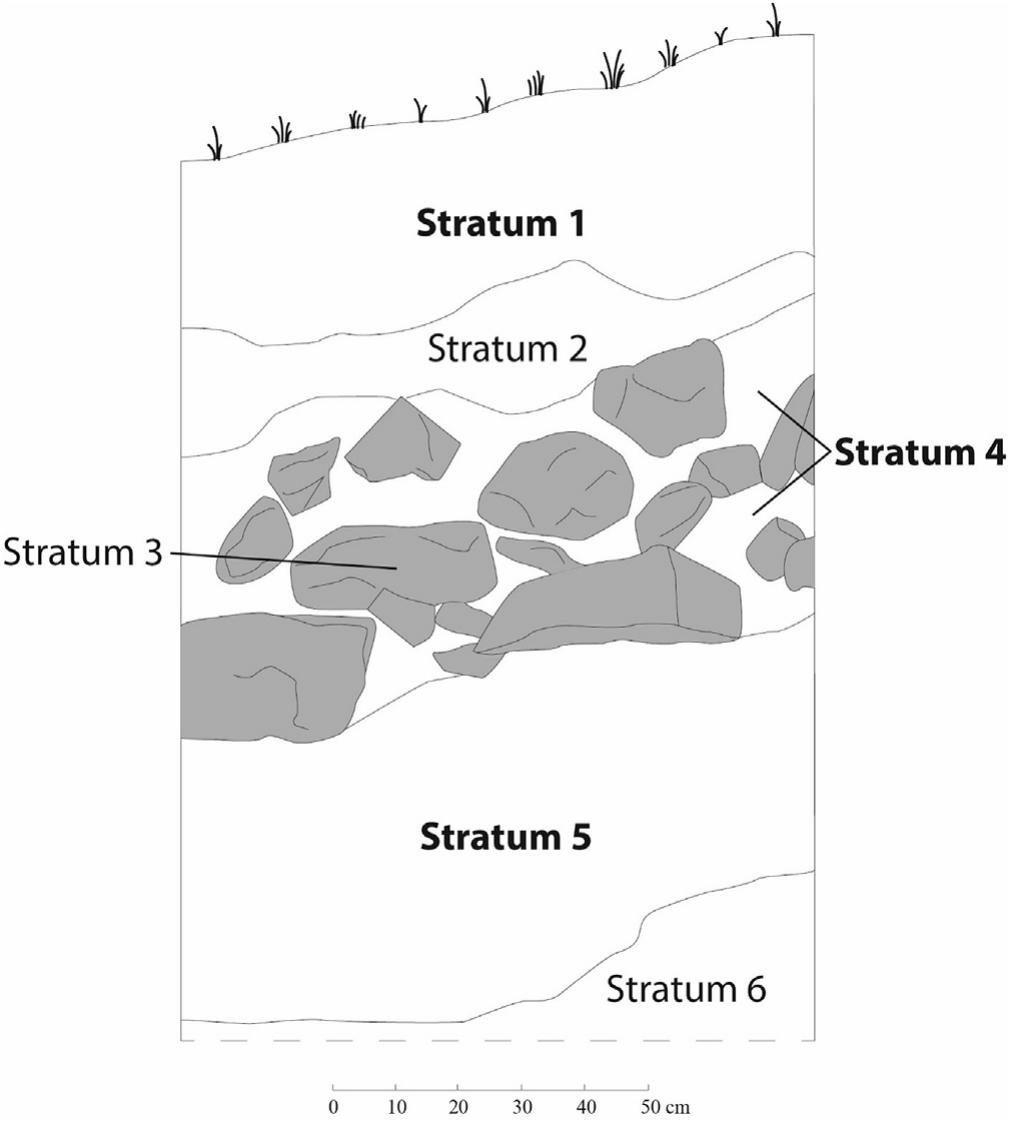
Profile of the cleaned anthropogenic cut at Bajos de Chila. Samples were derived from stratum names in **bold**. Stratum 3 is the interior course of a precolumbian retaining wall, the outer façade of which had been removed by the landowner.

**Fig. 3:**
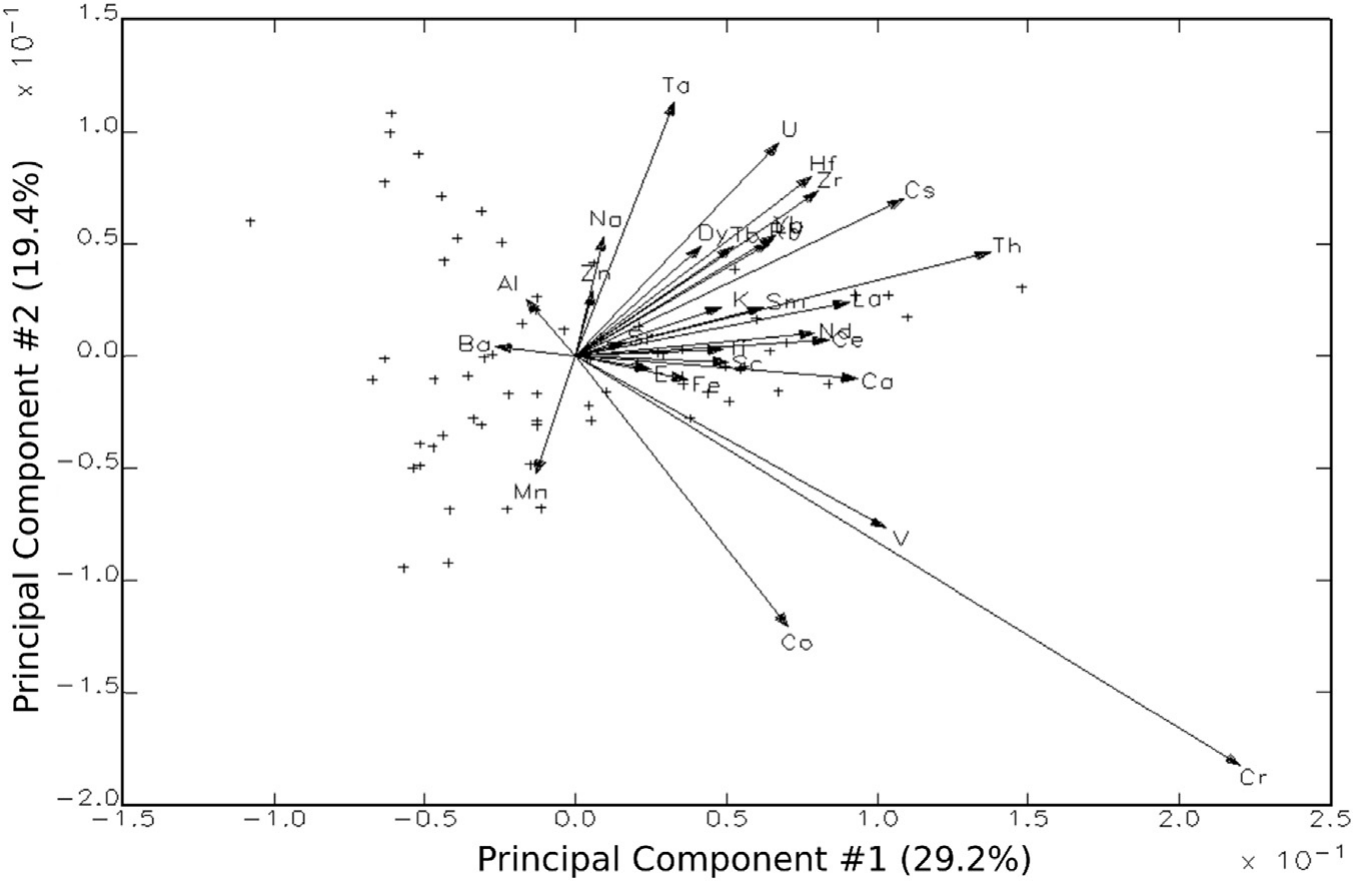
R–Q Mode biplot of the sample on principal component 1 and principal component 2.

**Table 1:** Elemental loadings for the pottery sample on principal component axes 1 through 6^a^.

Variable	Average	PC1	PC2	PC3	PC4	PC5	PC6
Ba	992.571	**−0.063**	0.013	−0.152	0.072	−0.155	−0.020
Al	100962.986	*−0.039*	0.074	−0.069	0.050	0.014	0.106
Mn	698.645	*−0.031*	*−0.154*	0.165	**0.811**	0.011	0.038
Zn	125.273	0.014	0.085	−0.085	0.182	*0.223*	0.089
Na	12122.018	0.022	0.156	*−0.337*	0.104	**−0.469**	*0.404*
Sr	327.853	0.039	0.017	**−0.450**	0.023	−0.144	0.111
Eu	1.412	0.059	−0.018	0.029	0.054	−0.033	−0.053
Ta	0.880	0.078	**0.332**	0.023	0.116	0.121	−0.013
Fe	43303.599	0.089	−0.031	−0.217	0.116	0.042	0.048
Dy	4.701	0.100	0.143	0.016	0.092	0.041	−0.109
K	17732.176	0.116	0.064	−0.064	−0.071	0.017	−0.001
Ti	4699.426	0.117	0.009	−0.226	0.070	0.151	0.012
Sc	13.819	0.121	−0.008	−0.111	0.081	0.097	−0.009
Tb	0.875	0.124	0.142	0.058	0.136	0.033	−0.028
Sm	6.818	0.149	0.064	0.071	0.037	−0.104	−0.007
Rb	80.525	0.154	0.148	0.004	0.037	0.107	0.036
Yb	2.408	0.158	0.157	0.029	0.067	0.031	−0.152
Lu	0.359	0.158	0.156	−0.008	0.063	0.037	−0.138
U	2.470	0.160	*0.278*	0.003	−0.070	0.083	**0.521**
Co	12.401	0.168	*−0.352*	0.025	*0.349*	−0.083	0.151
Hf	6.093	0.187	*0.235*	−0.175	0.063	−0.223	**−0.457**
Nd	32.011	0.190	0.030	0.106	−0.006	−0.111	−0.030
Zr	155.444	0.192	0.215	−0.162	0.084	−0.166	*−0.337*
Ce	70.496	0.200	0.021	*0.201*	0.081	−0.232	−0.047
La	33.912	0.217	0.070	0.166	−0.009	−0.208	−0.011
Ca	16209.269	0.223	−0.030	*−0.400*	−0.025	*0.226*	0.055
V	86.843	0.245	*−0.224*	−0.222	0.000	*0.256*	0.054
Cs	2.290	0.260	0.205	0.169	0.019	**0.487**	0.046
Th	8.500	*0.329*	0.136	**0.348**	*−0.112*	−0.220	*0.328*
Cr	46.445	**0.526**	**−0.535**	0.013	**−0.219**	−0.075	−0.078
Eigenvalues:		0.175	0.116	0.086	0.053	0.044	0.028
% of variation explained:		29.25%	19.45%	14.31%	8.92%	7.37%	4.65%

a Values in **bold** explain the greatest amount of variation within each component. Those in italics explain a significant portion of the variation, but less than those in bold.

**Fig. 4:**
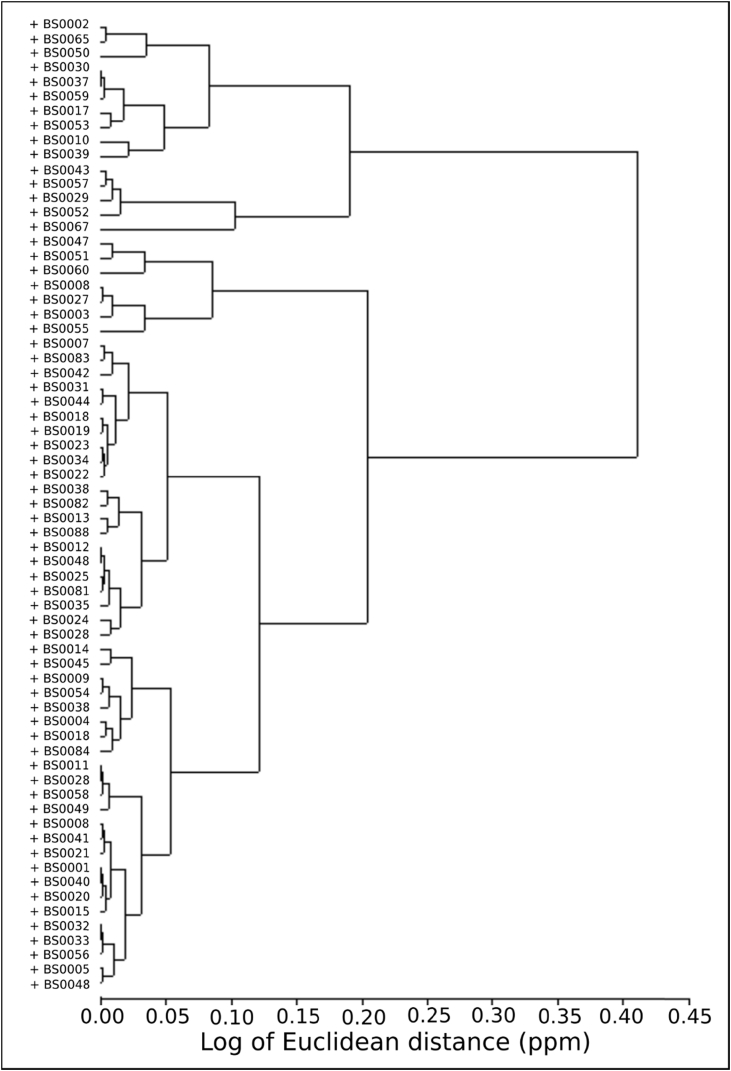
Hierarchical cluster analysis.

**Fig. 5:**
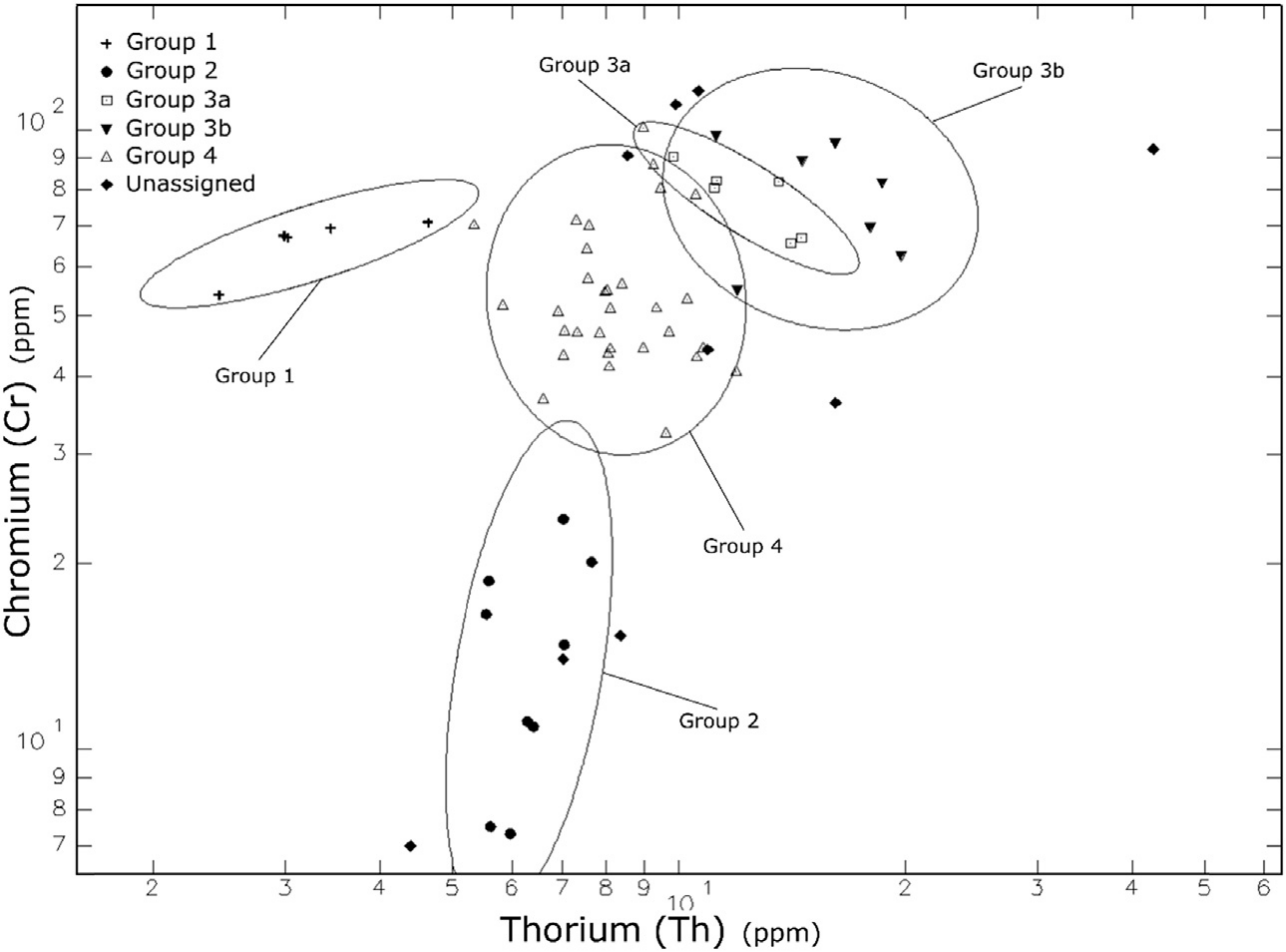
Bi-variate plot of the sample showing the chemical composition of sample on axes of Th and Cr.

**Fig. 6:**
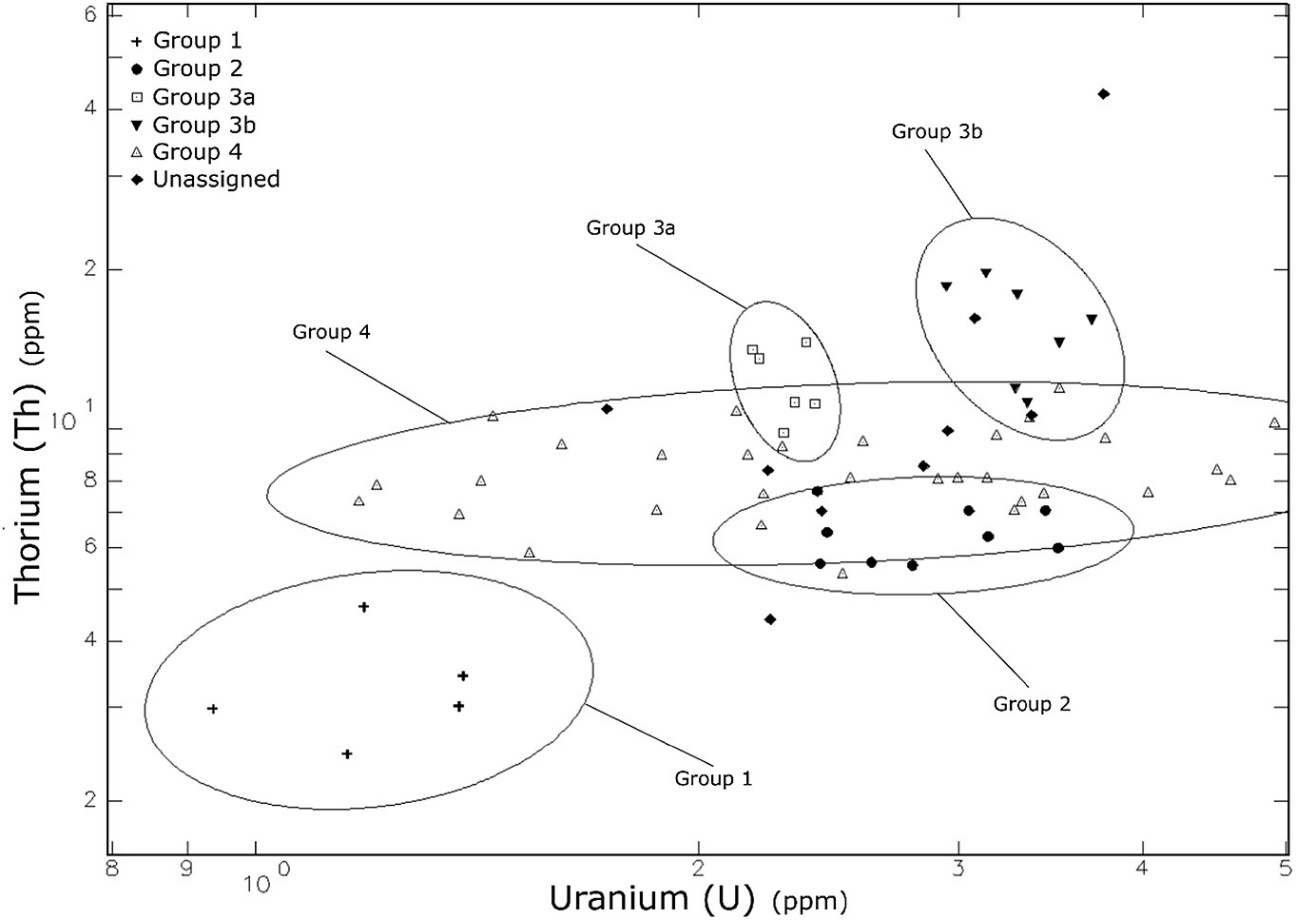
Bi-variate plot of the sample showing the chemical composition of sample on axes of U and Th.

**Table 2:** Canonical discriminant analysis of identified compositional groups in manialtepec sample.

Variable	CD1	CD2	CD3	CD4
La	1.01013	−0.49791	0.037311	−0.79517
Sm	−0.94766	0.439185	0.230063	−0.35269
Al	−0.2924	0.145029	0.960746	−0.01059
Ce	−0.36078	−0.53745	−0.52466	0.324969
Eu	0.556331	−0.53166	−0.42021	0.104332
Dy	−0.3961	0.361694	−0.0726	0.585709
Fe	0.183289	−0.68137	−0.16741	0.044277
Ta	0.053362	0.501236	0.202153	−0.43516
Lu	0.330805	0.546254	0.251123	0.087043
Yb	−0.23589	−0.57956	−0.26365	0.010992
Sc	−0.21098	−0.35812	−0.29294	−0.12941
Nd	0.101664	0.435865	−0.16689	0.196881
Cr	0.010082	0.196141	0.401964	−0.015
Ti	0.043809	−0.25669	−0.36154	0.005561
Na	−0.03503	0.242598	−0.20937	−0.03031
Cs	0.217389	0.095078	−0.04972	0.200604
Zn	−0.03008	0.227572	0.192117	−0.00972
K	0.0658	−0.25037	−0.13574	−0.05636
Rb	−0.19079	−0.08245	−0.1731	−0.11536
Th	0.146437	0.089392	−0.00248	0.208651
Tb	0.155683	0.138913	0.134585	−0.10172
Ba	−0.05615	0.138325	−0.02458	0.158131
Ca	−7.7E-05	0.041462	0.193157	−0.07456
Sr	0.123788	−0.10937	−0.11089	−0.00491
Co	0.127855	−0.111	−0.10133	−0.00744
Hf	−0.14991	0.021014	0.073442	0.086472
Mn	−0.04666	0.101769	0.099362	−0.00932
U	0.034065	0.038021	0.008713	0.106311
Zr	0.040996	−0.05674	0.035313	0.083834
V	−0.0686	0.001351	−0.0157	0.068594
Total variance explained:	53.58%	33.73%	11.11%	1.57%
		Wilk's lambda:		0.000227
		Approx. F:		5.924415
		p-value:		1.1E-17

## 2 Experimental design, materials, and methods

### 2.1 Research area

The materials for this data set consisted of 66 ceramic sherds from seven archaeological sites in the Manialtepec Basin of Pacific coastal Oaxaca, Mexico. The Manialtepec Basin is a 60 km^2^ coastal basin surrounded by the piedmont zone of the Sierra Madre del Sur mountain range and includes a 1200-ha lagoon. Two permanent rivers flow into the basin on either side of the lagoon: the Manialtepec River on the west and the Chila River on the east. Archaeological surface reconnaissance of the basin has identified 21 archaeological sites and 16 isolated surface finds of precolumbian materials dating from the Late Formative (400–150 BCE) to Late Postclassic periods (CE 1100–1522) (Fig. 1) [1]; see also [2]. Only four of these sites were larger than 25 ha in area: Bajos de Chila (288 ha), Linda Vista (115 ha), Chila Cementerio (62 ha), and Manilatepec 2 (57 ha).

### 2.2 Sample selection

Sample sherds were selected based on chronological sensitivity or because they appeared to be non-local to the Oaxaca coast. Sherds diagnostic of all precolumbian time periods, based on the published ceramic chronology of the lower Río Verde valley [3], [4], [5], were included. Later Formative (400 BCE – CE 250) and Late Postclassic samples were emphasized because there was a large extant database for these two time periods in the literature on Oaxacan paste composition groups. All samples were recovered either from surface collections (*n* = 50; 76%; see Fig. 1) or a cleaned and documented anthropogenic cut through precolumbian architecture at Bajos de Chila (*n* = 16; 24%; Fig. 2).

### 2.3 Sample preparation for INAA

In accordance with MURR protocols for INAA sample preparation [6], [7], [8], [9], 1 cm^2^ fragments were removed from each specimen using a silicon carbide burr. This removed all glaze, slip, paint, and adhering soil, thus minimizing the risk of erroneous measurement of contaminants. After removal, specimens were washed in deionized water and dried. To homogenize the specimens, each sherd fragment was then ground into a fine powder using an agate mortar and pestle and split into multiple analytical samples. When possible, a portion was archived for future research, while two analytical samples were retained for each specimen. Each sample was weighed to the nearest 0.01 mg using an analytical balance. One 150 mg sample of powder was weighed and sealed into a clean high-density polyethylene vial. A second 200 mg sample was then sealed into a high-purity quart vial. While the first was used for shorter irradiation, the second was reserved for longer periods of irradiation. National Institute of Standards and Technology (NIST) certified standards, including SRM-1633a (coal fly ash) and SRM-688 (basalt rock) were included for reference. Treating them as unknowns, SRM-278 (obsidian rock) and Ohio Red Clay were also utilized to ensure data quality.

After preparation, the samples were irradiated and subjected to three subsequent gamma counts. Each polyvial was sequentially irradiated through a pneumatic tube system two at a time for 5 s by a neutron flux of 8 × 10^13^ n cm^−2^ s^−1^ [6]. After this short irradiation, a gamma count of 720 seconds yielded spectra containing peaks for nine short-lived elements: aluminum (Al), barium (Ba), calcium (Ca), dysprosium (Dy), potassium (K), manganese (Mn), sodium (Na), titanium (Ti), and vanadium (V). The 200 mg samples in quartz vials were then subjected to a long 24-h irradiation at a neutron flux of 5 × 10^13^ n cm^−2^ s^−1^ and allowed to decay for seven days. Decay was recorded through gamma counts of 1800 seconds using a high resolution geranium detector coupled to an automatic sample changer. This count allows the recordation of seven medium half-life elements, including: arsenic (As), lanthanum (La), lutetium (Lu), neodymium (Nd), samarium (Sm), uranium (U), and ytterbium (Yb). Finally, an additional count of 8,500 seconds was recorded after an additional four weeks of decay to yield measurements of 17 long half-life elements, including: cerium (Ce), cobalt (Co), chromium (Cr), cesium (Cs), europium (Eu), iron (Fe), hafnium (Hf), nickel (Ni), rubidium (Rb), antimony (Sb), scandium (Sc), strontium (Sr), tantalum (Ta), terbium (Tb), thorium (Th), zinc (Zn), and zirconium (Zr). Subsequently, data from all three counts were tabulated in parts per million.

### 2.4 Statistical analysis of INAA data

Interpretation of compositional data obtained from INAA included an array of statistical procedures discussed elsewhere [[6], [9],[10], [11], [12], [13]]. In total, the gamma counts produced elemental concentration values for 33 elements. Nickel (Ni) and Arsenic (As), however, were removed from statistical analyses due to a high number of instances where concentrations fell below detection limits. All statistical analyses were then carried out on base-10 logarithms to account for differences in elemental magnitude through GAUSS 8.0 software. The dataset was initially characterized using primarily principal component analyses (PCA) (Fig. 3 and Table 1).

This information was then used in coordination with hierarchical cluster analysis (HCA) (Fig. 4), visual inspection of bivariate plots (Fig. 5, Fig. 6), and canonical discriminant analysis (CDA) (Table 2; see also [1]: Fig. 3]) to determine compositional groups. Bootstrapped multi-dimensional Mahalanobis distance was then calculated using principal components in adherence to the provenance postulate [14] (Appendix B). After discrimination of compositional groups, data was then compared to archived samples within MURR's NAA database through visual inspection of bivariate plots (see Ref. [1]) and Euclidian distance searches using to assess potential provenance locales.
