# Supplementary material for: Supplemental paste composition data from the Manialtepec Basin, Oaxaca
Source: Data Brief. 2019 Mar 8;23:103805. doi: 10.1016/j.dib.2019.103805 (PMC6661237; doi:10.1016/j.dib.2019.103805)
Supplement: Multimedia component 2 [file mmc2.docx]

**Appendix A**

**Photographs of 58 sherds from the Manialtepec Basin analyzed using INAA**

**
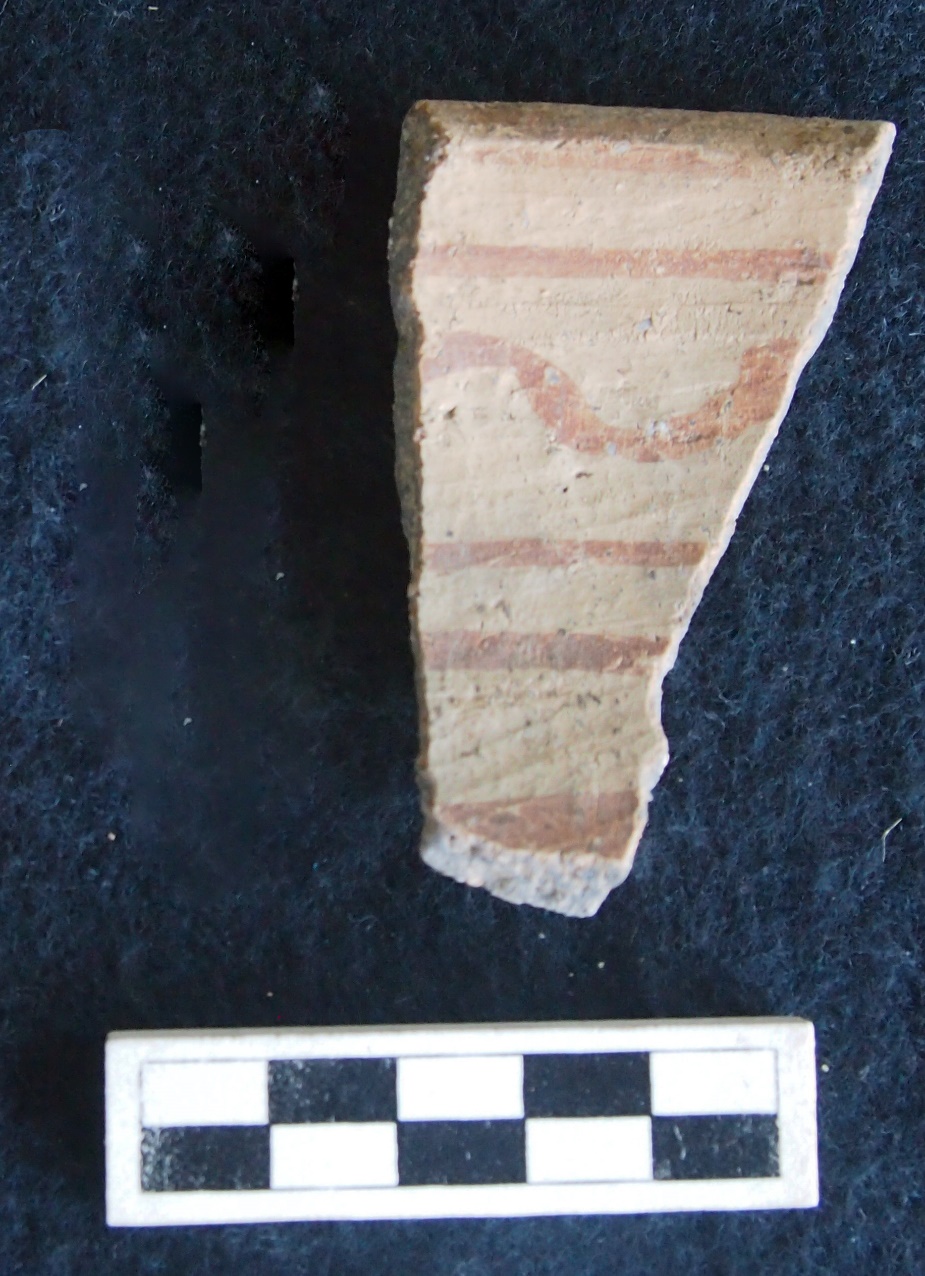
**

Appendix A.1: BS0002 (see [1], Fig. 2D for image of BS001)

**
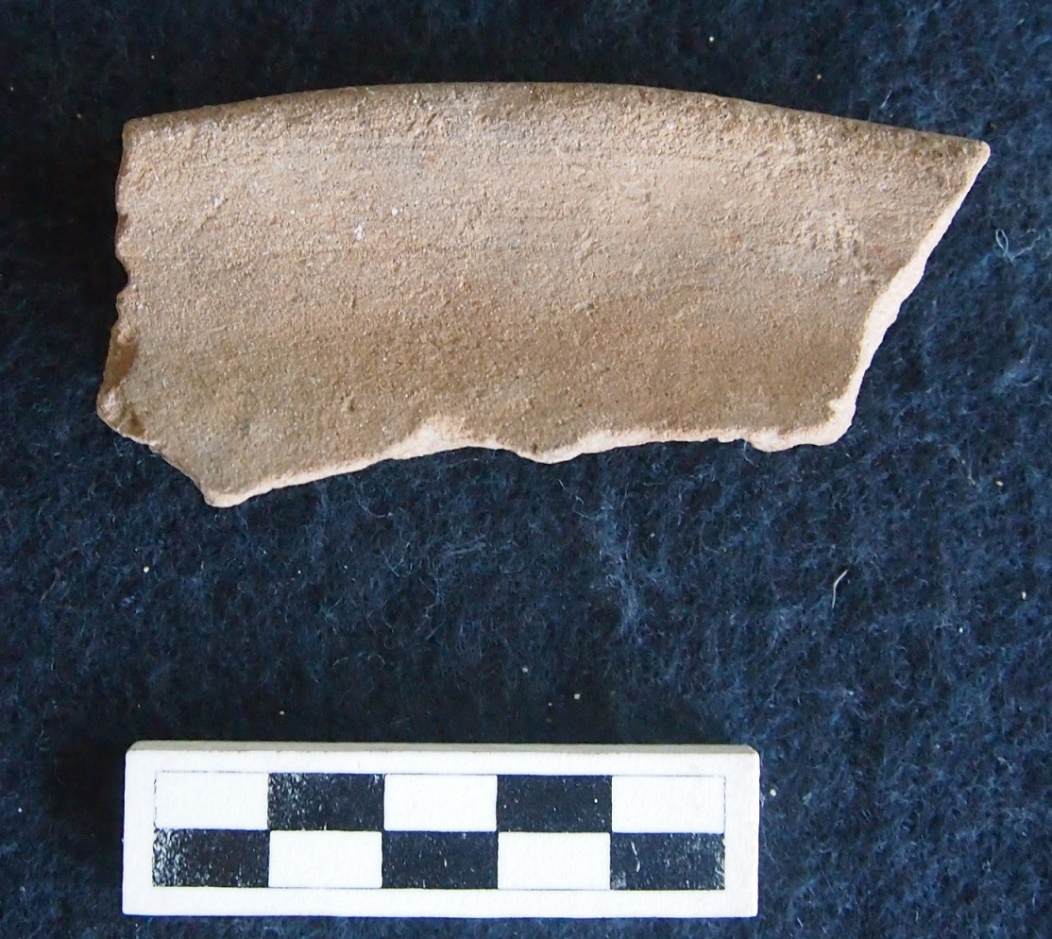
**

Appendix A.2: BS0003 Interior


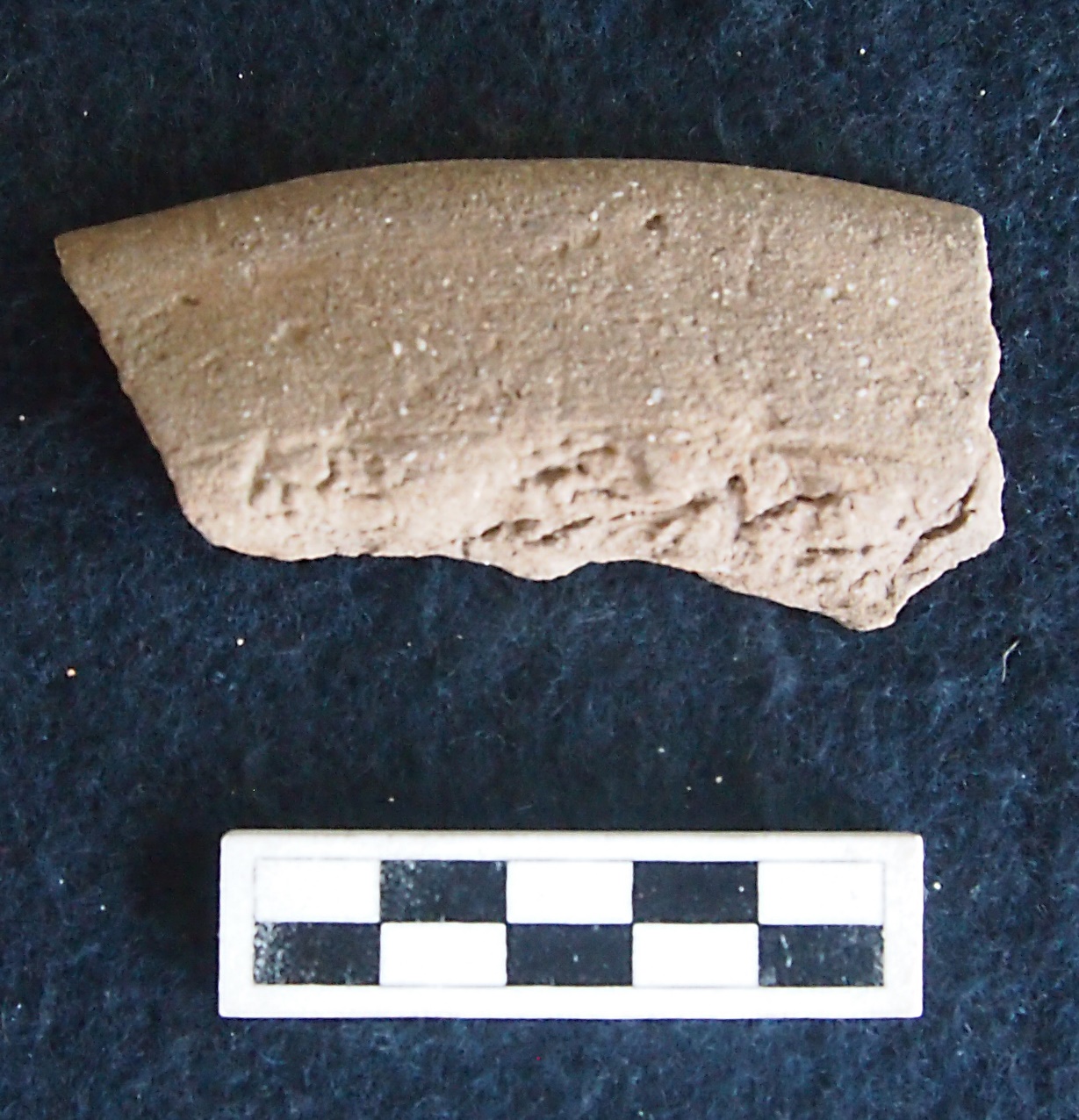


Appendix A.3: BS0003 Exterior


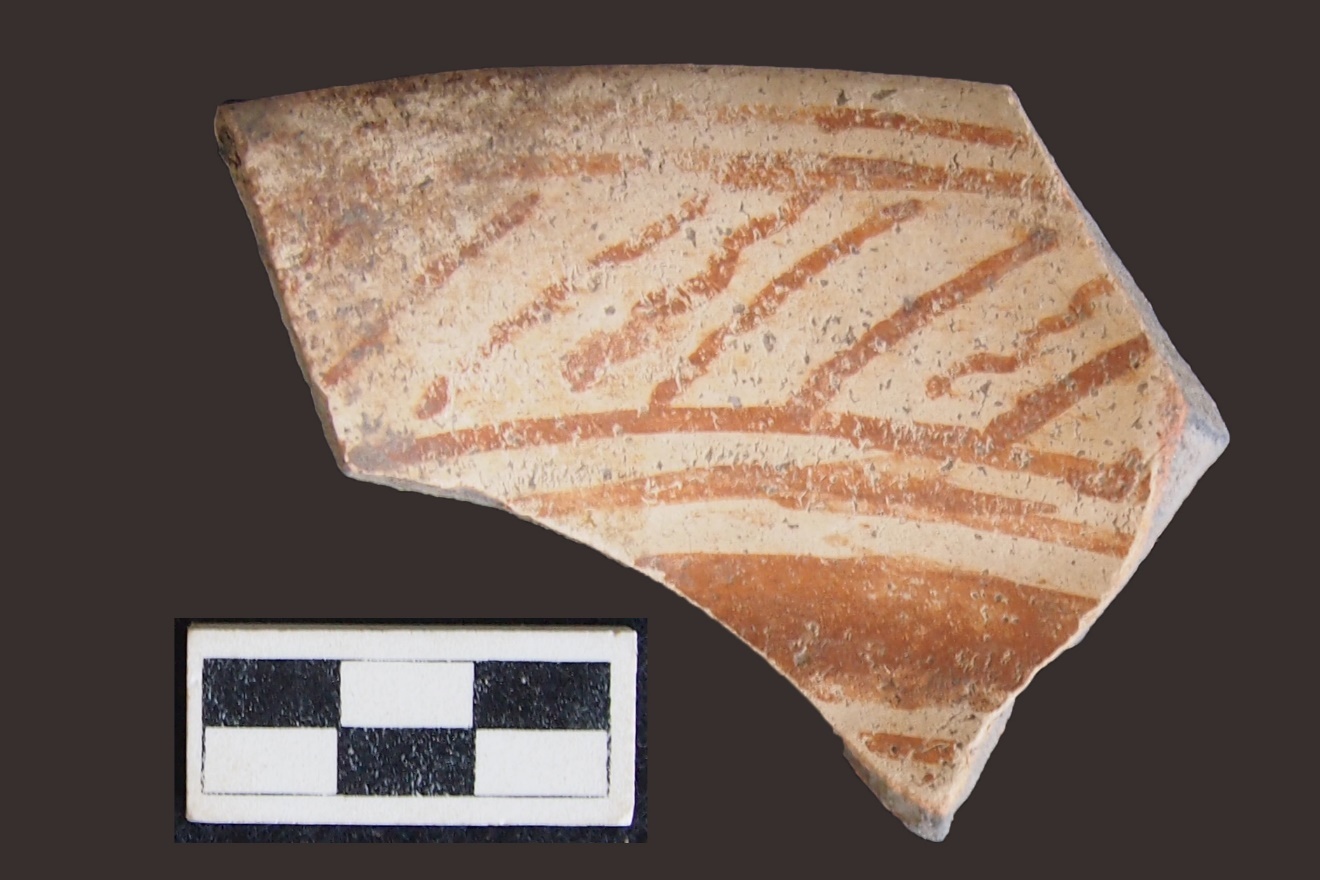


Appendix A.4: BS0004


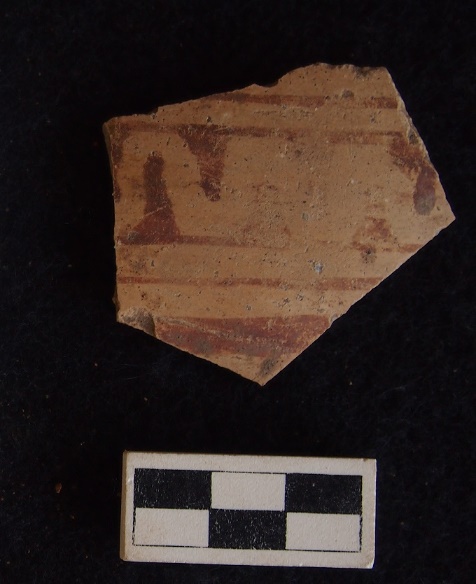


Appendix A.5: BS0005


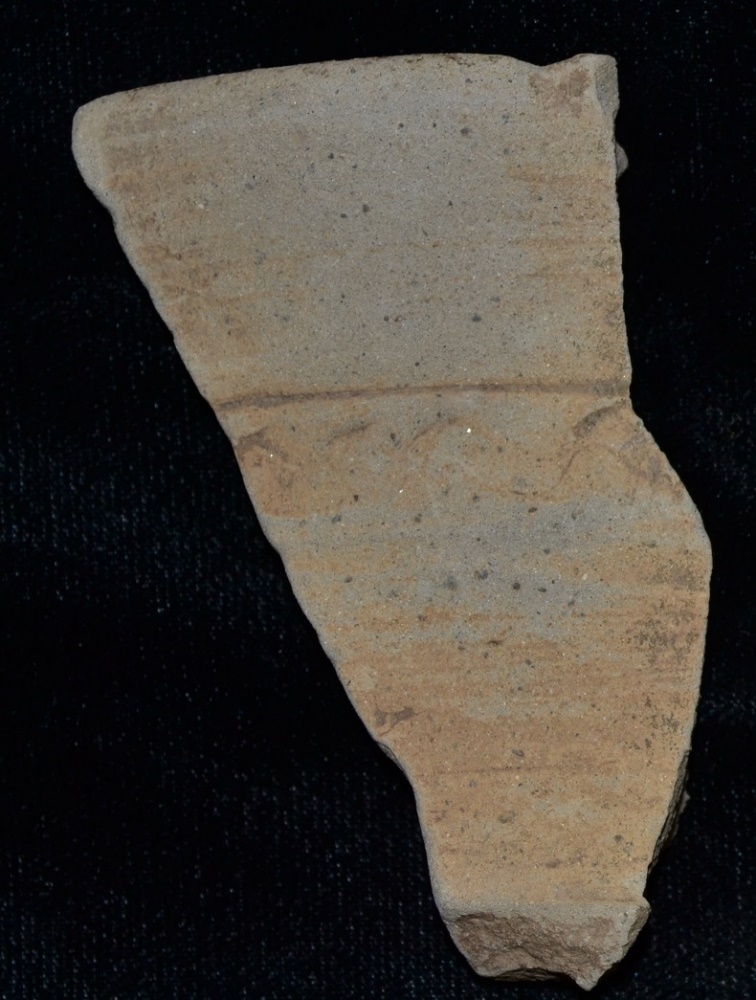


Appendix A.6: BS0006


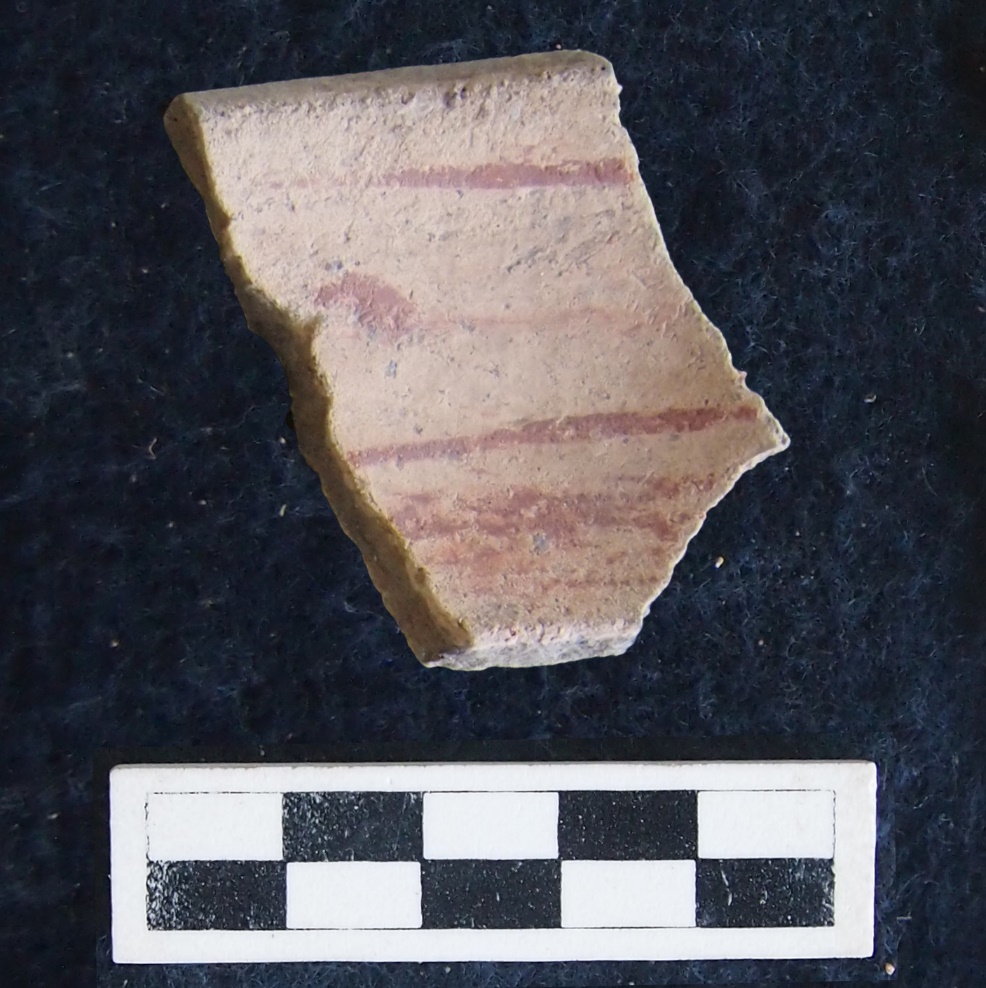


Appendix A.7: BS0007


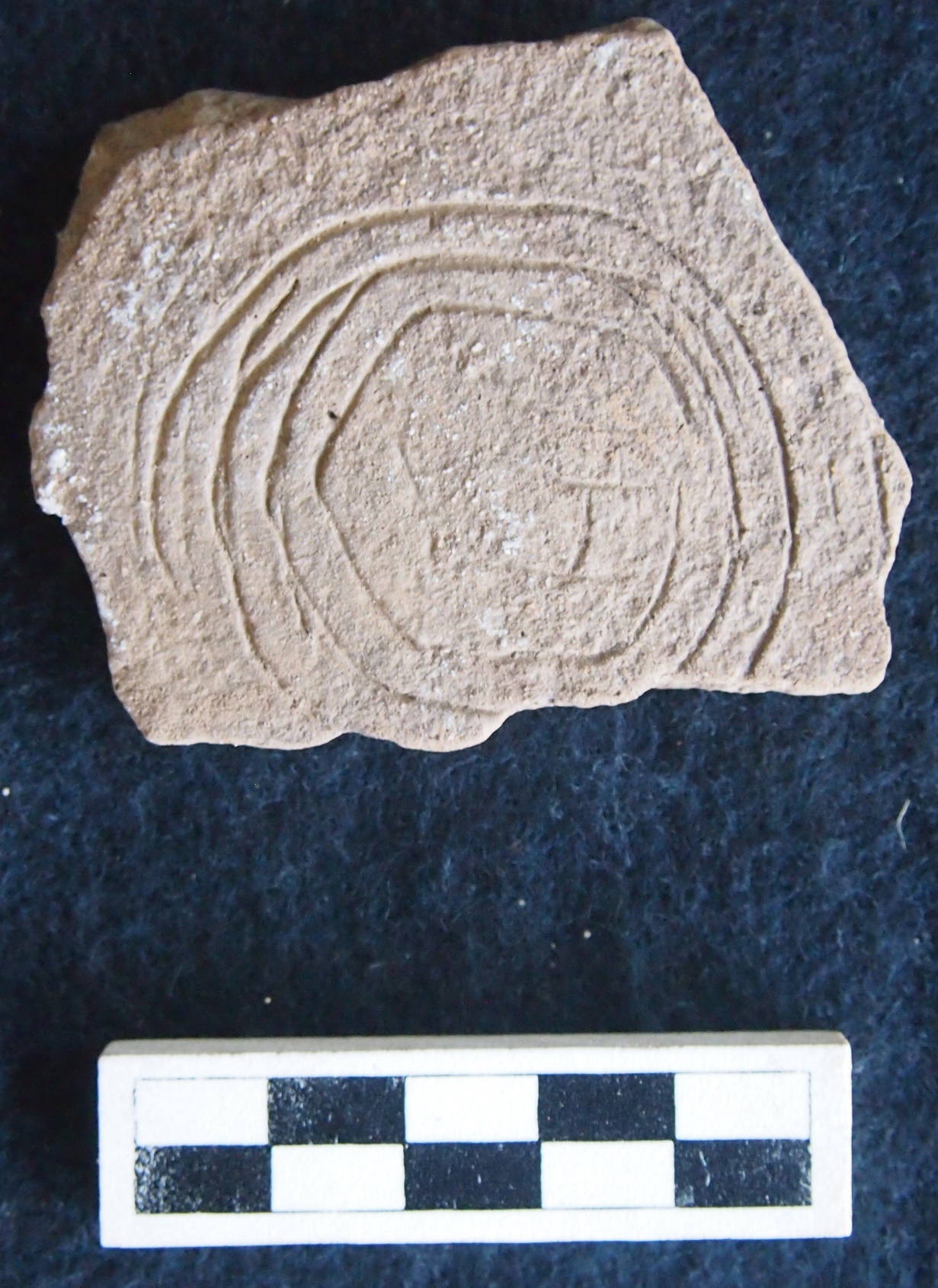


Appendix A.8: BS0008


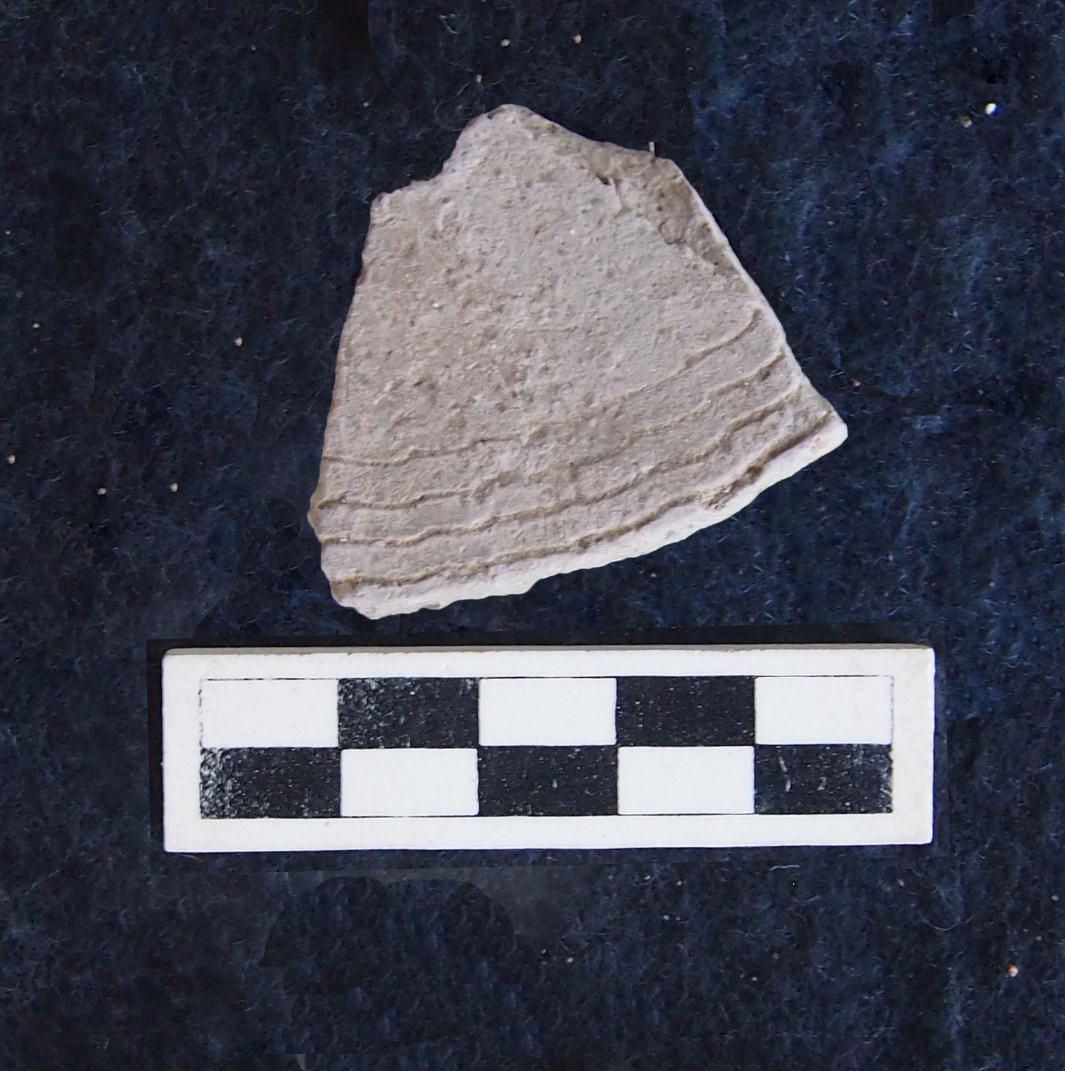


Appendix A.9: BS0009


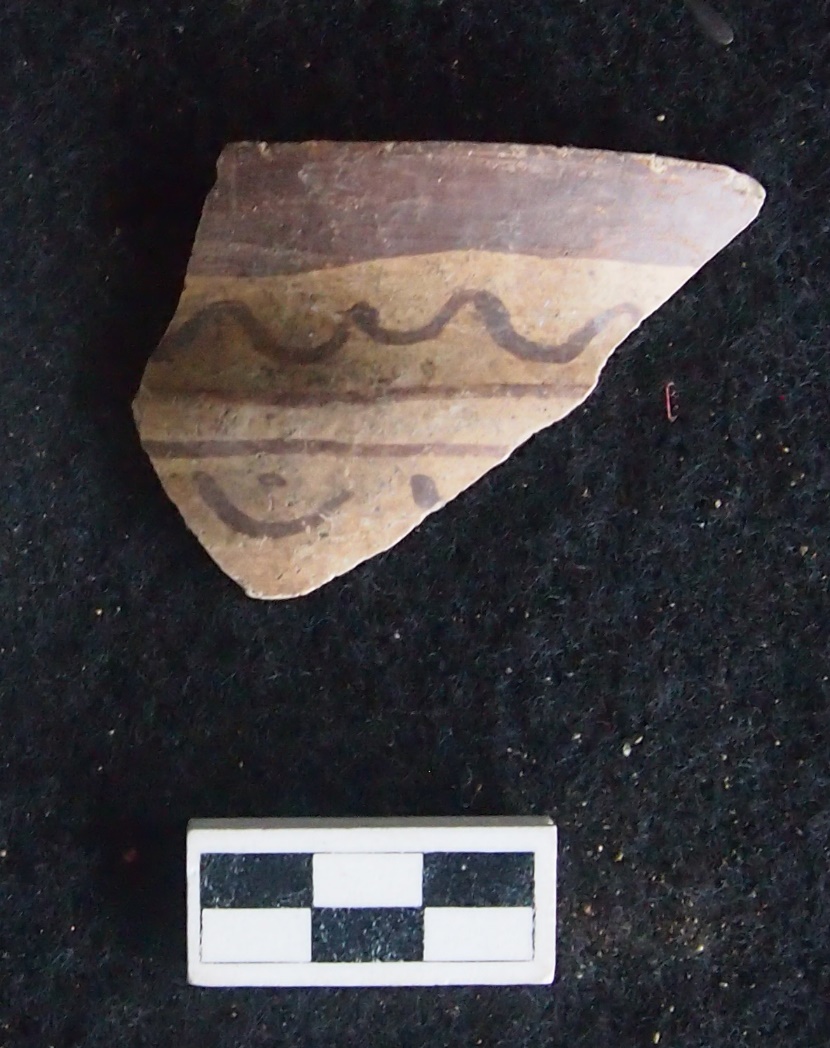


Appendix A.10: BS0010


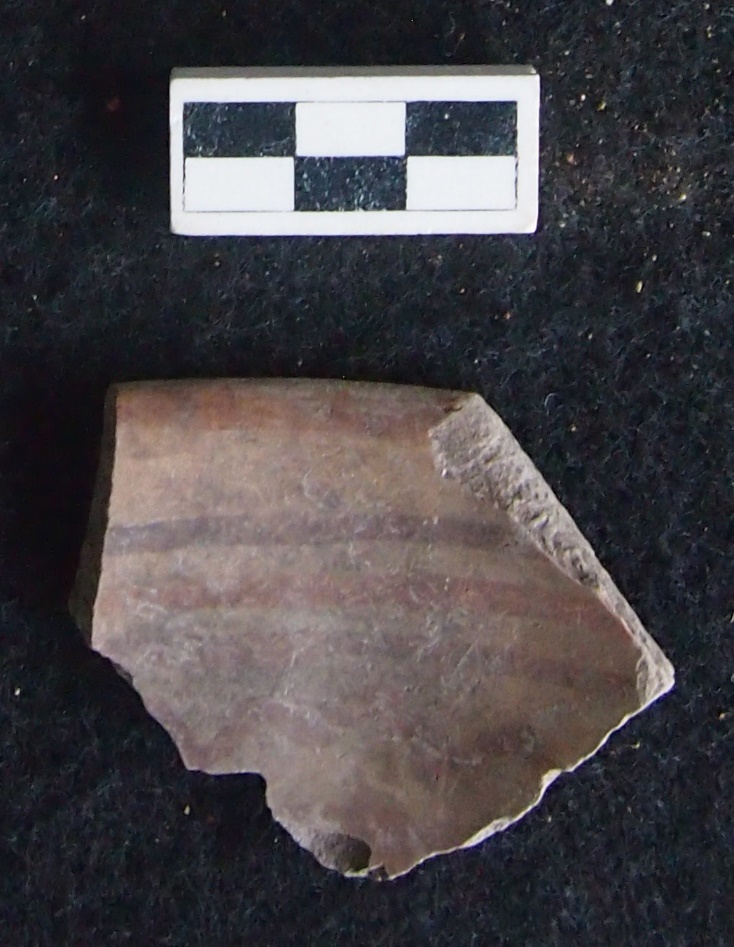


Appendix A.11: BS0011


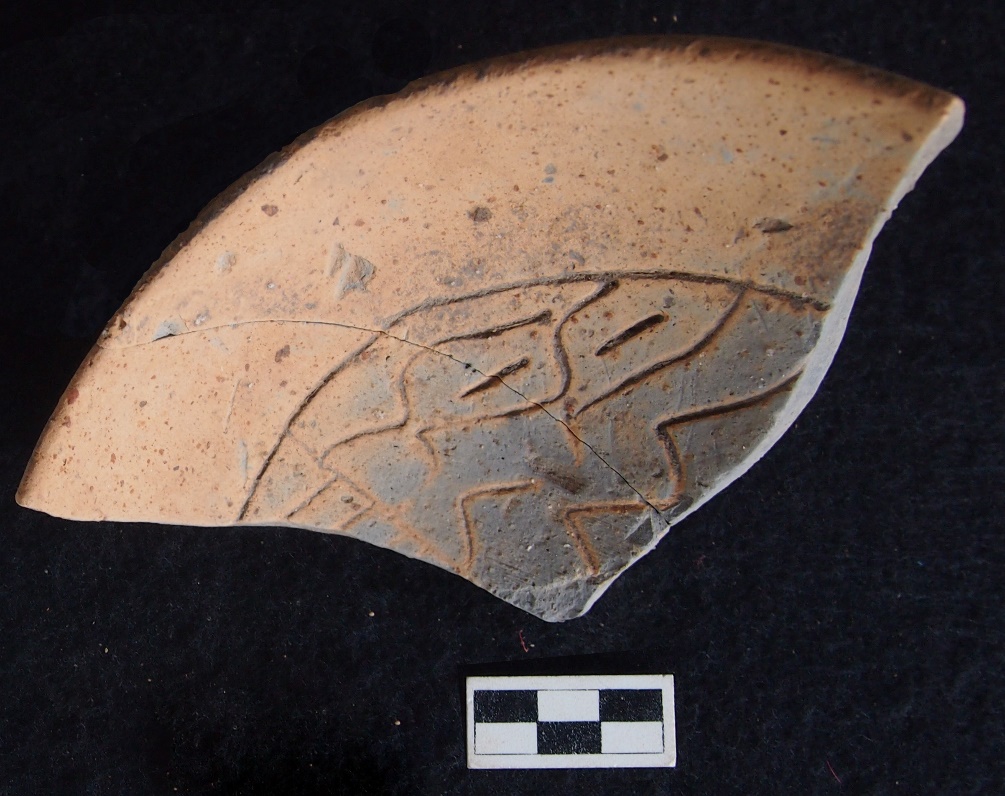


Appendix A.12: BS0012


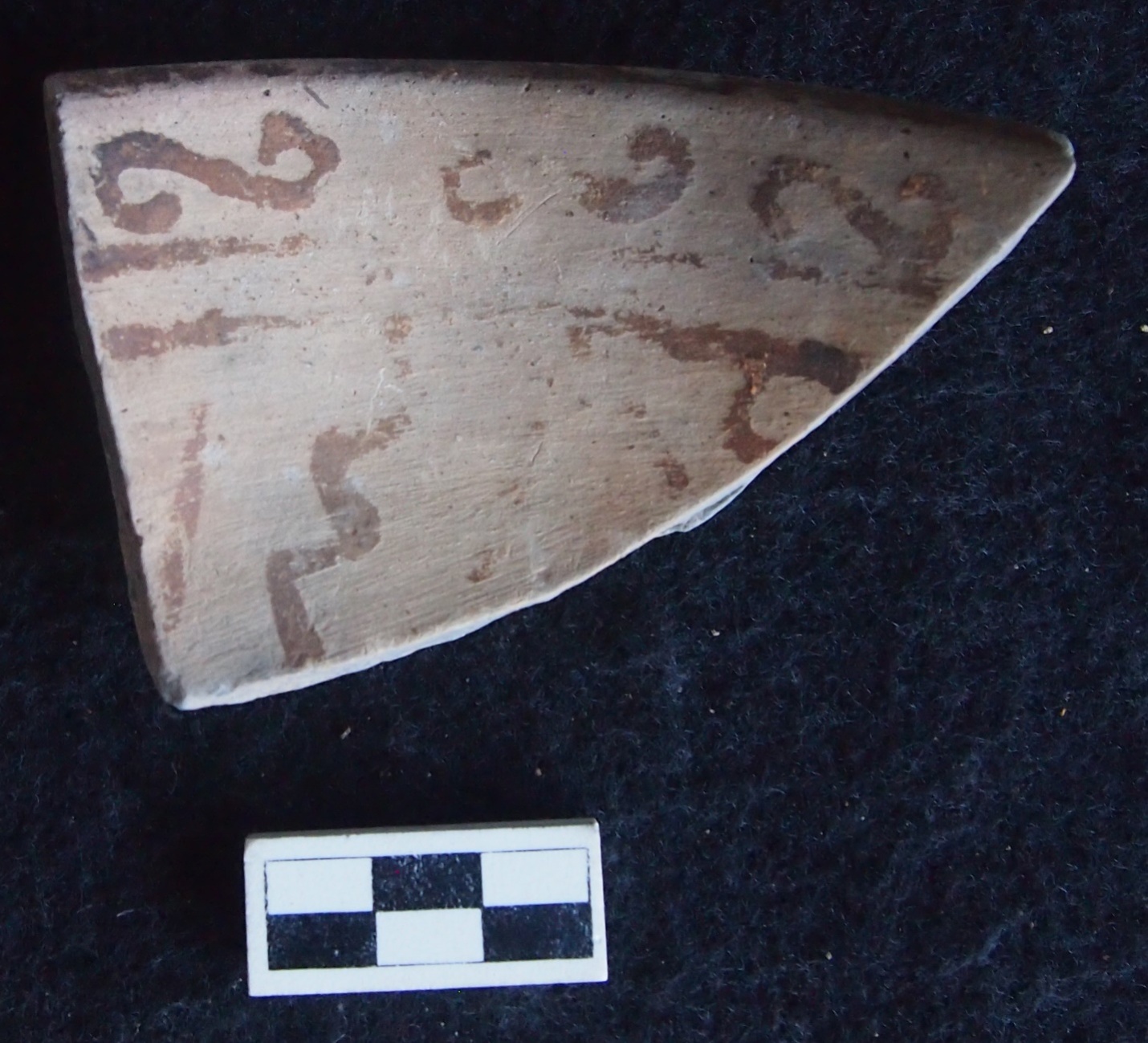


Appendix A.13: BS0013


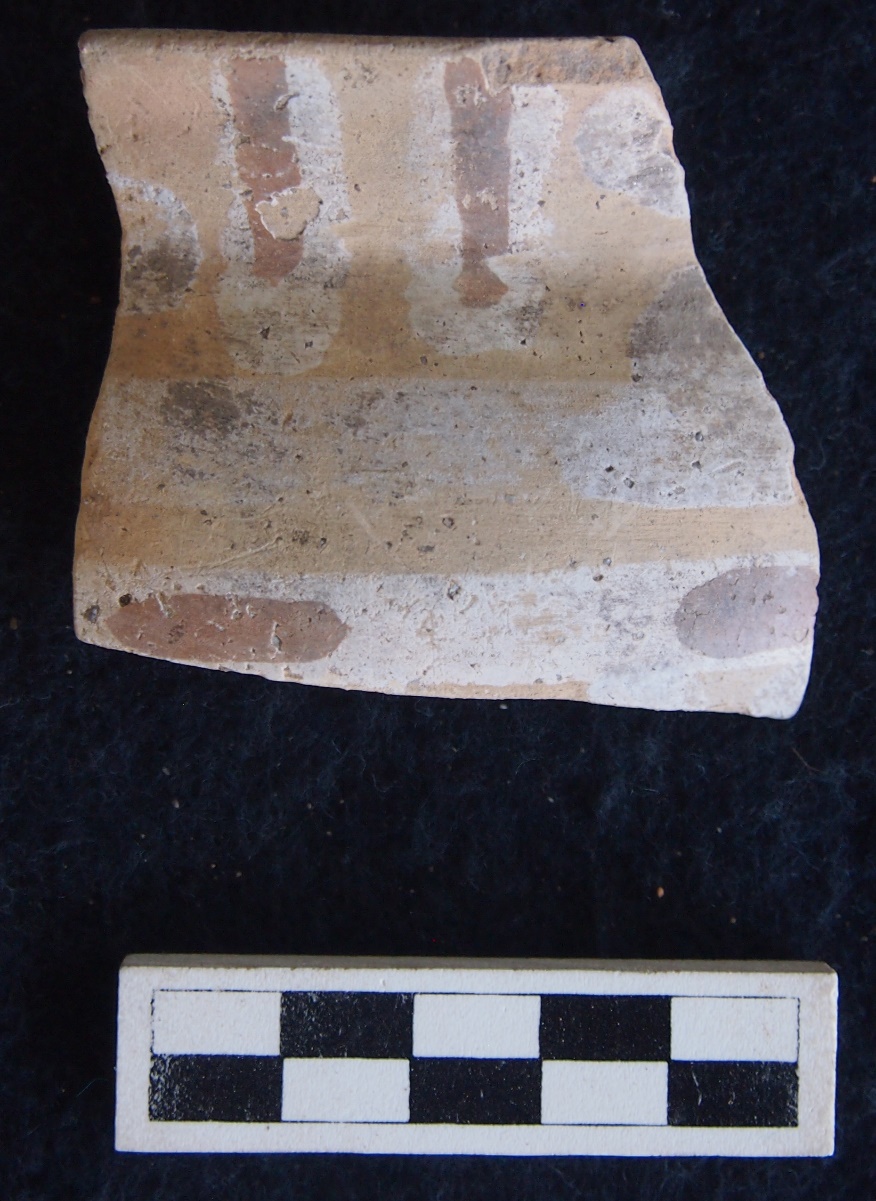


Appendix A.14: BS0014


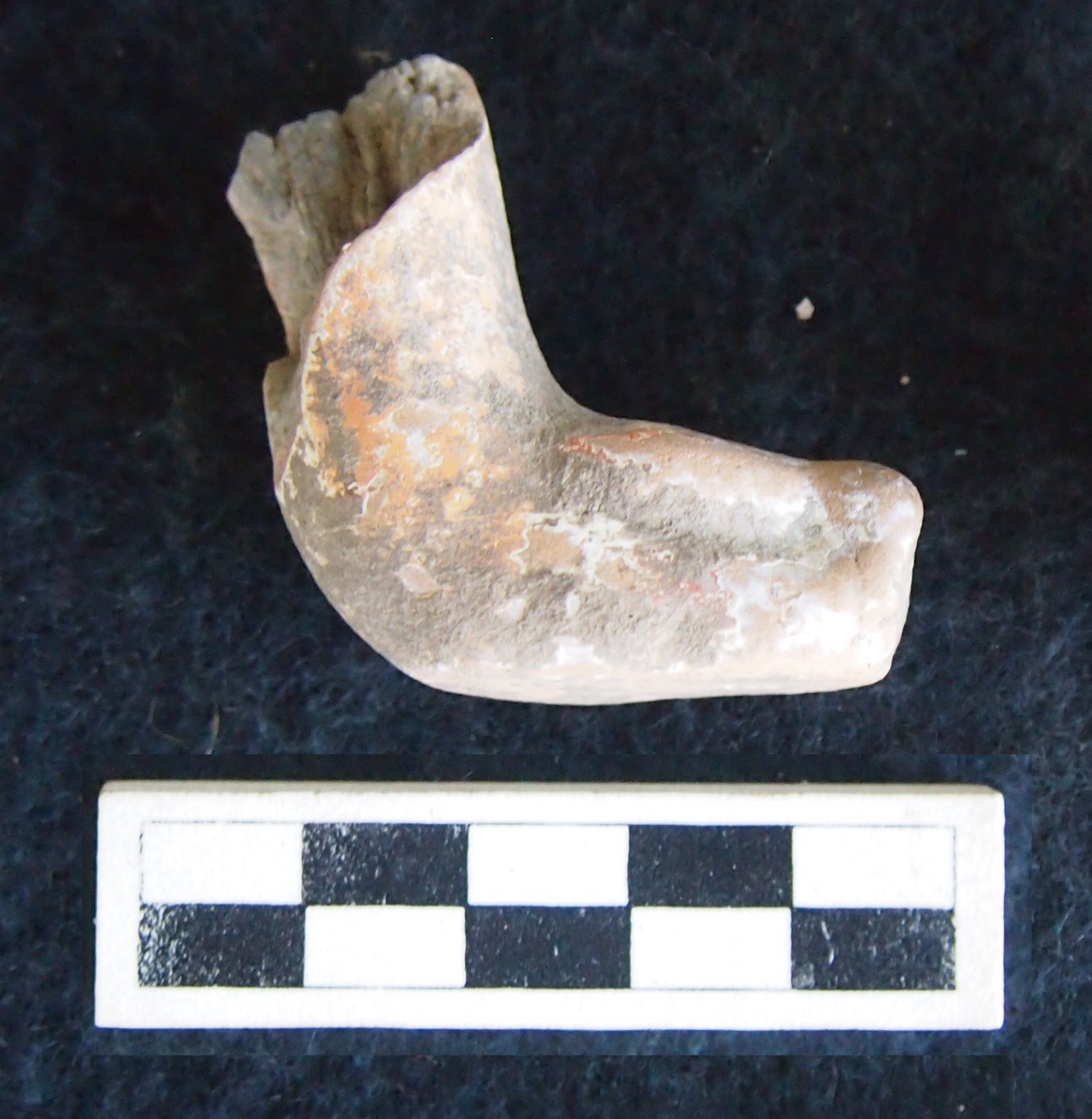


Appendix A.15: BS0015


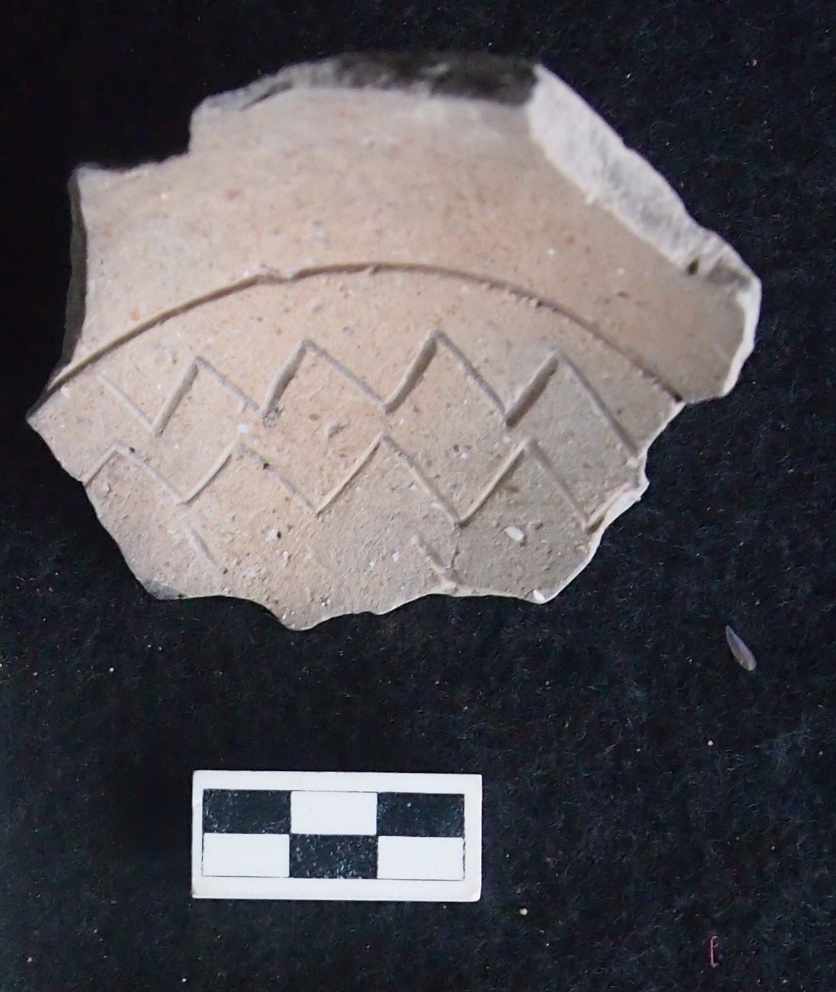


Appendix A.16: BS0016


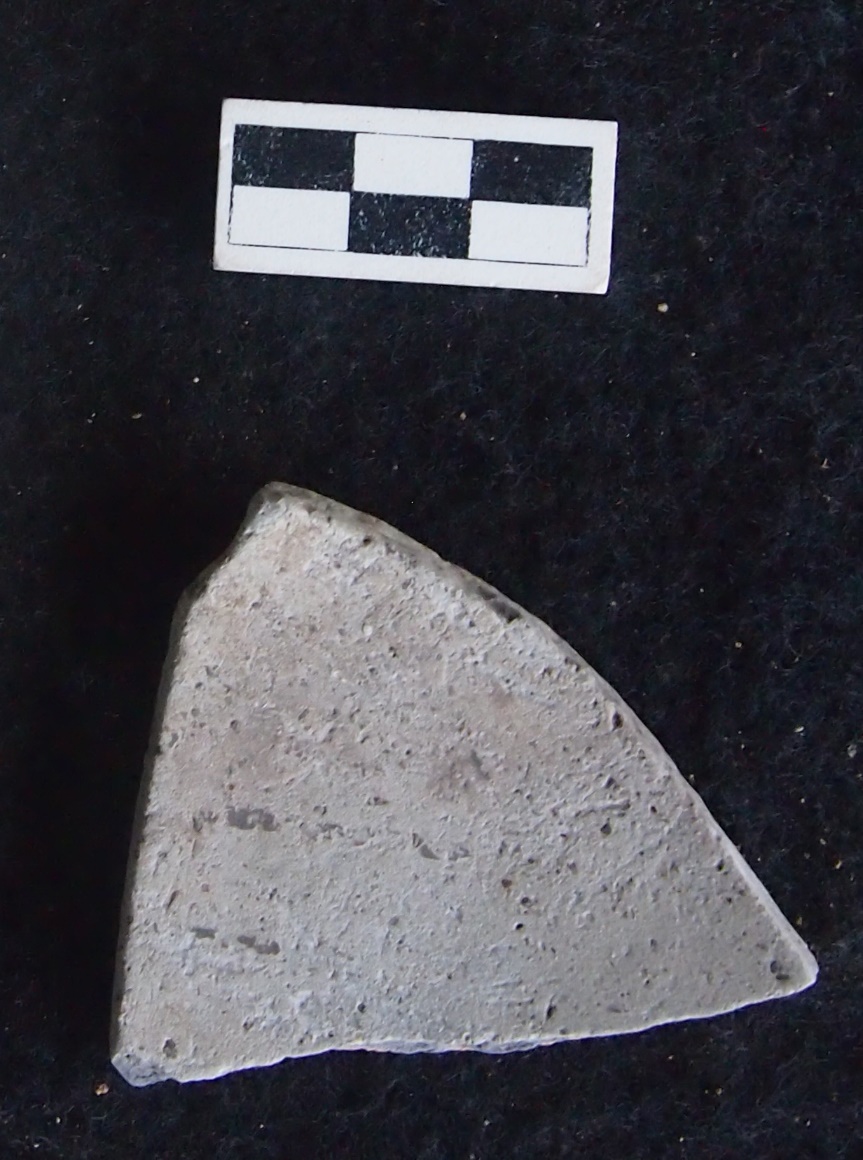


Appendix A.17: BS0017 interior


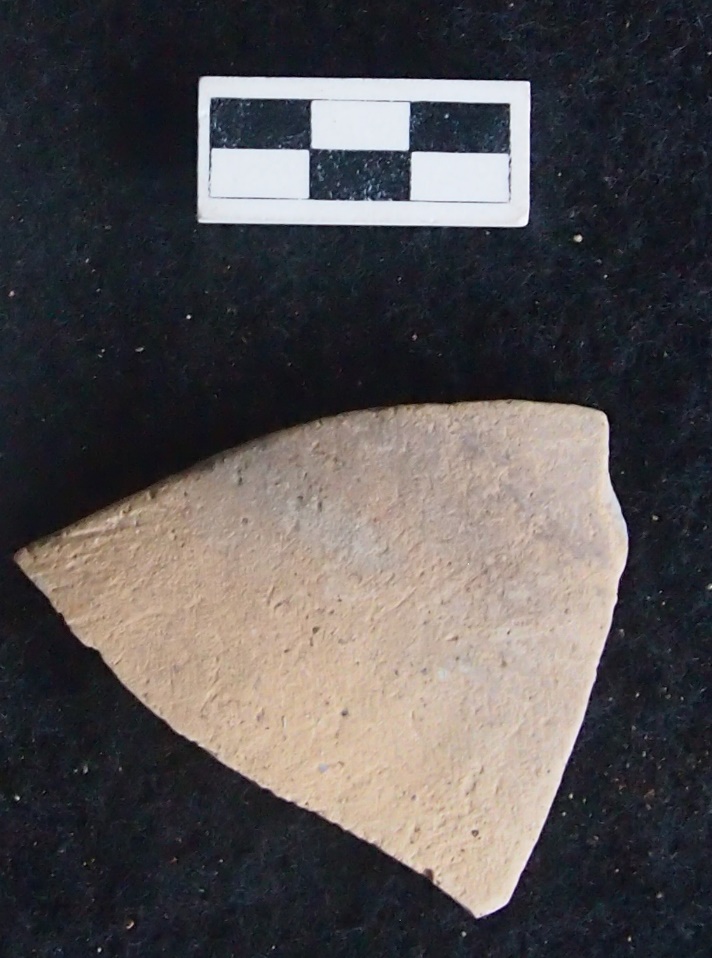


Appendix A.18: BS0017 exterior

**
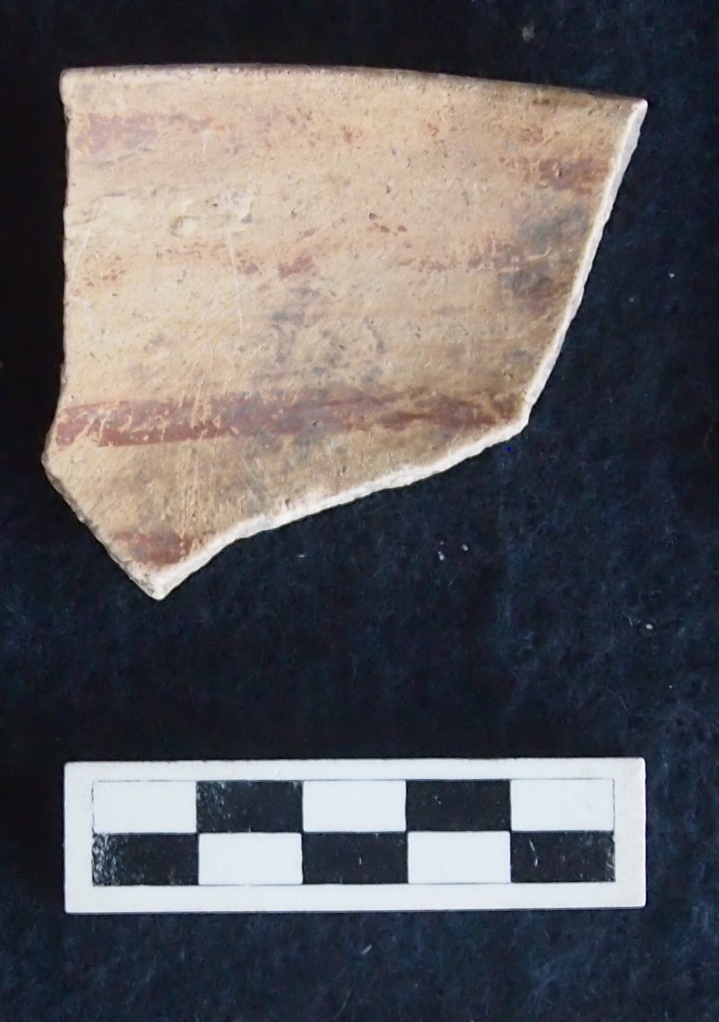
**

Appendix A.19: BS0018


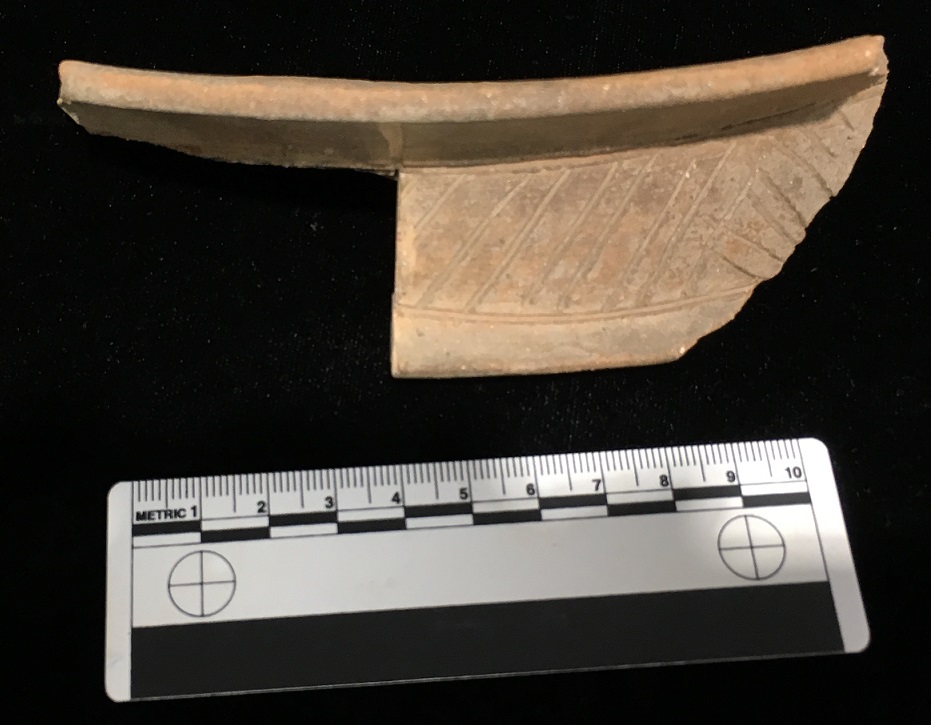


Appendix A.20: BS0019


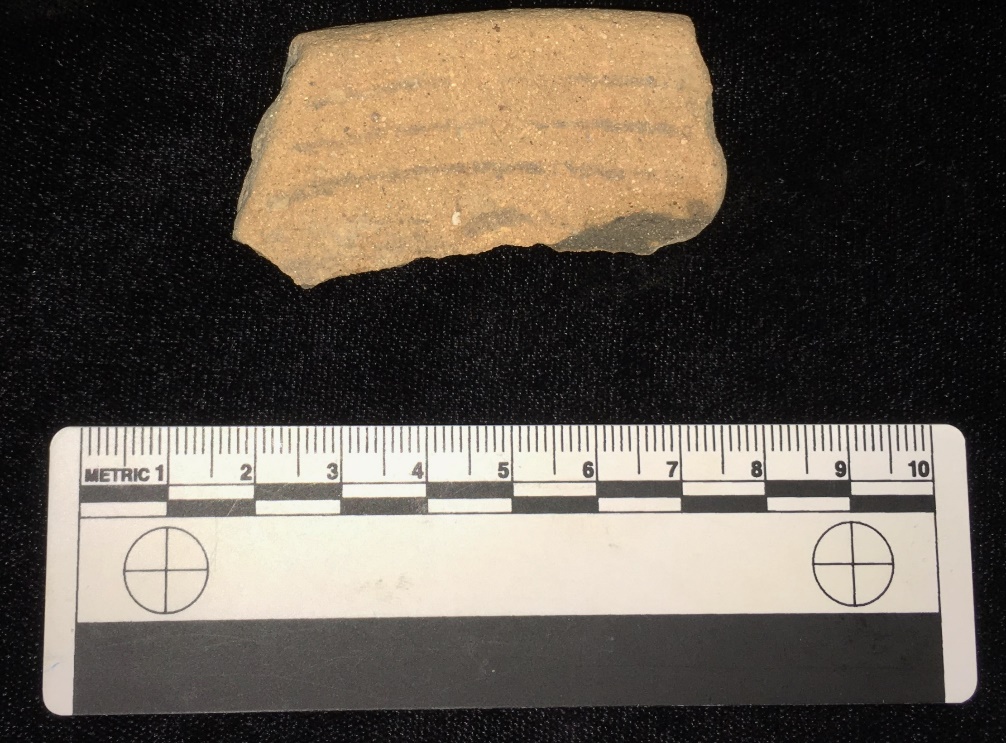


AppendixA.21: BS0020


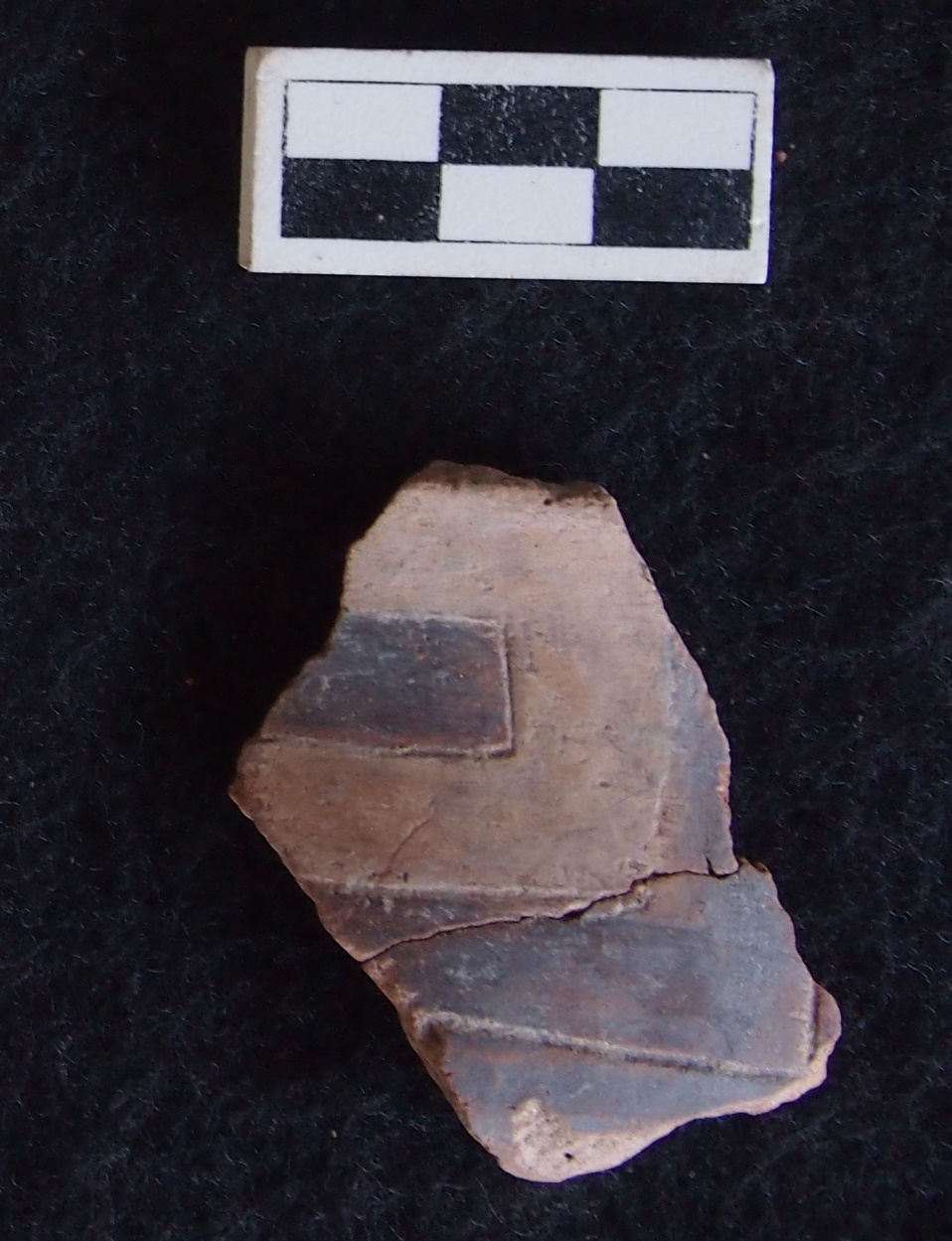


AppendixA.22: BS0022(see [1],Fig. 6B for image of BS0021)


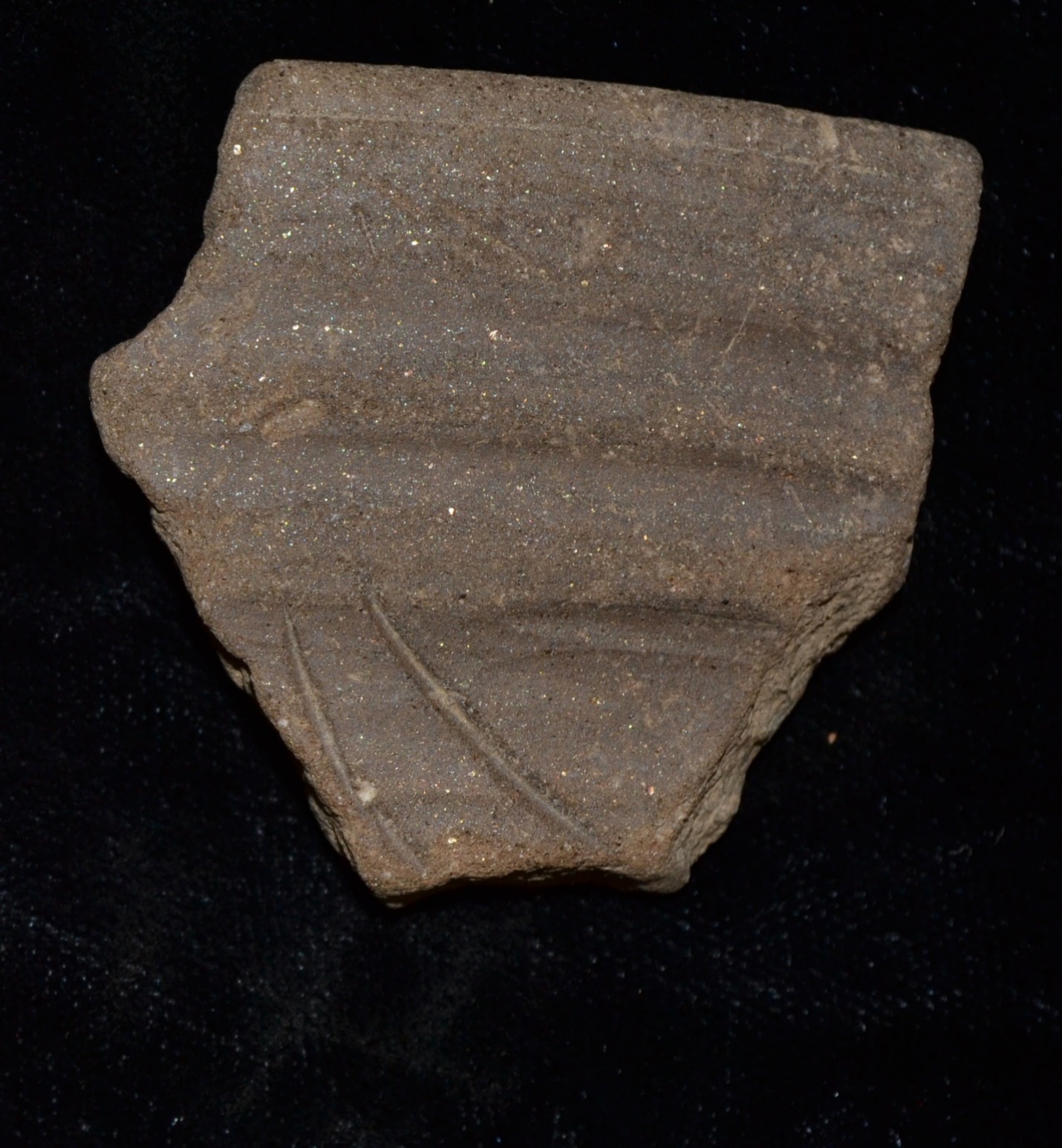


AppendixA.23: BS0023


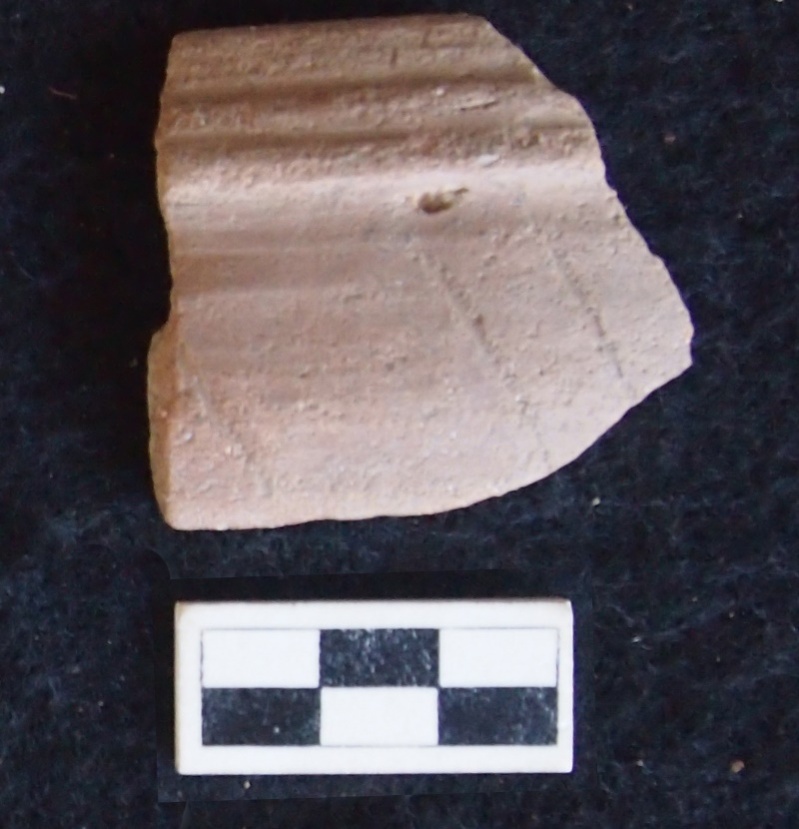


Appendix A.24: BS0024


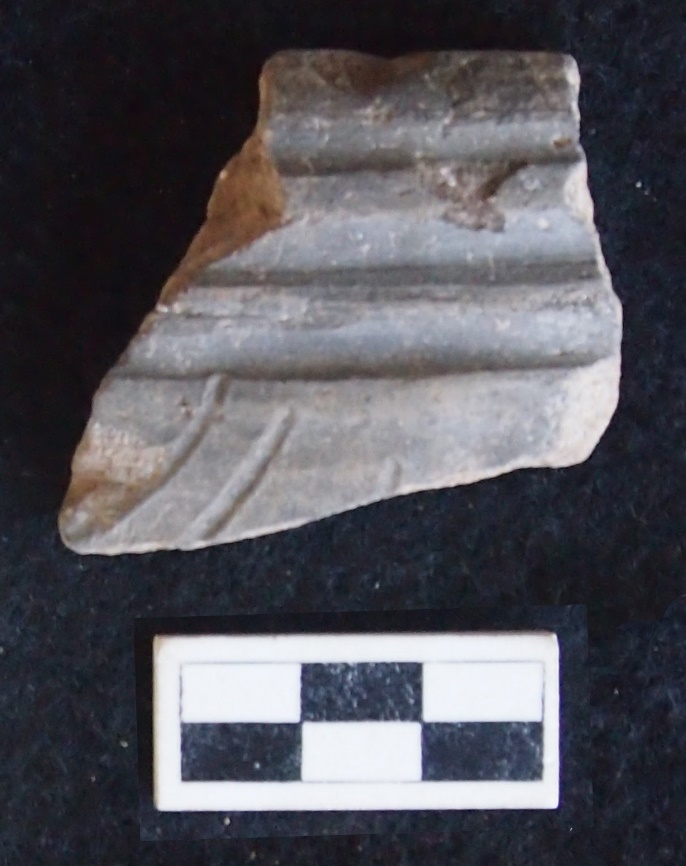


AppendixA.25: BS0025


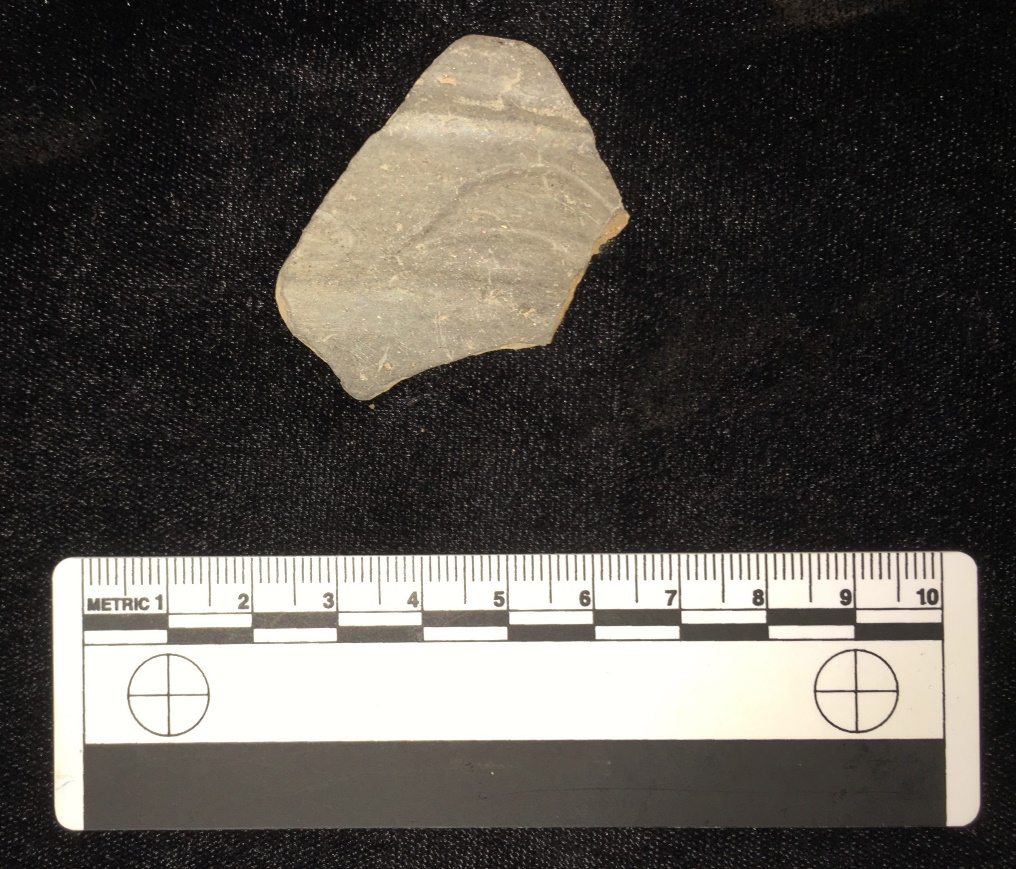


AppendixA.26: BS0027 (see [1], Fig. 2A for image of BS0026)


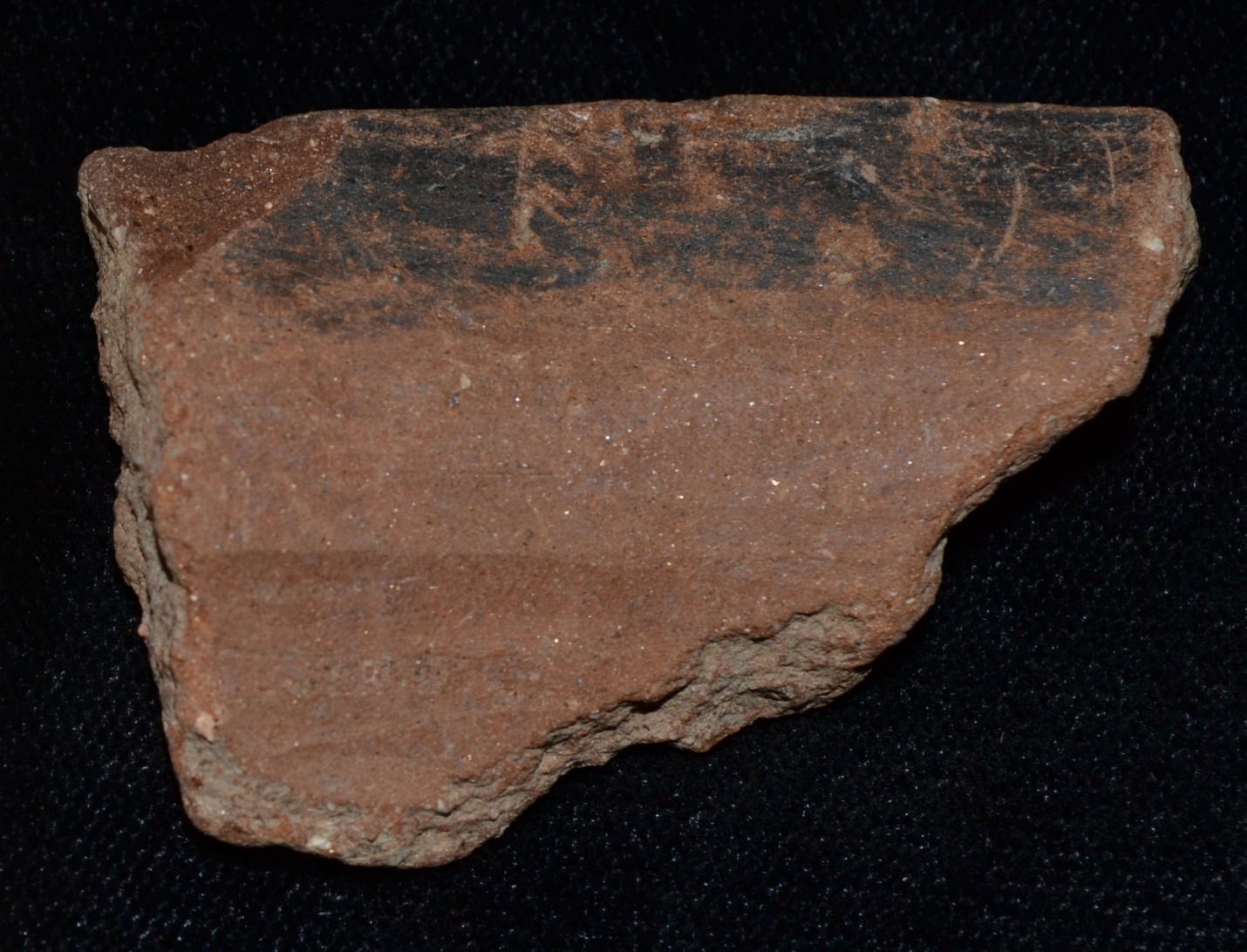


Appendix A.27: BS0028


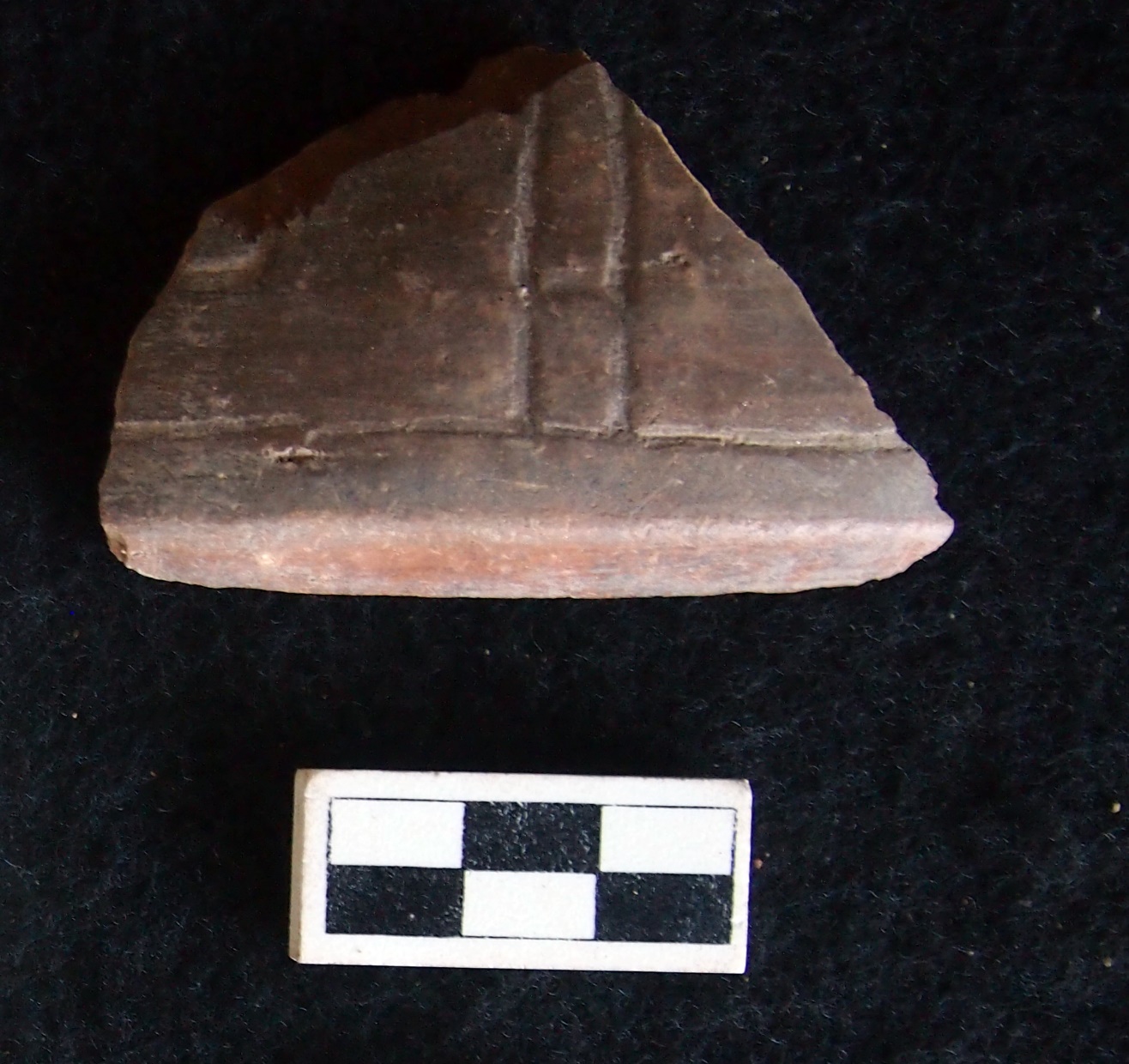


Appendix A.28: BS0029


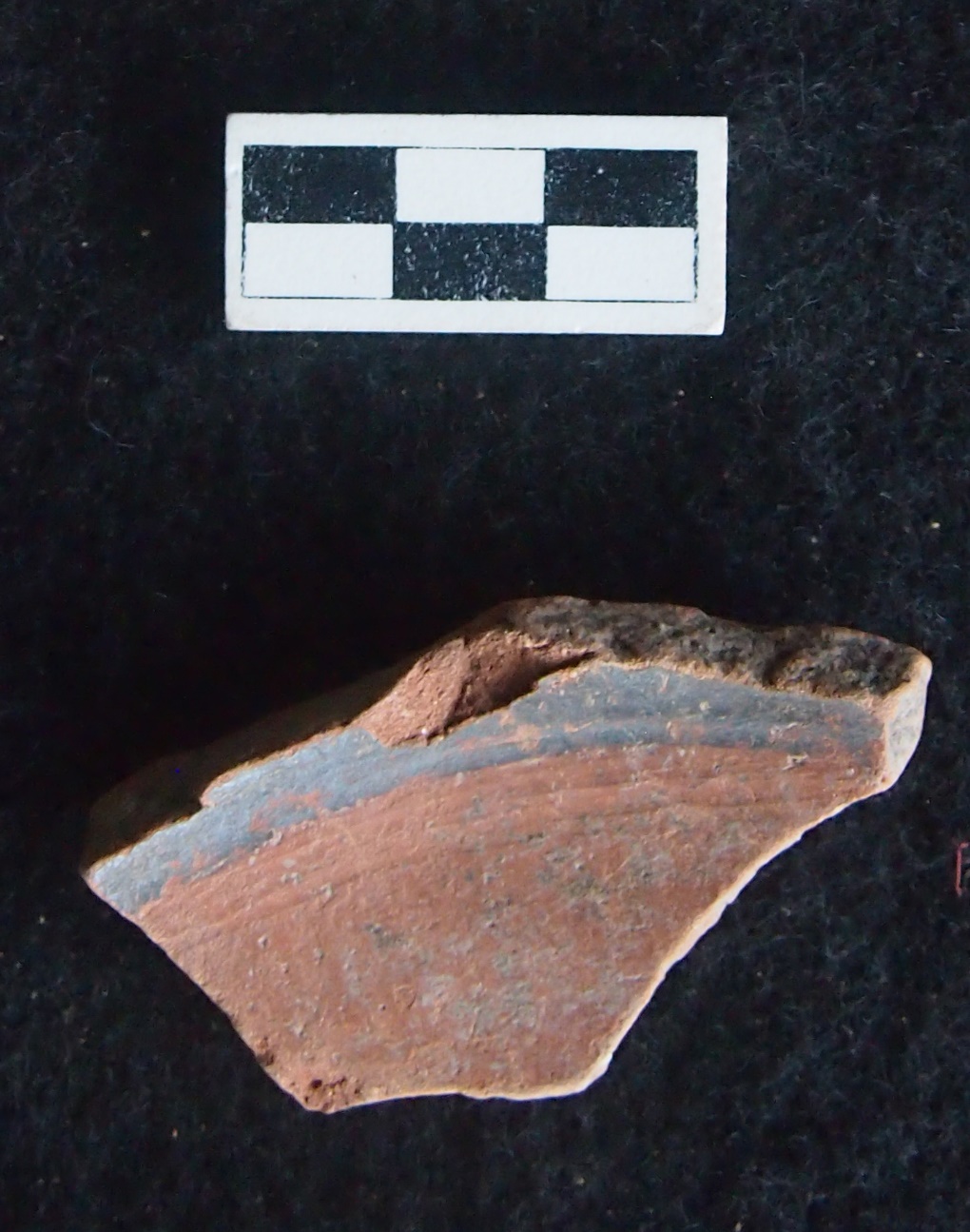


Appendix A.29: BS0030


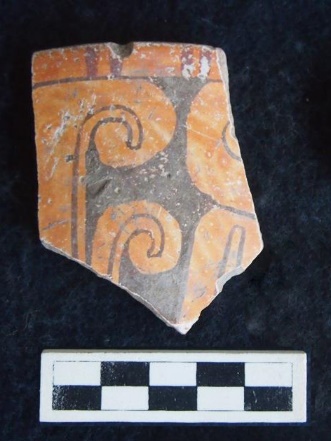


Appendix A.30: BS0031 exterior


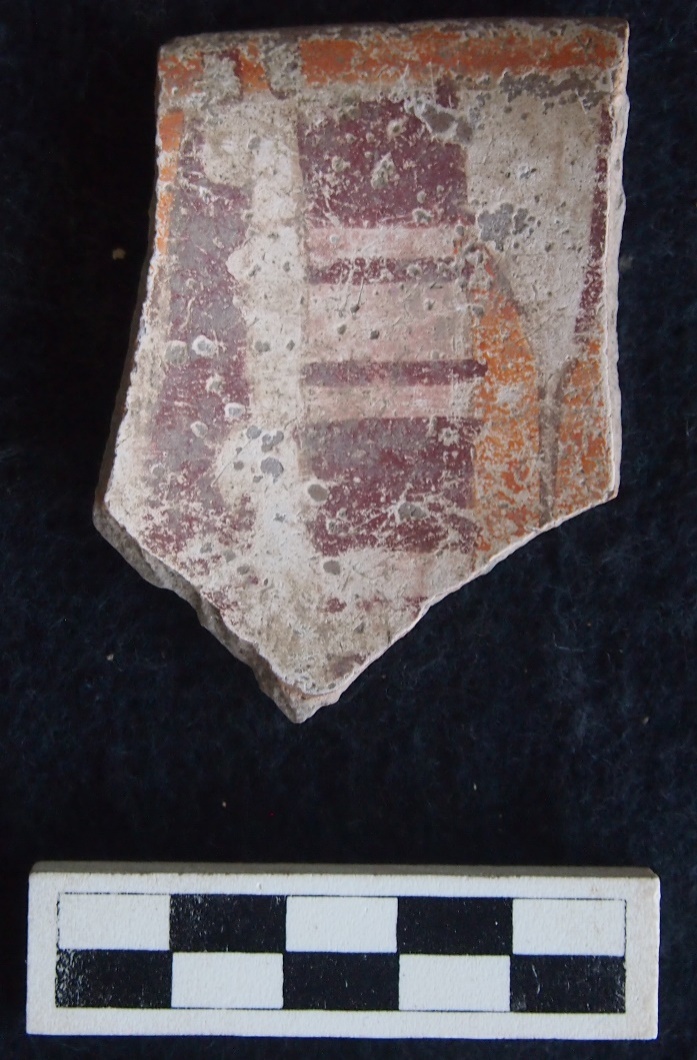


Appendix A.31: BS0031 interior


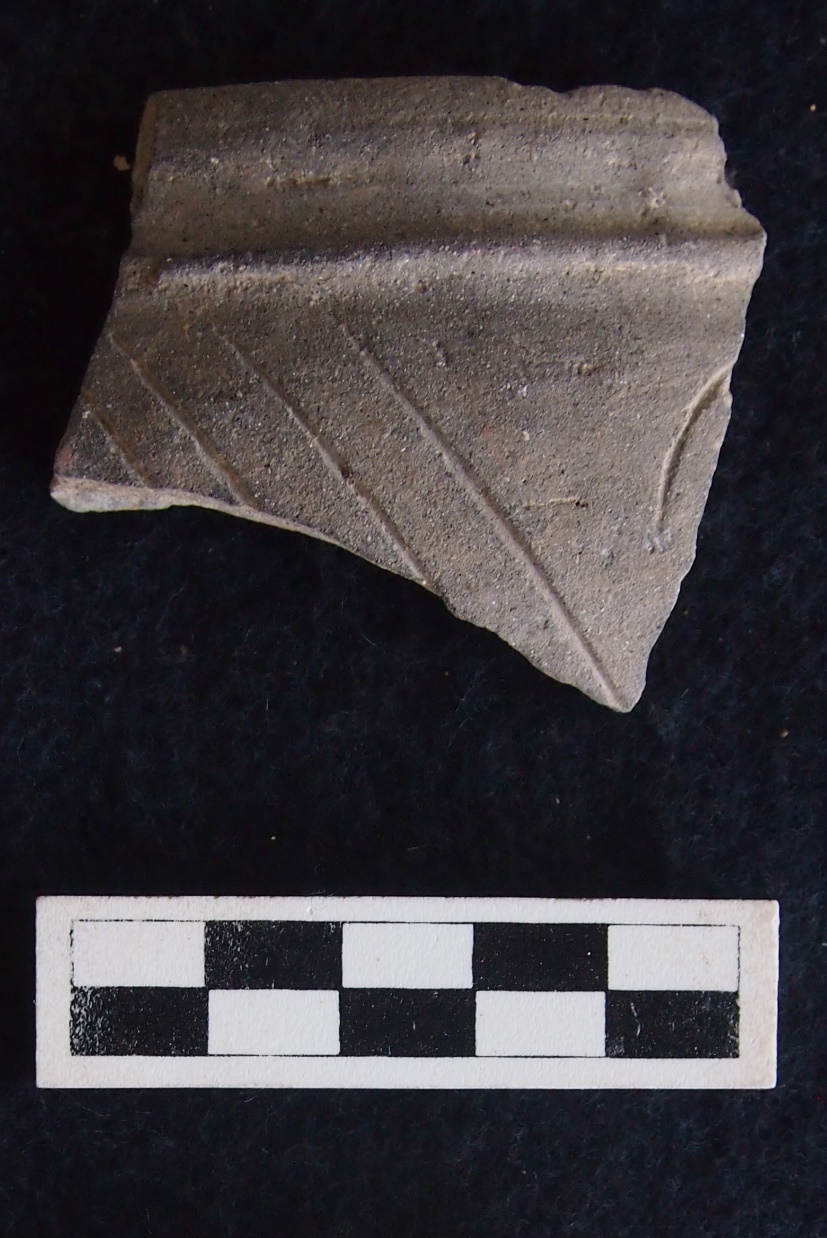


Appendix A.32: BS0032


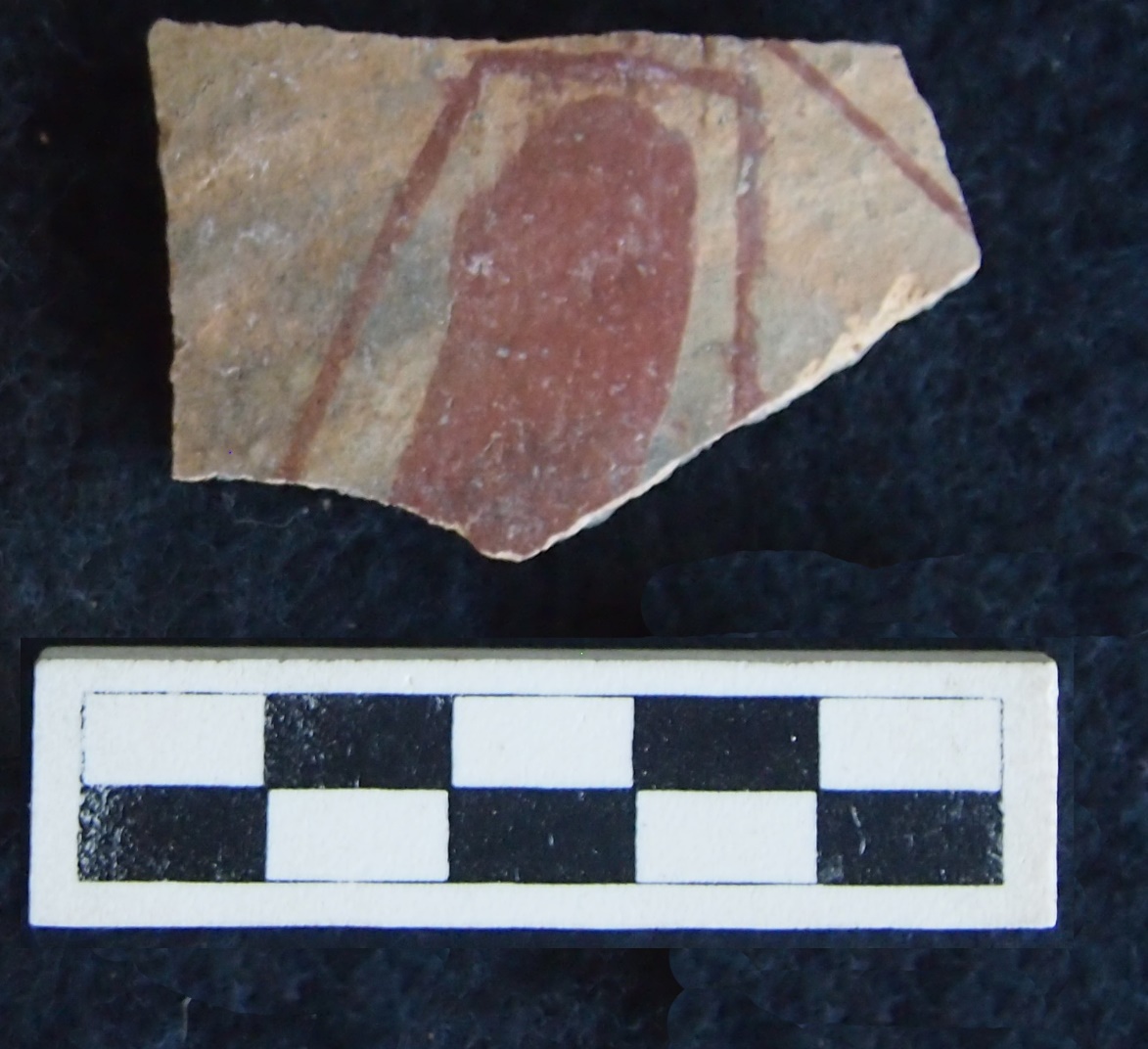


Appendix A.33: BS0033


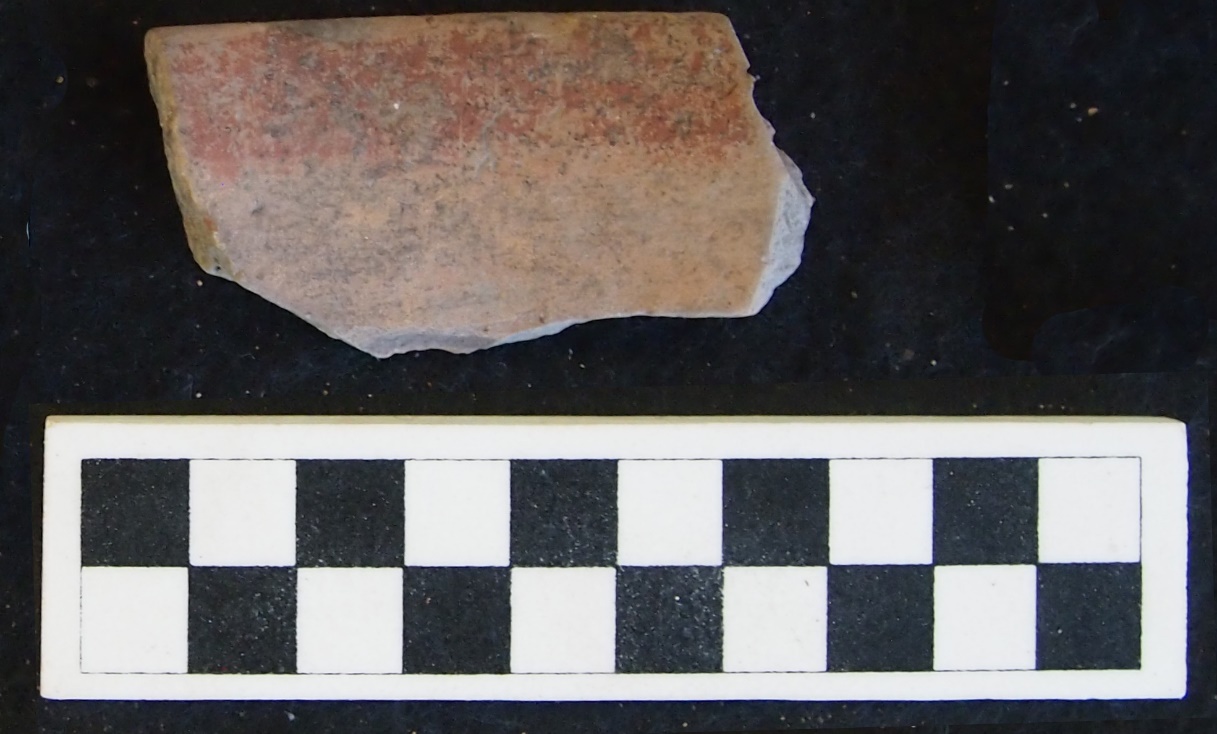


Appendix A.34: BS0034


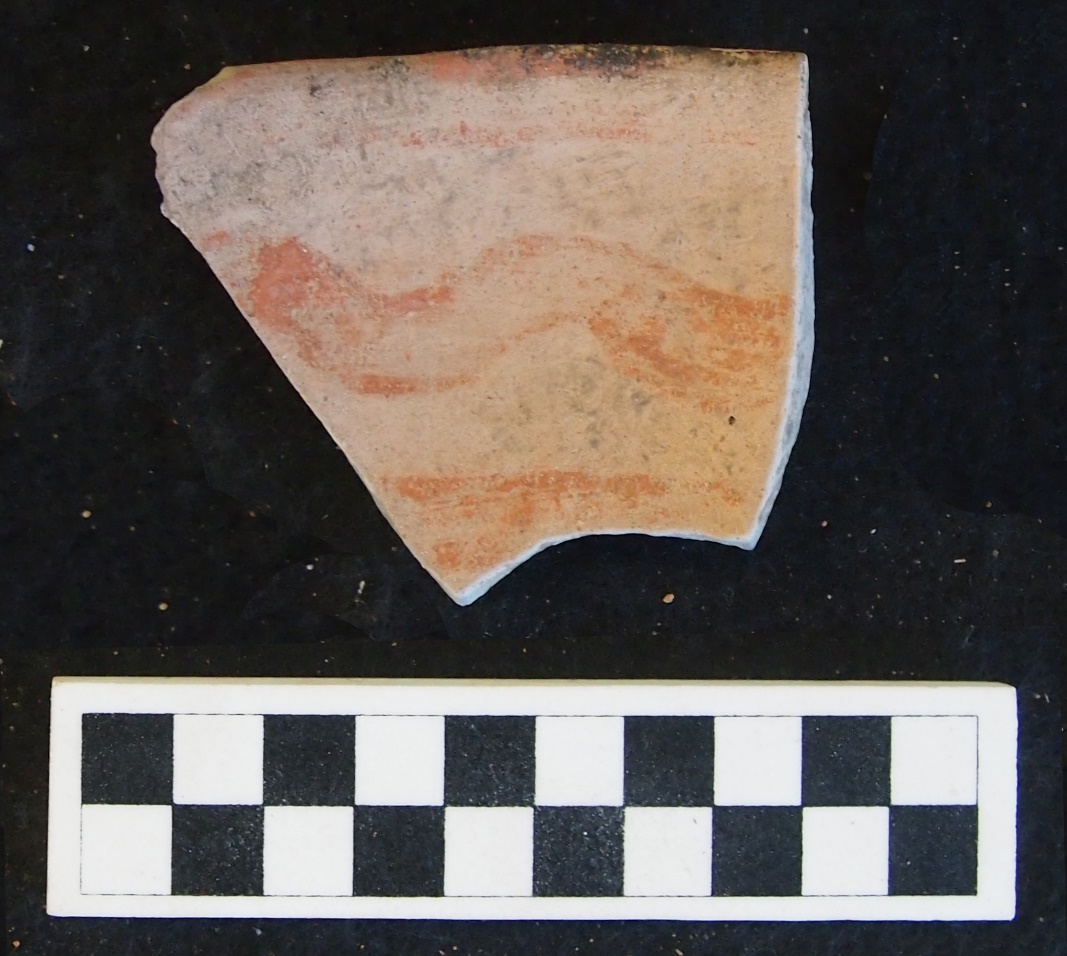


Appendix A.35: BS0035


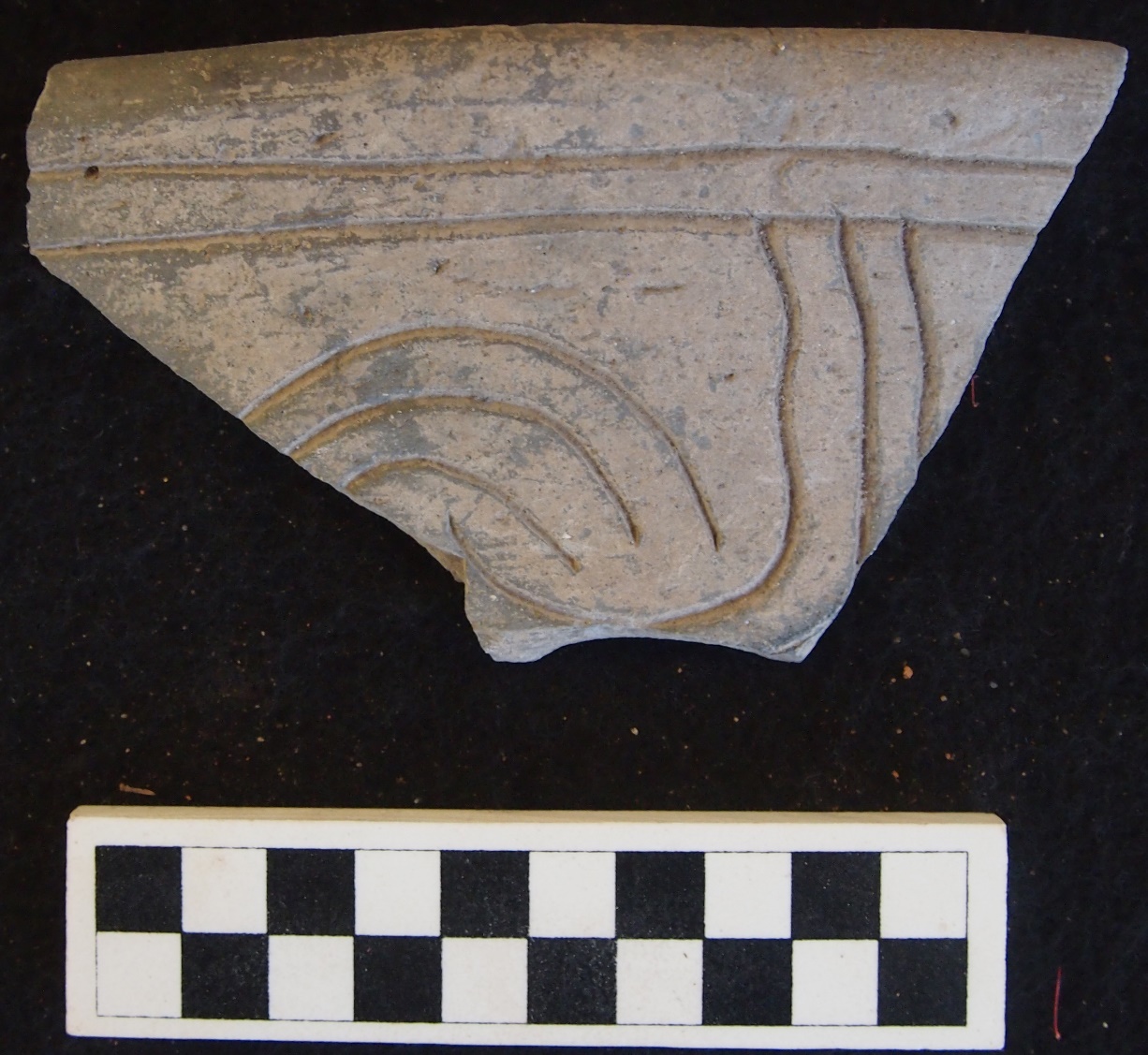


Appendix A.36: BS0036


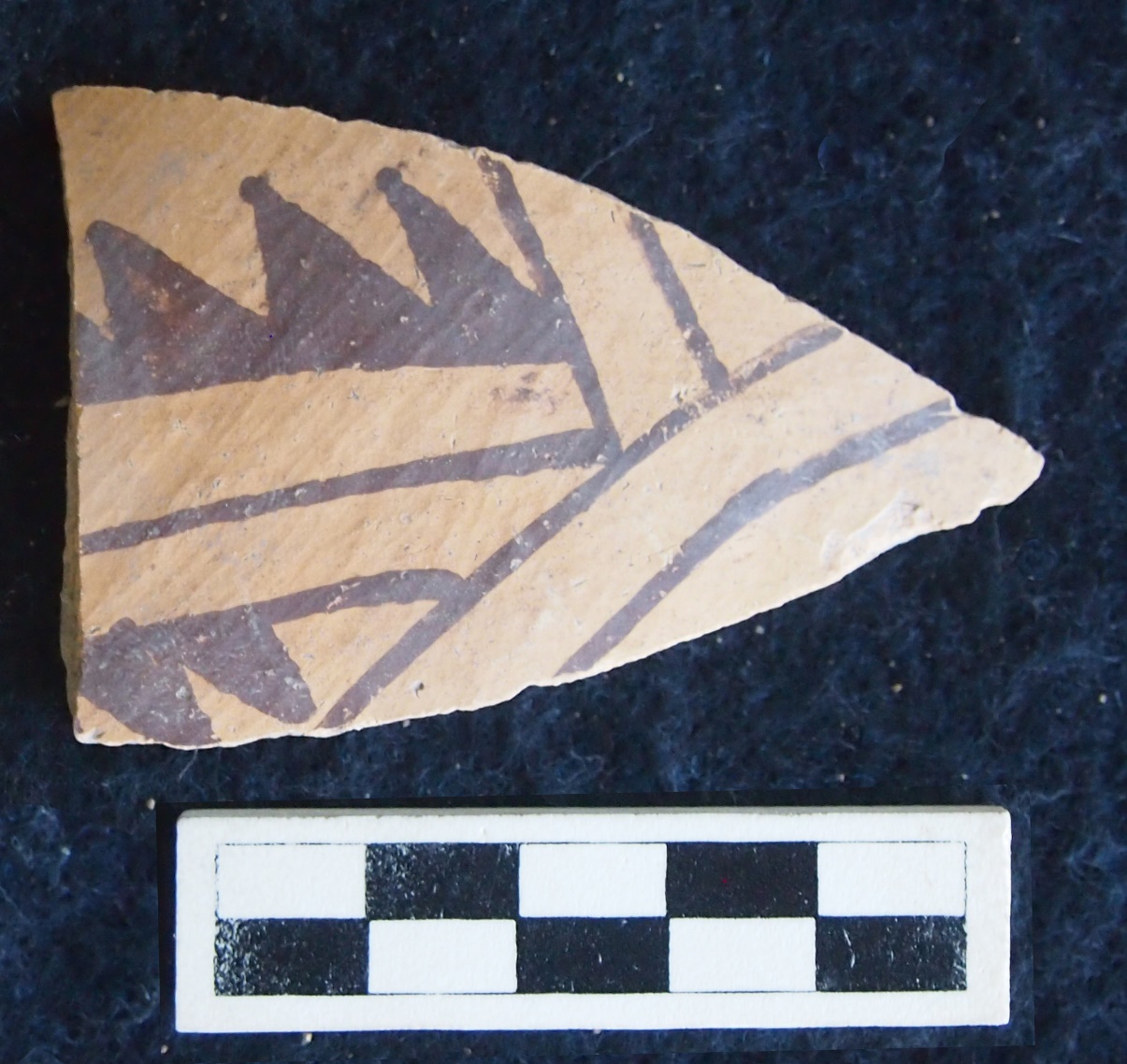


Appendix A.37: BS0037


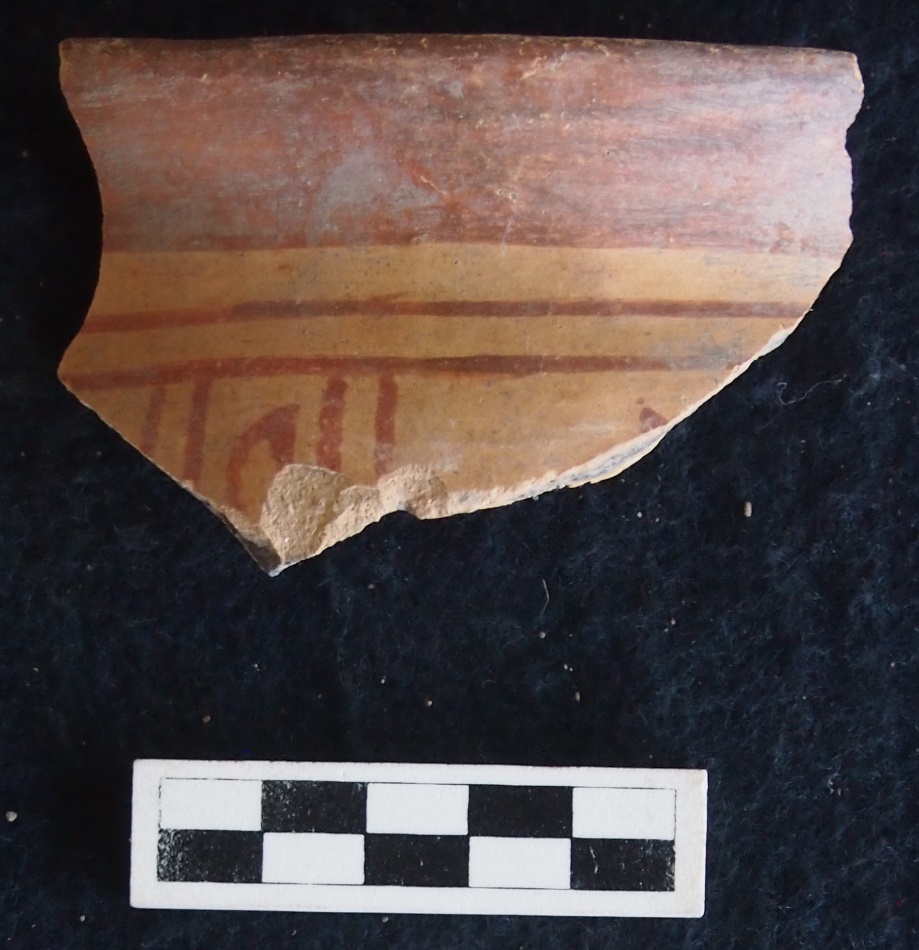


Appendix A.38: BS0038


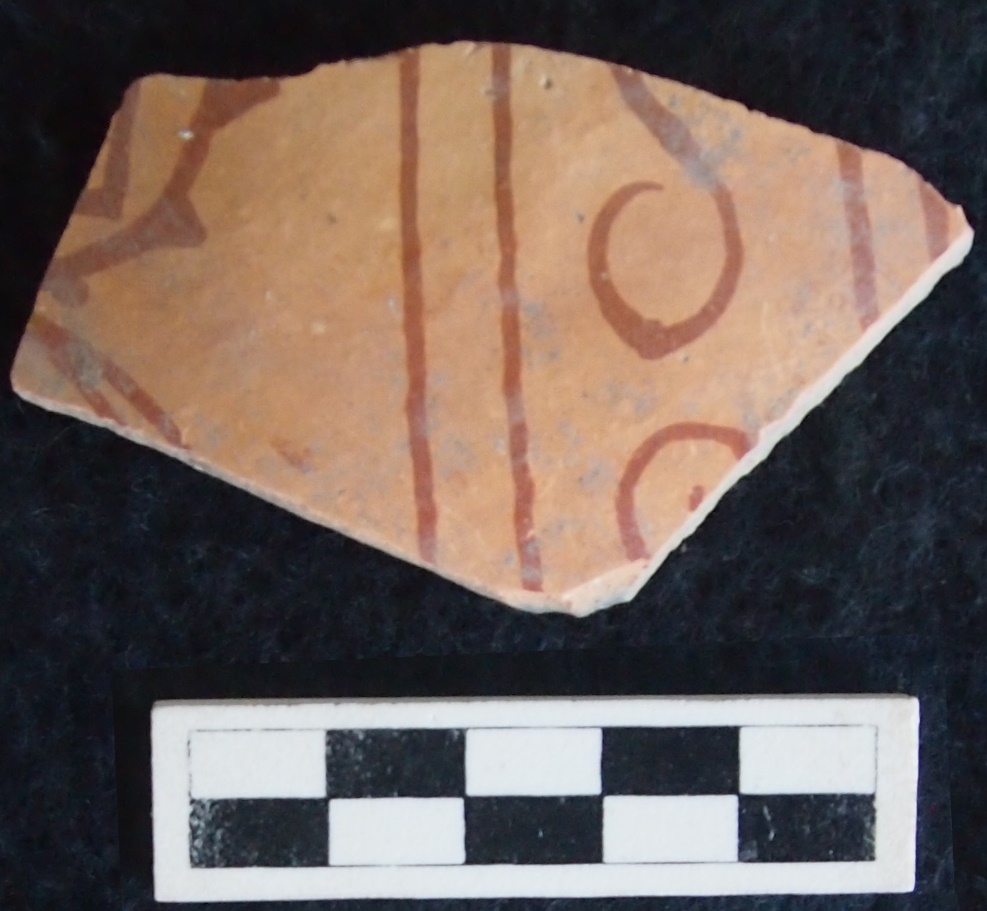


Appendix A.39: BS0039


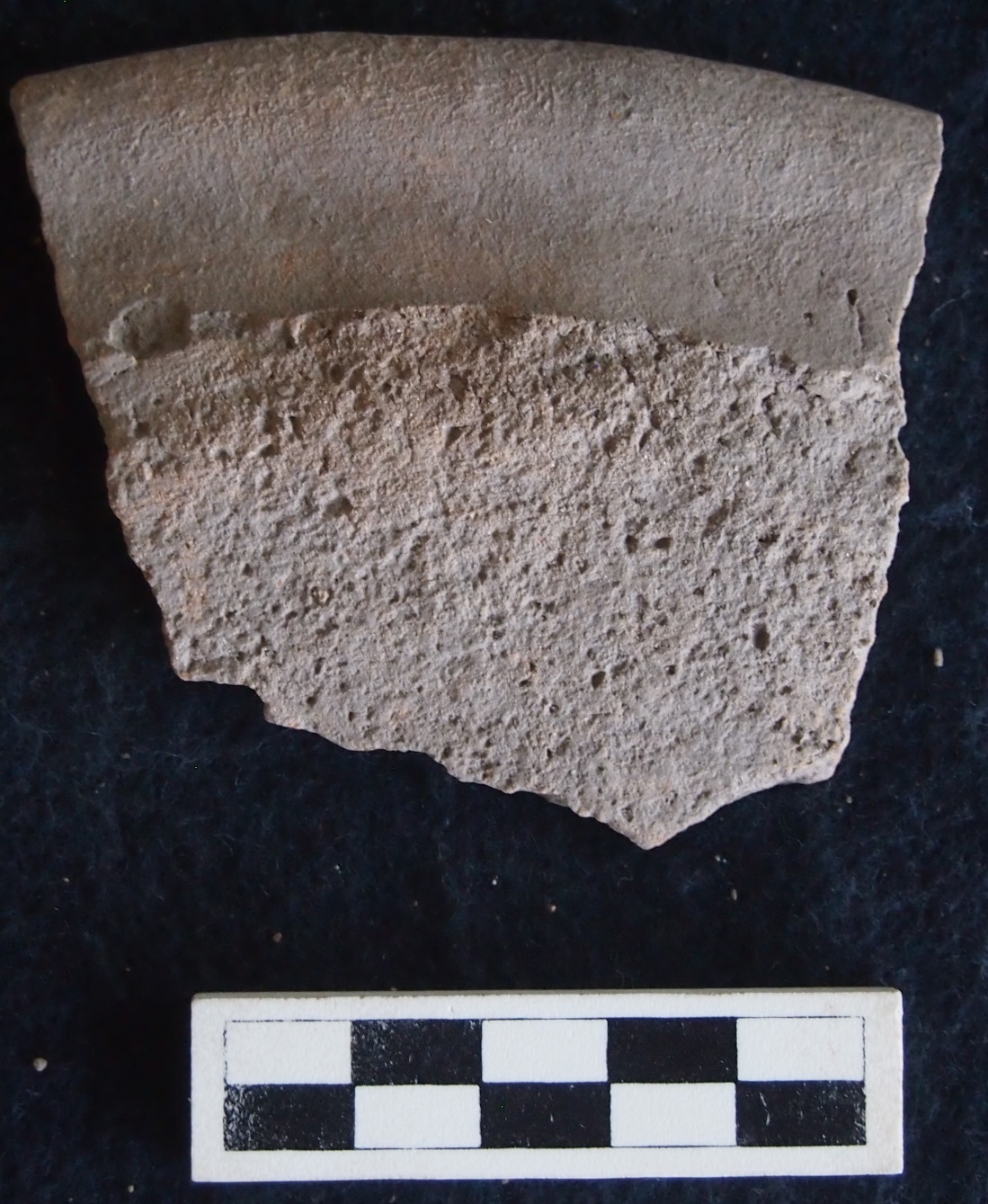


Appendix A.40: BS0040 exterior


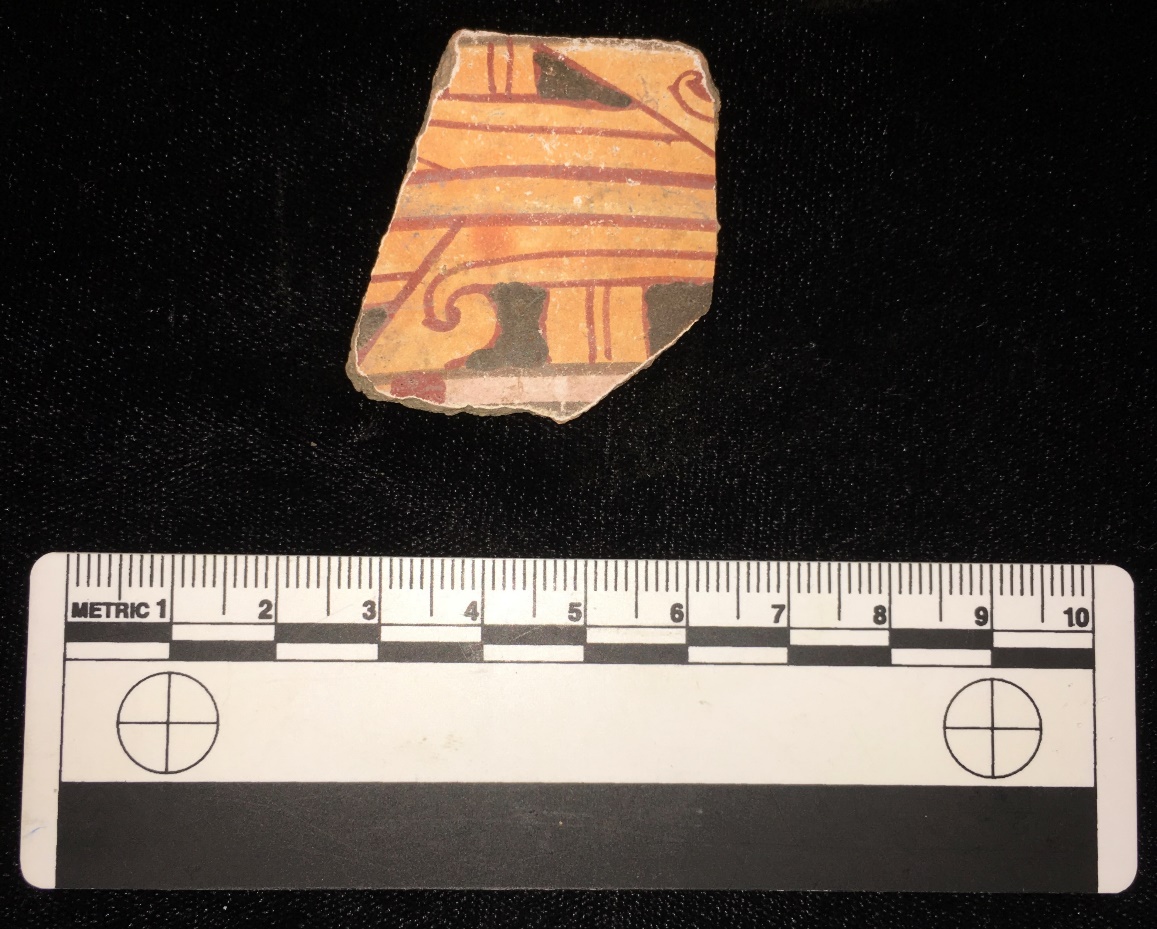


Appendix A.41: BS0041


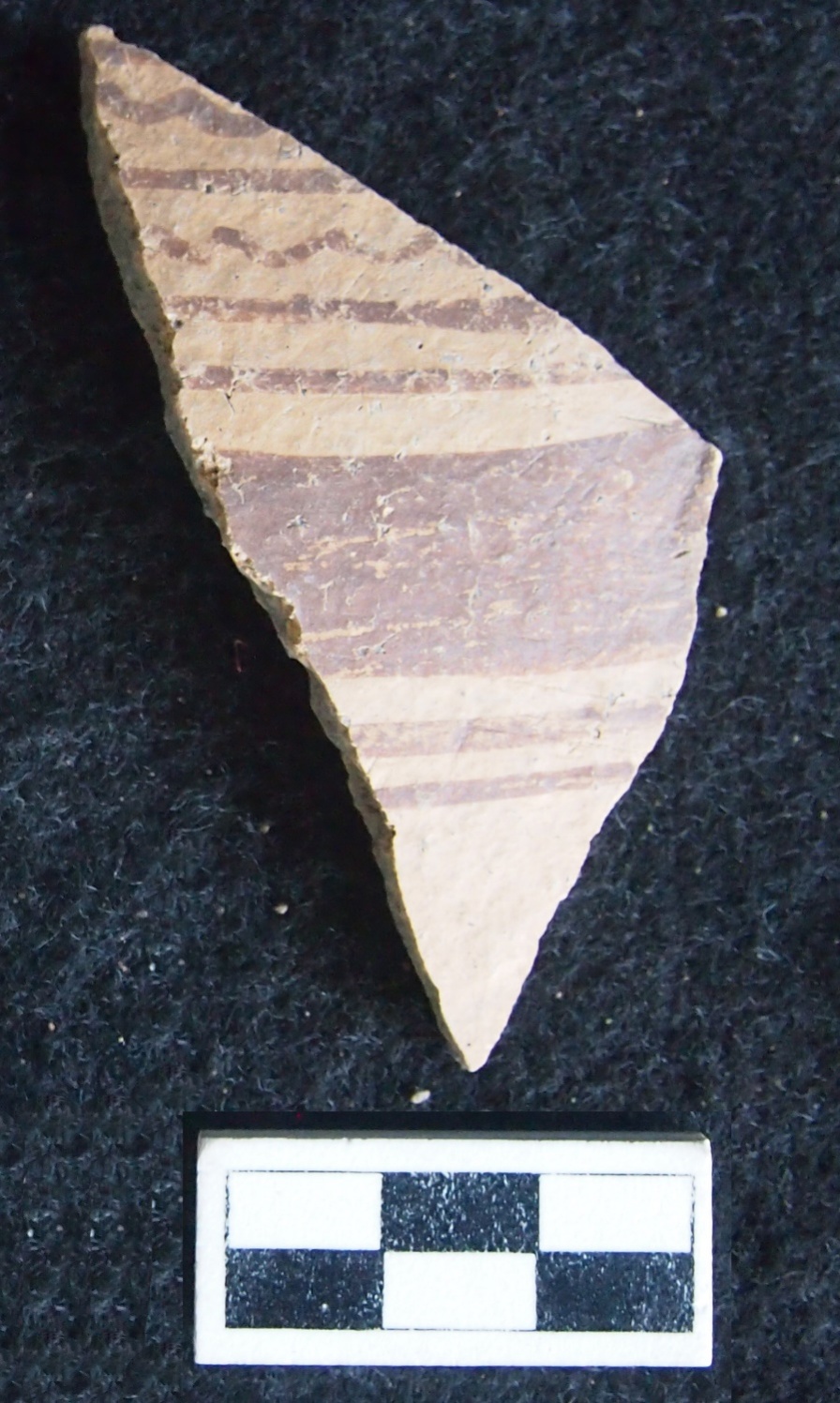


Appendix A.42: BS0042


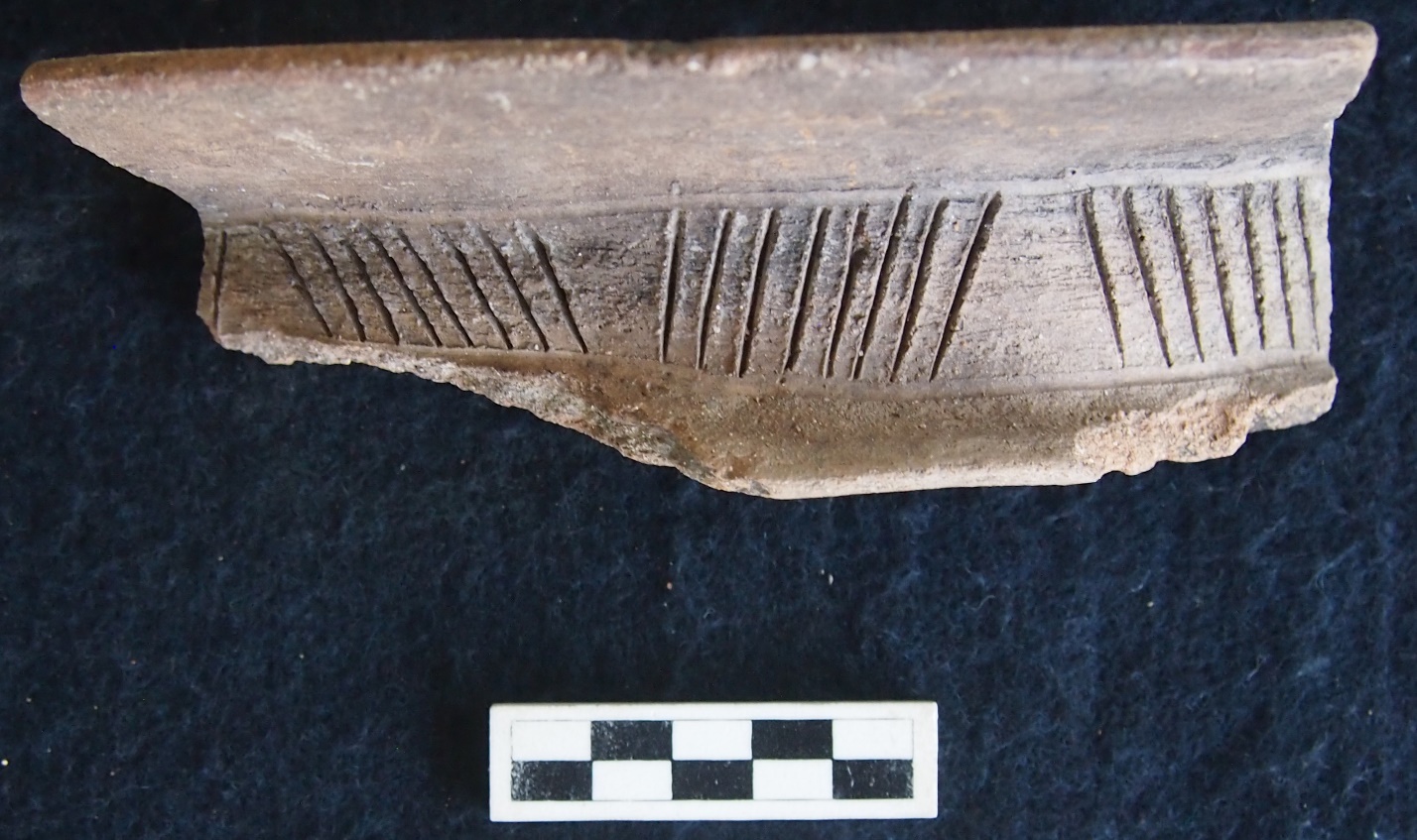


Appendix A.43: BS0046 (see [1], Fig. 4B for image of BS0043, Fig. 2B for image of BS0044, and Fig. 6A for image of BS0045)


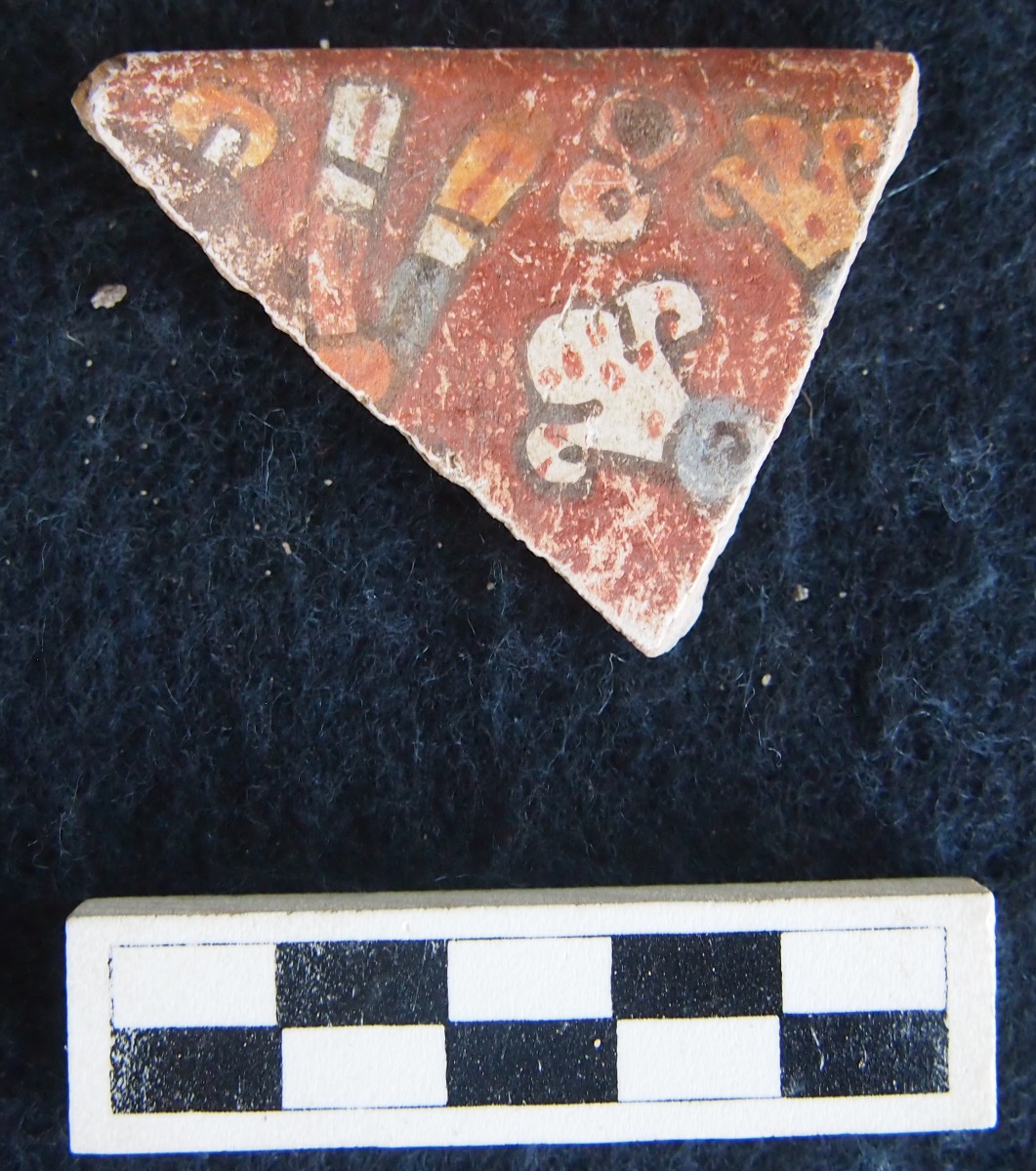


Appendix A.44: BS0047 interior


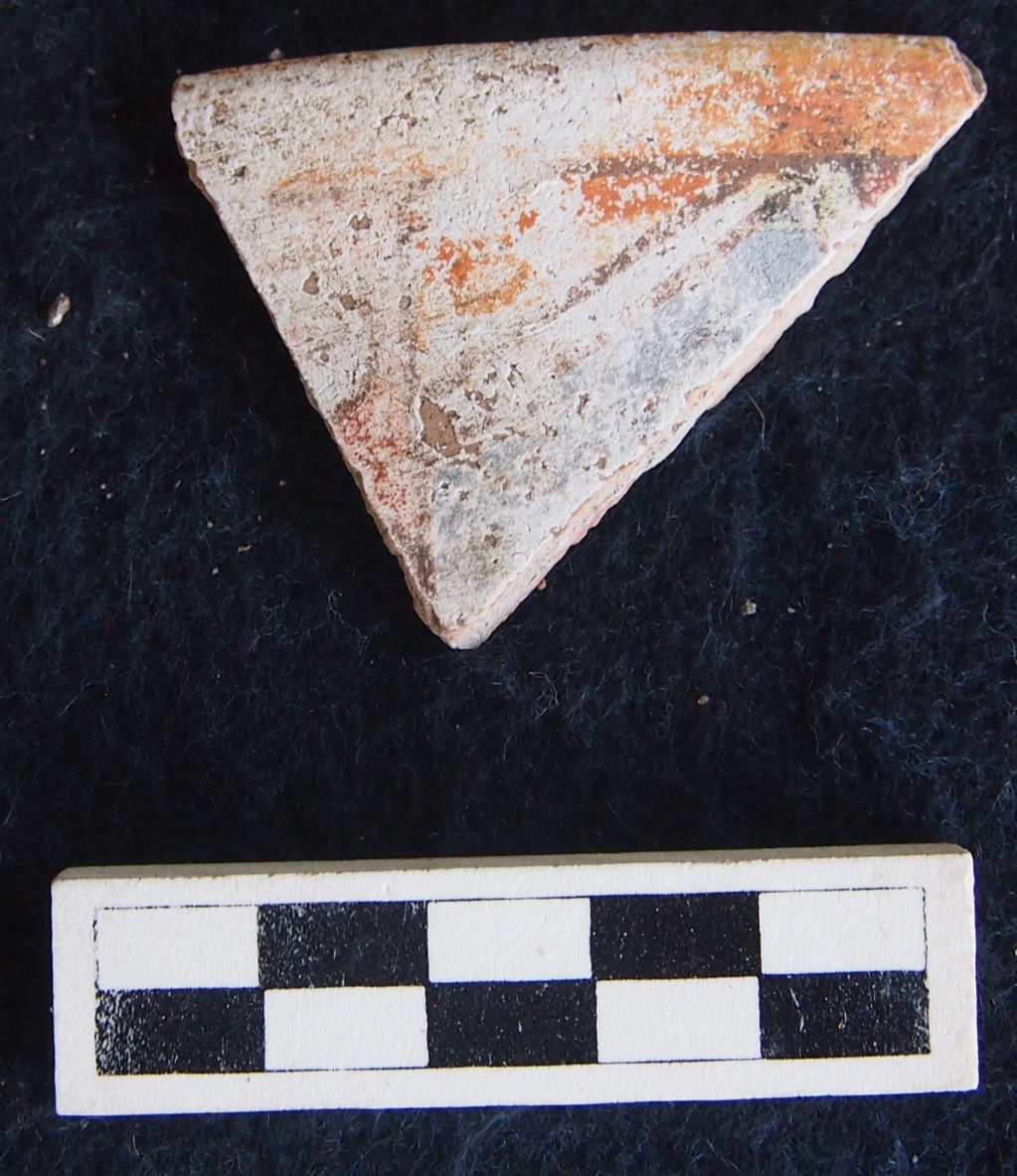


Appendix A.45: BS0047 exterior


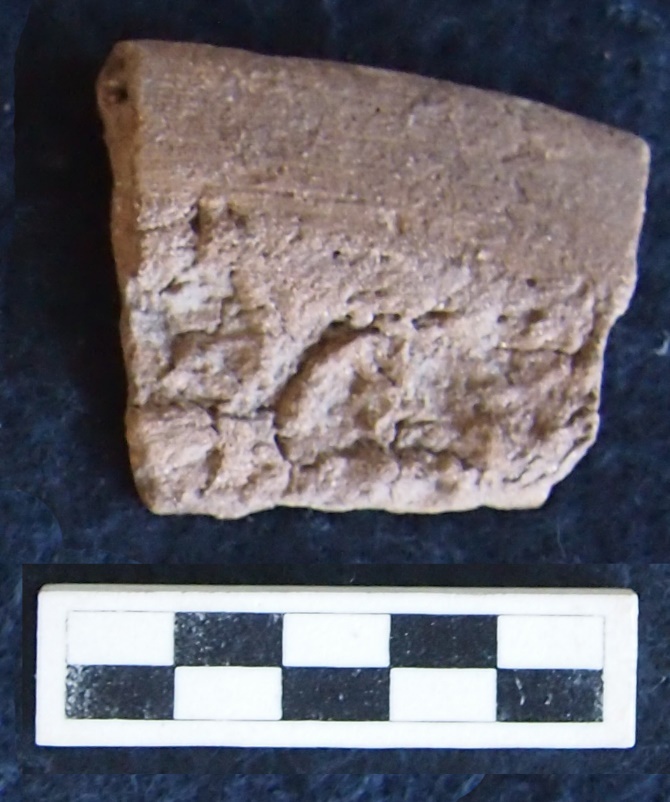


Appendix A.46: BS0048


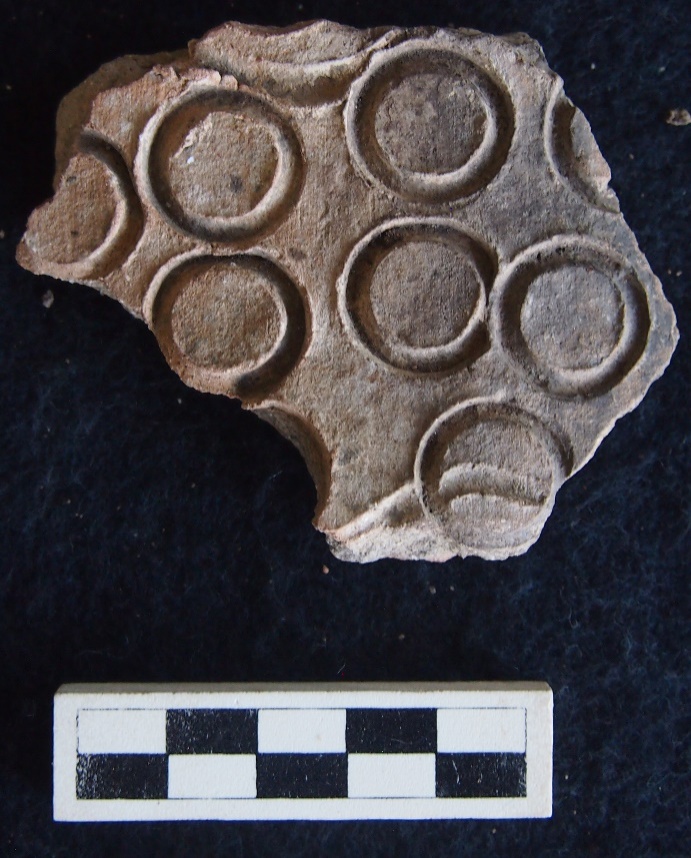


Appendix A.47: BS0049


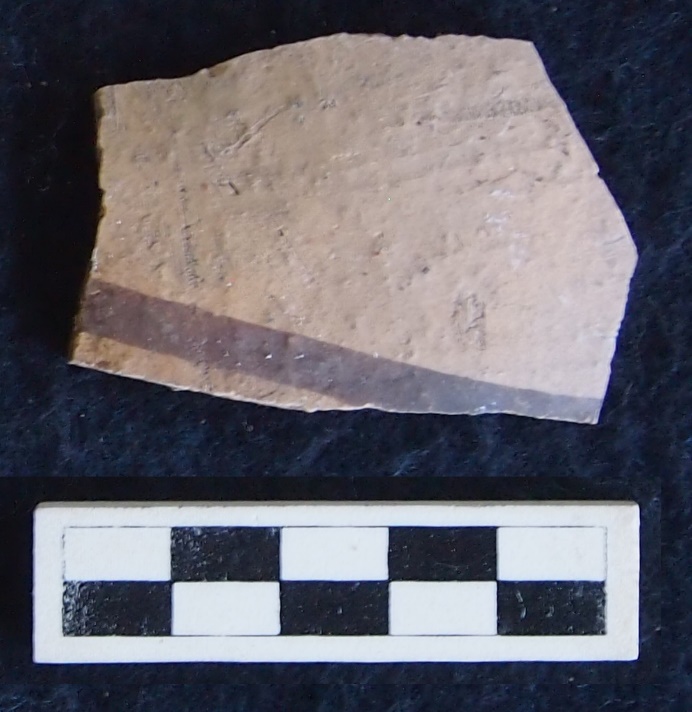


Appendix A.48: BS0050


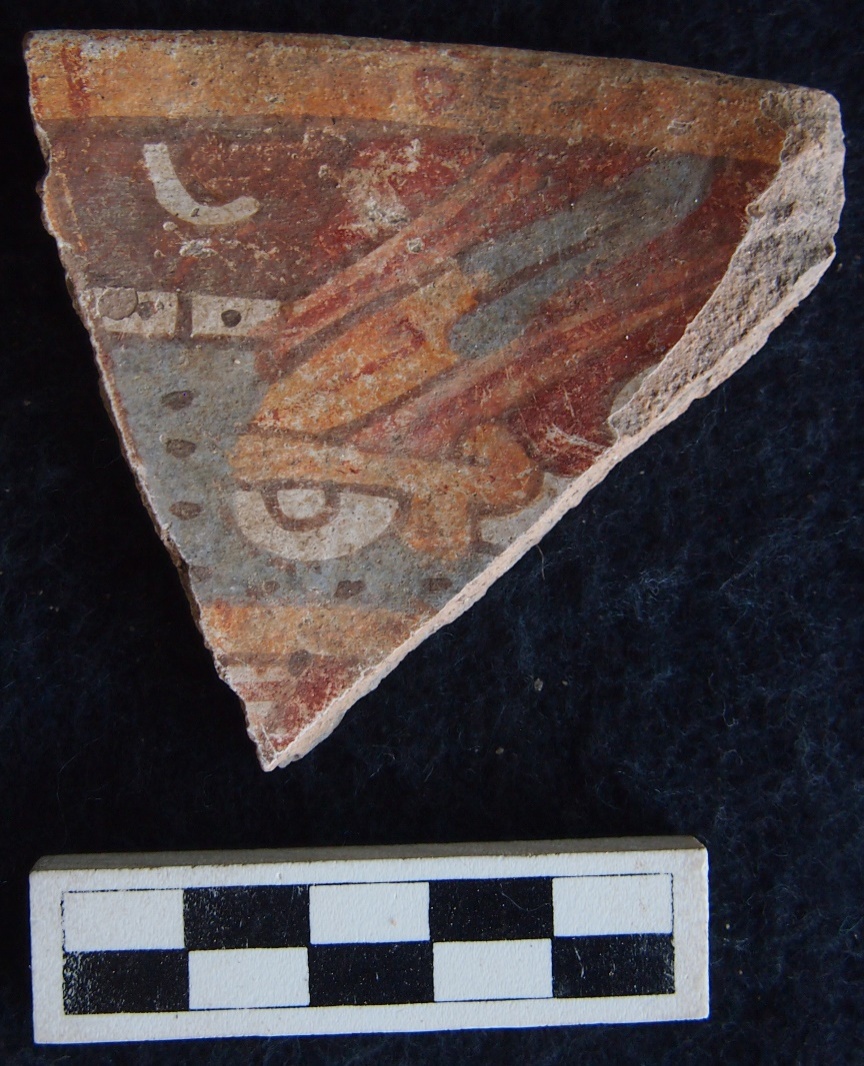


Appendix A.49: BS0051interior


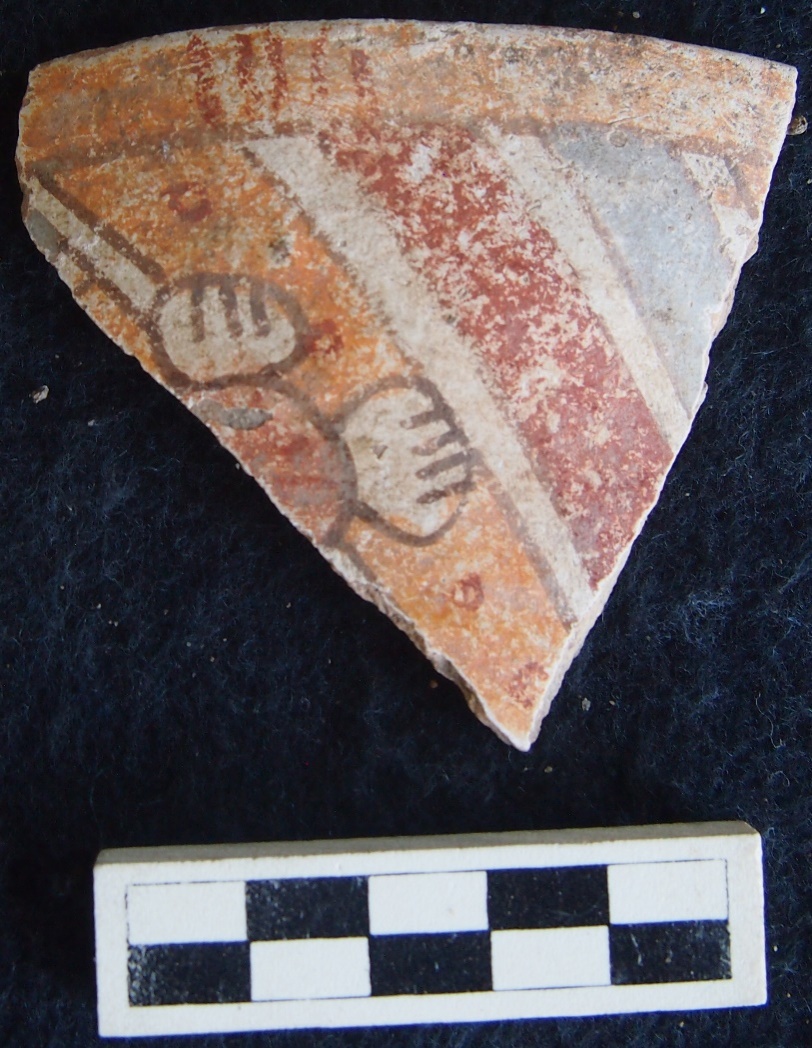


Appendix A.50: BS0051 exterior


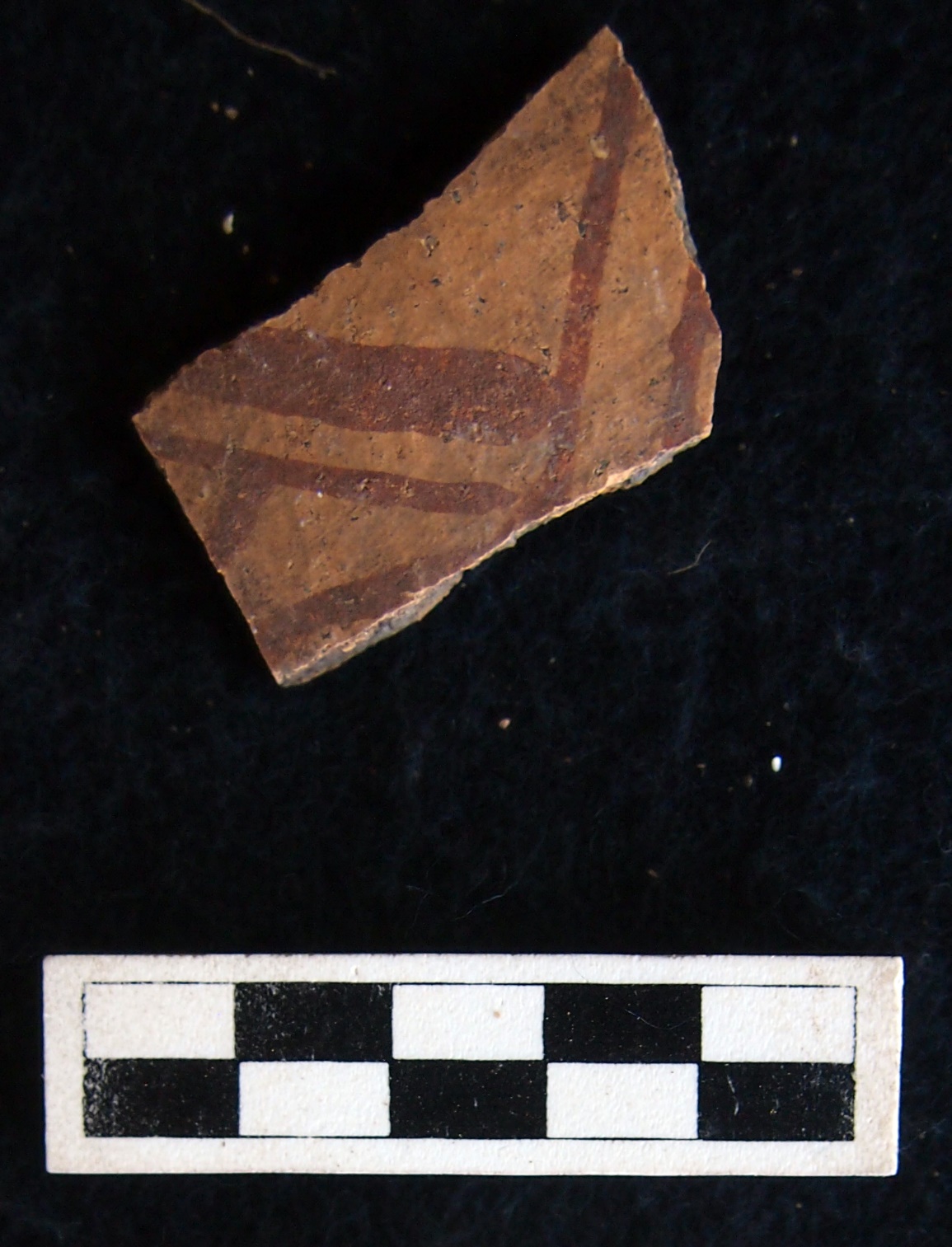


Appendix A.51: BS0052


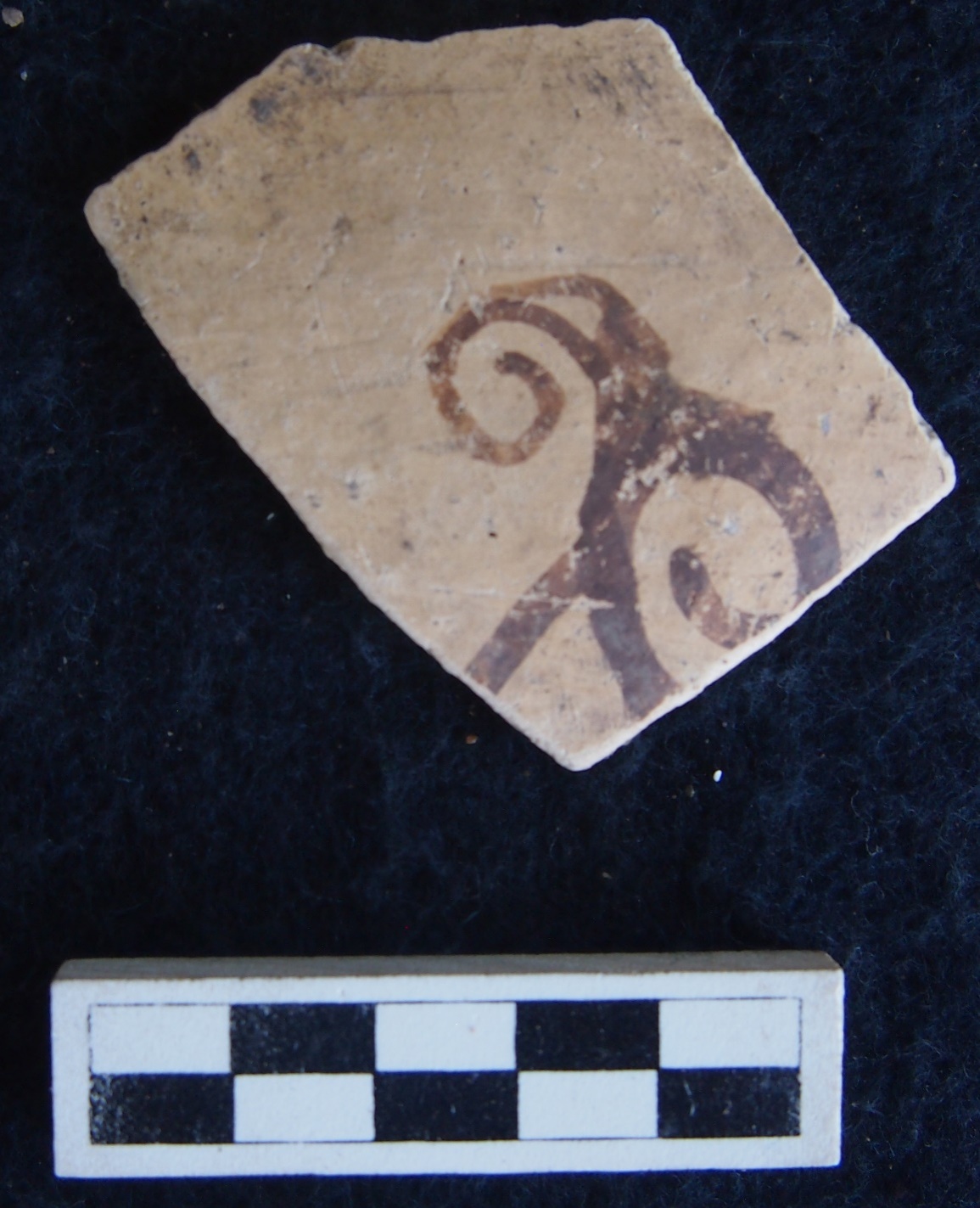


Appendix A.52: BS0053


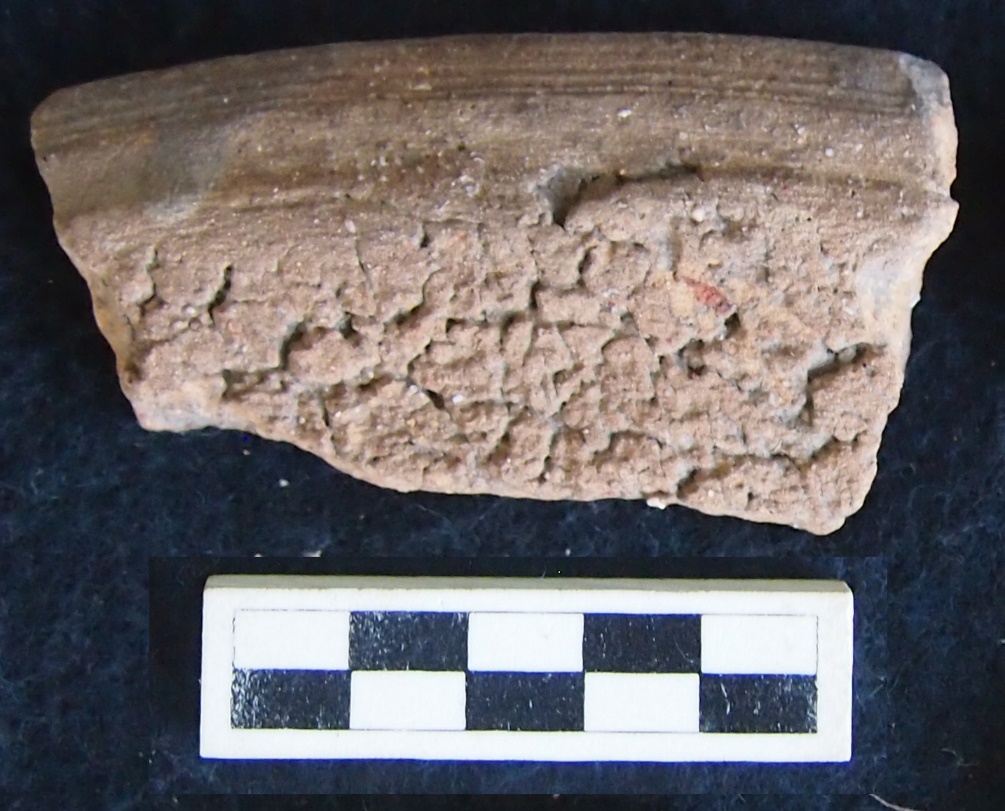


Appendix A.53: BS0054 exterior


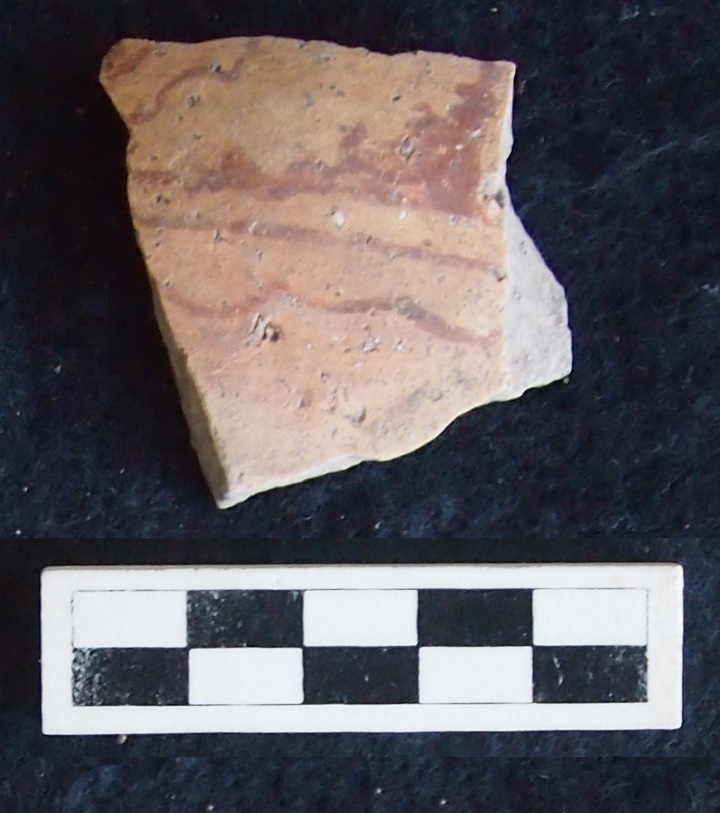


Appendix A.54: BS0055


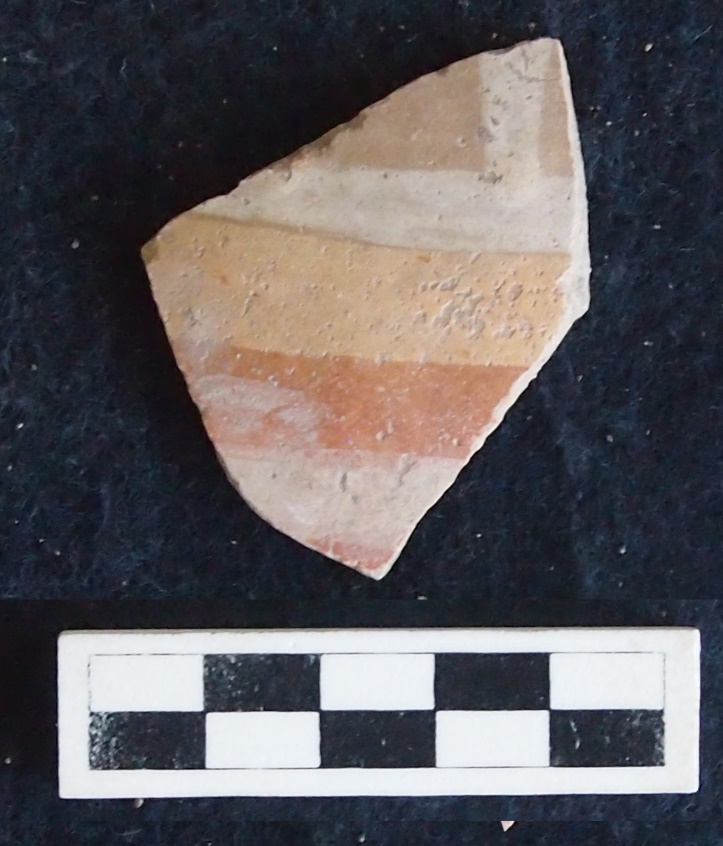


Appendix A.55: BS0056


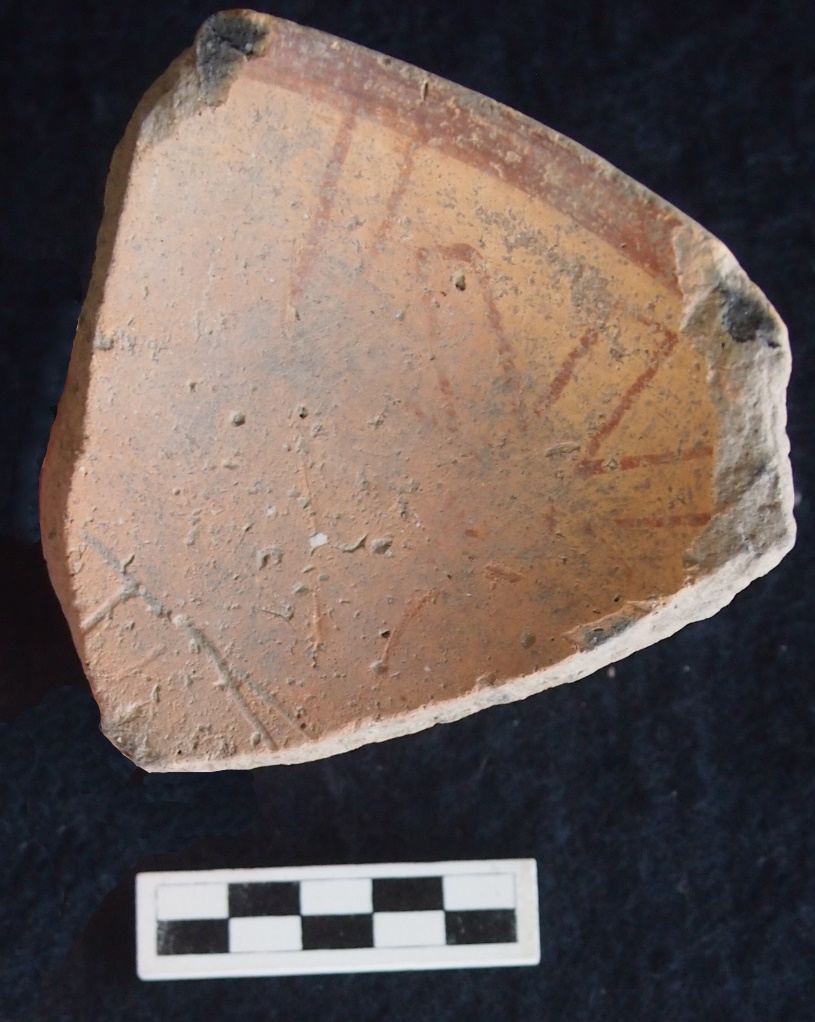


Appendix A.56: BS0057


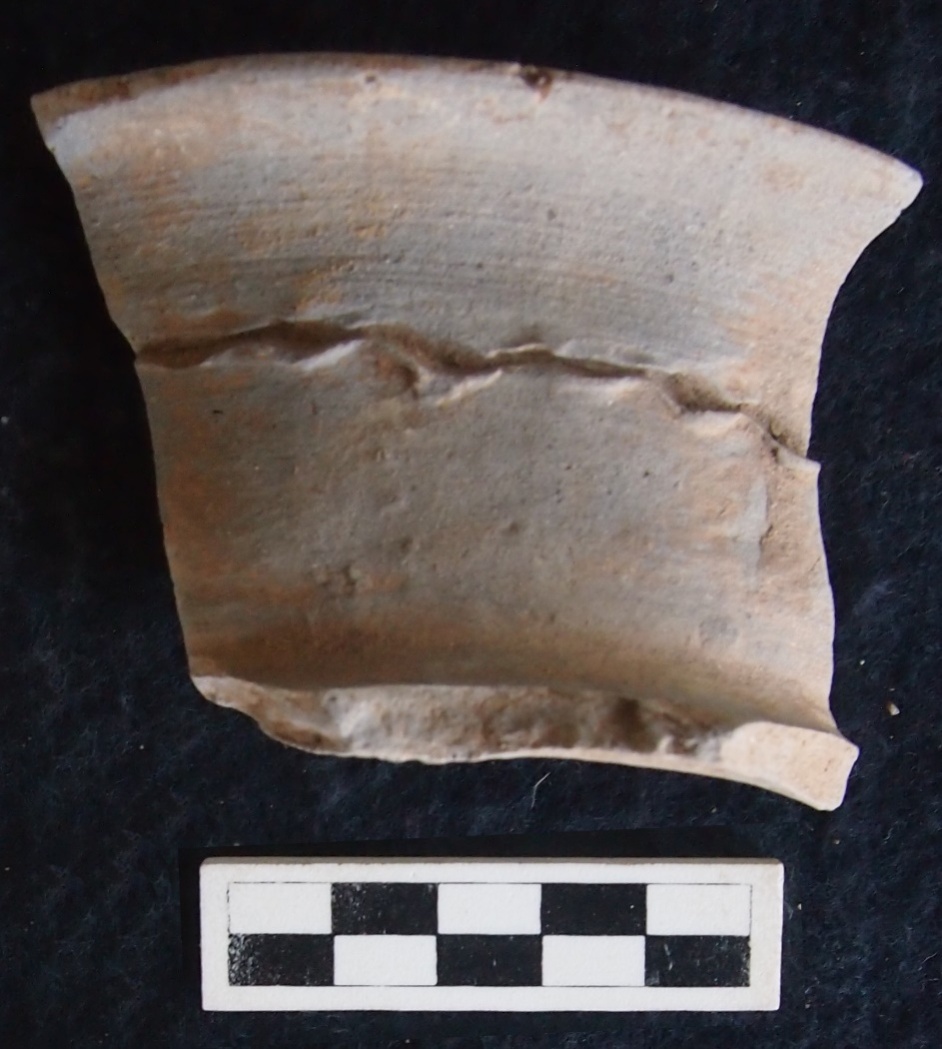


Appendix A.57: BS0060 (see [1], Fig. 2C for image of BS0048 and, Fig. 4A for image ofBS0059)


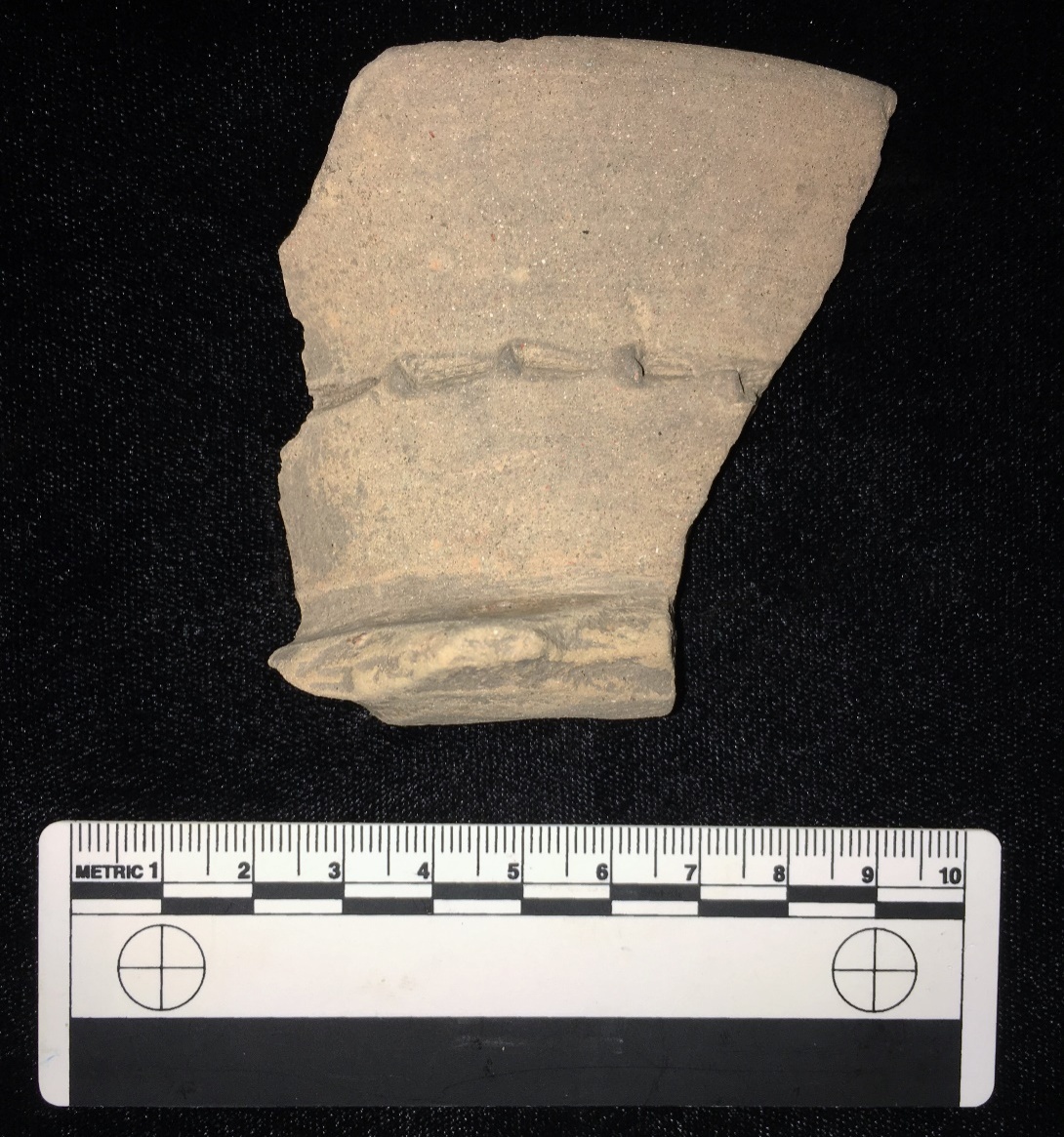


Appendix A.58: BS0061


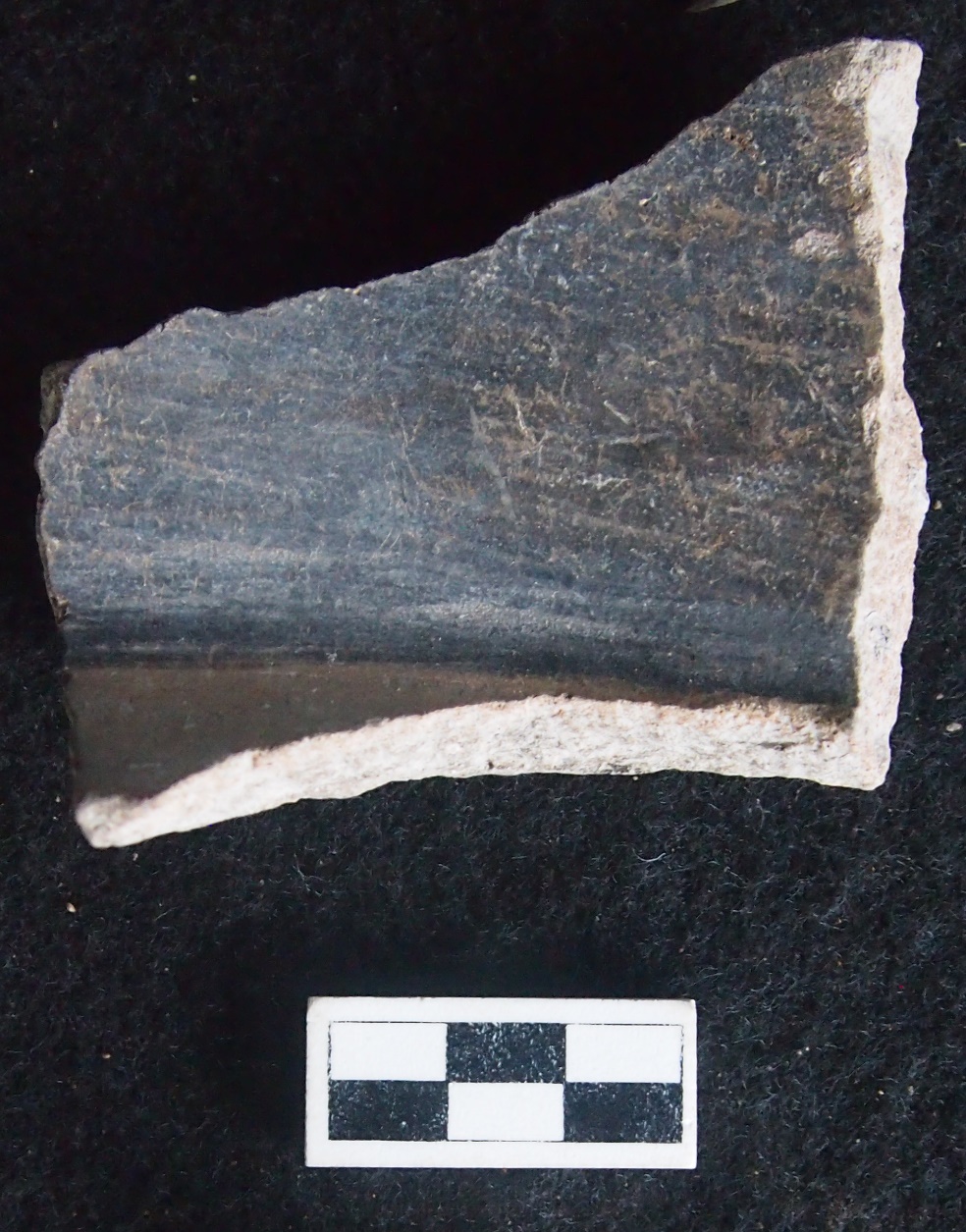


Appendix A.59: BS0062


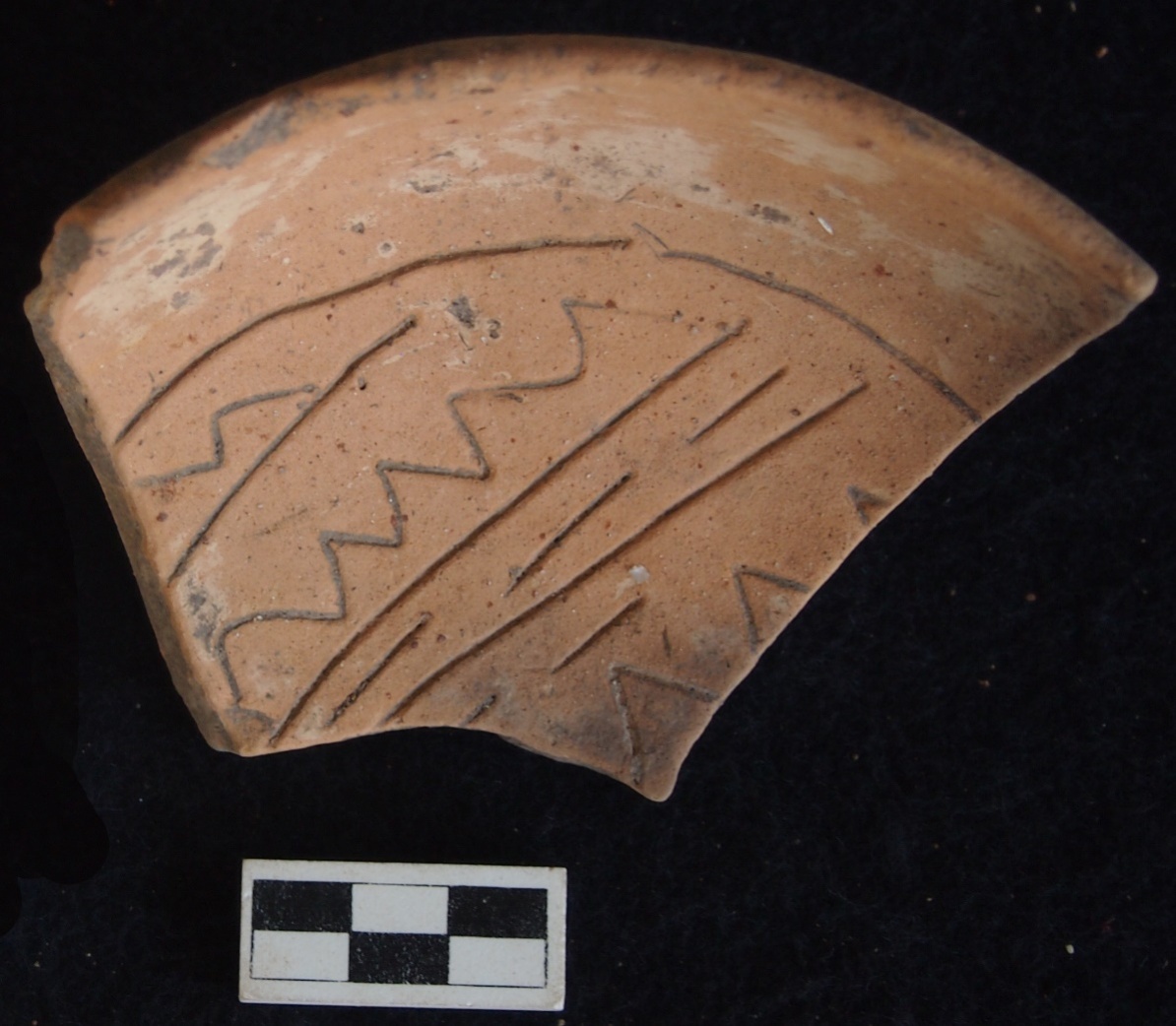


Appendix A.60 BS0064 (BS0063 number not used)


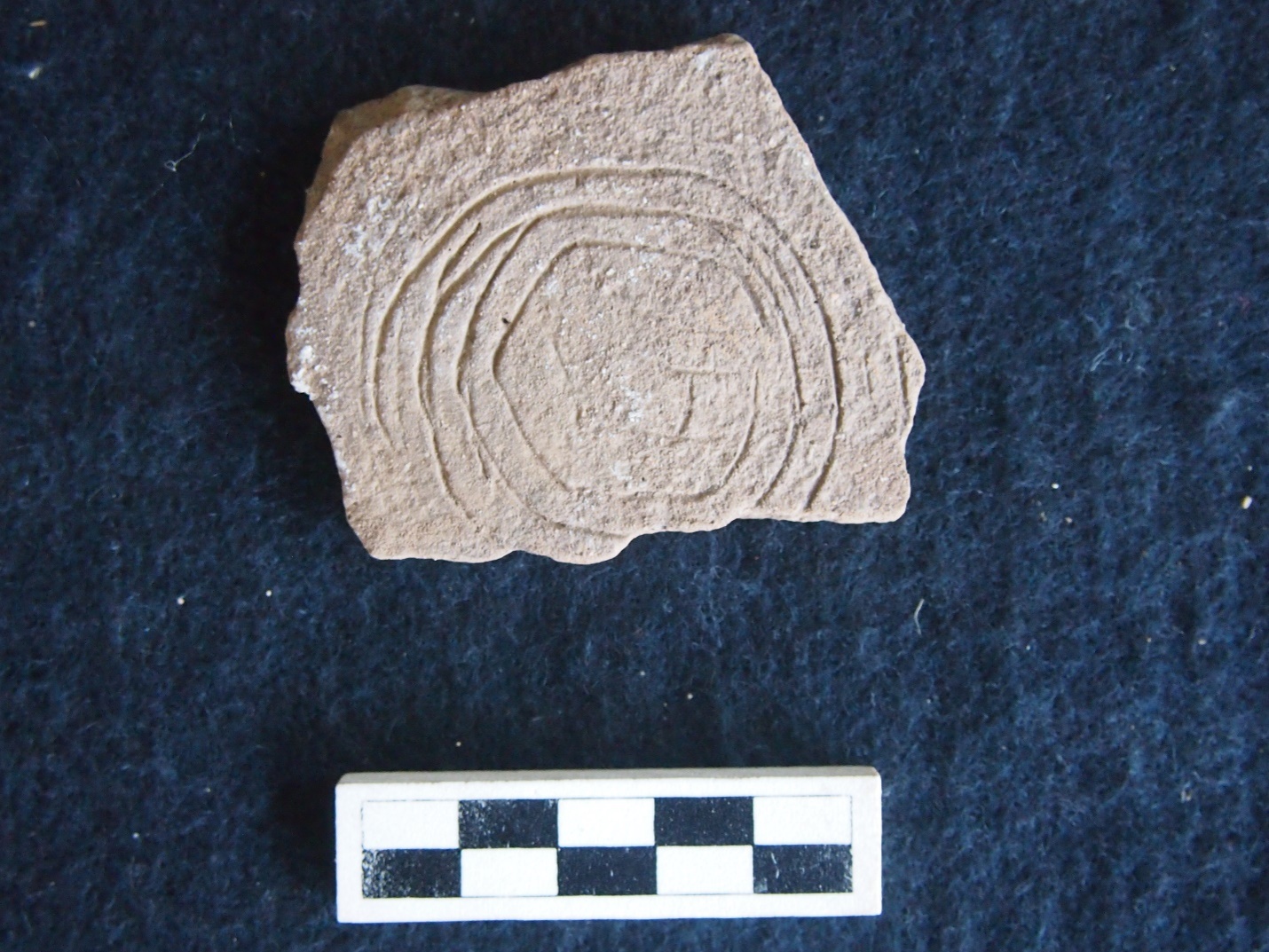


Appendix A.61: BS0065


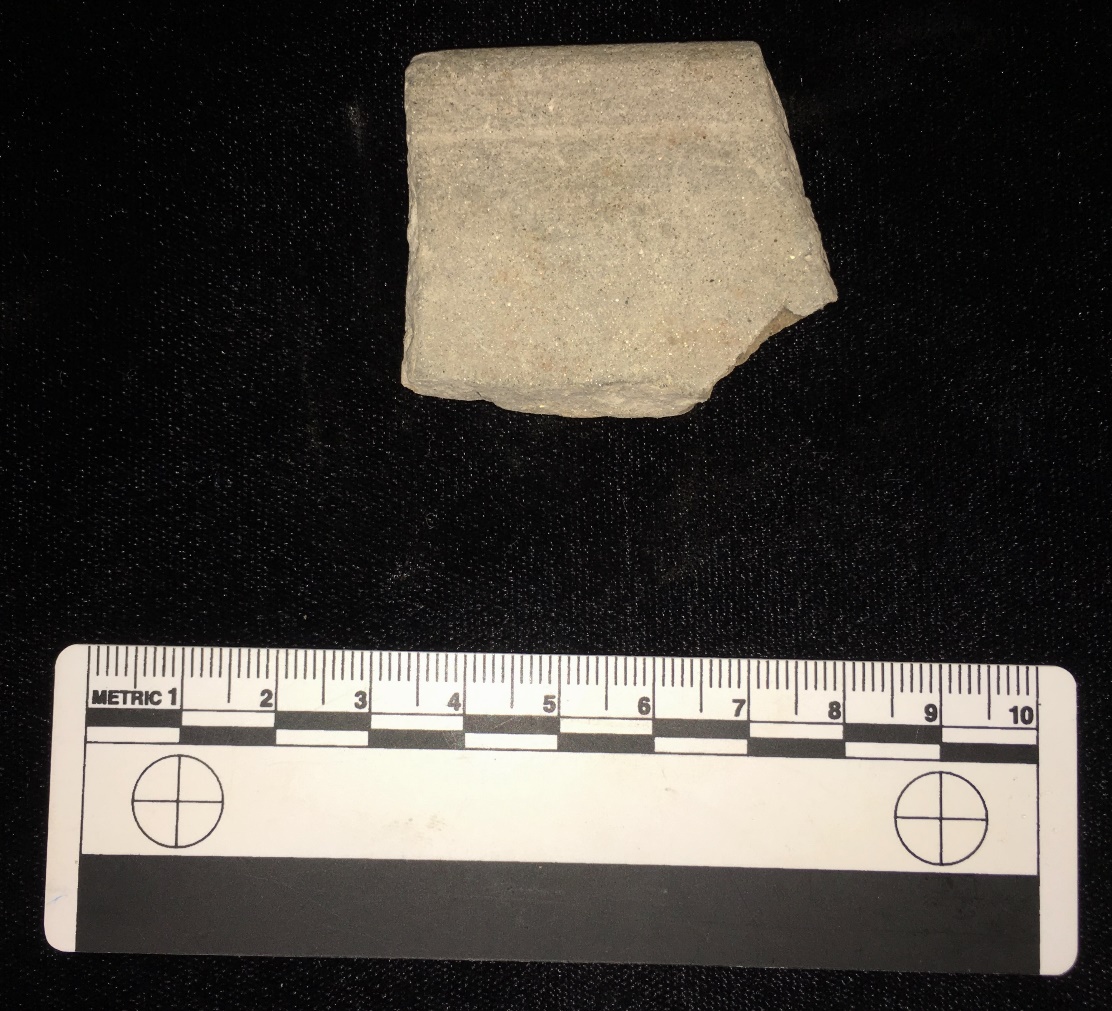


Appendix A.62: BS0066


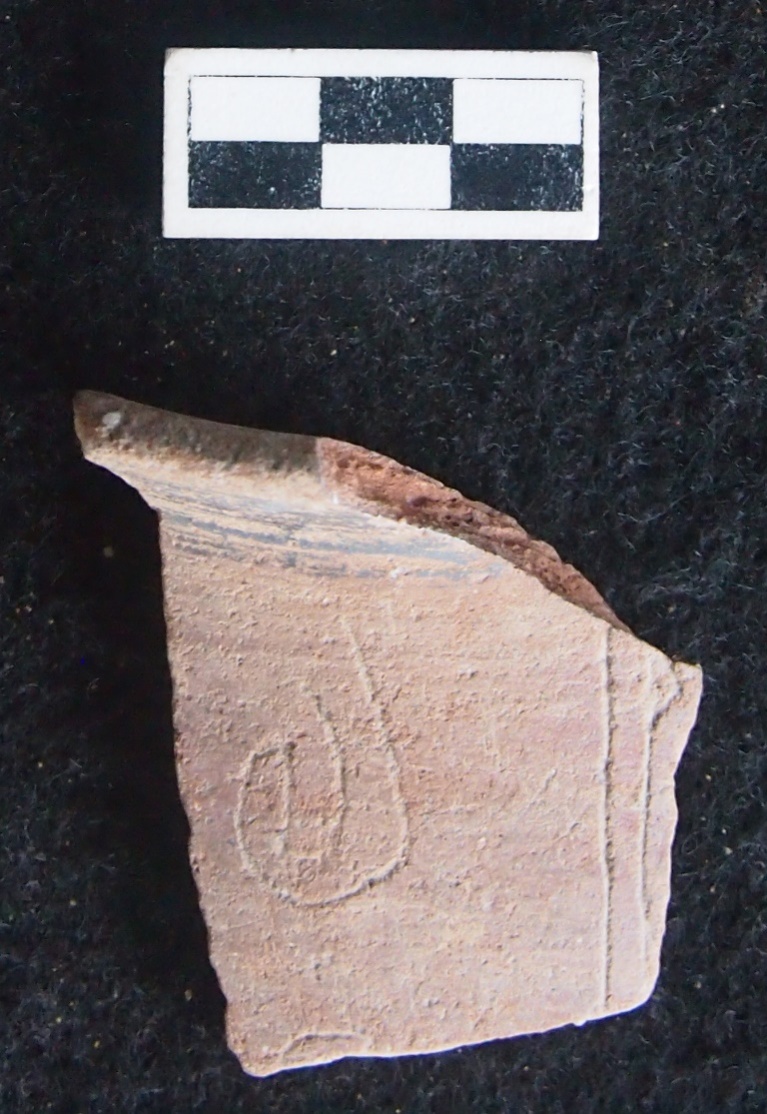


Appendix A.63: BS0067

**Appendix B**

**Mahalanobiscalculationsconfirming group membership of ceramic sherd samples (based upon first three principal components).**

Membership probabilities(%) for samples from the group: Group 1

Probability for each sample calculated after removal from original group.

ANID Group 1 Group 2 Group 3A Group 3B Group 4 Best Group

BS0029 79.335 0.034 0.004 1.069 0.001 Group 1

BS0030 83.357 0.182 0.009 2.402 0.255 Group 1

BS0043 84.971 1.607 0.027 5.785 8.214 Group 1

BS0059 81.920 0.407 0.011 2.794 0.295 Group 1

BS0067 74.769 0.074 0.005 0.998 0.030 Group 1

--------- -------- -------- -------- -------- -------- ----------

Membership probabilities(%) for samples from the group: Group 2

ANID Group 1 Group 2 Group 3A Group 3B Group 4 Best Group

BS0004 4.542 38.645 0.096 5.407 3.544 Group 2

BS0005 2.556 99.854 0.031 1.035 0.165 Group 2

BS0012 3.539 79.575 0.068 1.702 1.093 Group 2

BS0013 1.533 69.982 0.012 0.430 0.003 Group 2

BS0014 3.851 54.142 0.135 3.368 2.519 Group 2

BS0035 1.865 88.393 0.016 0.592 0.015 Group 2

BS0056 1.416 64.987 0.009 0.266 0.001 Group 2

BS0058 1.892 60.992 0.025 0.594 0.025 Group 2

BS0061 3.292 41.367 0.035 0.973 0.489 Group 2

--------- -------- -------- -------- -------- -------- ----------

Membership probabilities(%) for samples from the group: Group 3A

ANID Group 1 Group 2 Group 3A Group 3B Group 4 Best Group

BS0023 18.071 0.376 92.091 57.104 19.868 Group 3A

BS0024 20.866 0.515 82.600 61.965 27.927 Group 3A

BS0025 22.600 0.564 91.648 63.876 29.018 Group 3A

BS0028 25.589 0.959 60.717 24.632 34.930 Group 3A

BS0031 18.000 0.327 66.139 47.104 14.536 Group 3A

BS0063 27.363 0.874 63.137 45.182 32.365 Group 3A

--------- -------- -------- -------- -------- -------- ----------

Membership probabilities(%) for samples from the group: Group 3B

ANID Group 1 Group 2 Group 3A Group 3B Group 4 Best Group

BS0003 7.794 0.014 0.066 76.228 0.097 Group 3B

BS0008 11.906 0.106 1.436 55.411 9.564 Group 3B

BS0020 18.910 0.155 0.550 90.887 4.564 Group 3B

BS0026 17.501 0.149 0.085 57.068 2.304 Group 3B

BS0032 15.297 0.176 5.064 78.239 8.881 Group 3B

BS0040 23.776 2.272 4.143 66.105 49.276 Group 3B

BS0054 10.044 0.022 0.084 74.865 0.124 Group 3B

--------- -------- -------- -------- -------- -------- ----------

Membership probabilities(%) for samples from the group: Group 4

ANID Group 1 Group 2 Group 3A Group 3B Group 4 Best Group

BS0001 23.572 5.219 0.134 16.546 58.717 Group 4

BS0002 6.949 0.037 0.016 0.620 38.358 Group 4

BS0006 9.315 5.591 0.123 9.449 14.437 Group 4

BS0007 7.770 0.632 0.120 7.878 52.781 Group 4

BS0010 5.504 0.040 0.051 0.564 42.321 Group 4

BS0011 15.081 9.126 3.176 38.464 73.643 Group 4

BS0015 53.989 4.836 0.123 12.904 67.379 Group 4

BS0017 13.956 0.349 0.077 1.914 82.777 Group 4

BS0022 29.326 10.389 1.516 38.566 87.822 Group 4

BS0033 6.452 0.723 2.043 9.699 11.292 Group 4

BS0034 10.895 30.312 0.593 12.417 36.476 Group 4

BS0036 10.332 29.609 0.498 10.091 32.597 Group 4

BS0037 6.274 0.040 0.023 0.642 53.398 Group 4

BS0038 6.569 0.290 0.546 3.527 56.290 Group 4

BS0039 6.888 0.062 0.031 0.898 64.720 Group 4

BS0041 24.296 0.825 2.171 12.693 31.436 Group 4

BS0042 5.510 0.099 0.202 1.493 47.778 Group 4

BS0044 20.765 0.898 2.079 35.365 35.104 Group 3B

BS0047 43.281 5.266 0.446 23.162 89.595 Group 4

BS0048 12.289 12.202 0.278 11.473 24.611 Group 4

BS0049 31.940 7.632 0.178 19.615 70.822 Group 4

BS0051 27.782 9.837 1.848 41.312 91.736 Group 4

BS0052 6.041 0.039 0.024 0.718 51.759 Group 4

BS0053 4.387 0.013 0.014 0.265 26.880 Group 4

BS0055 33.459 2.596 0.206 9.025 91.064 Group 4

BS0057 9.925 0.173 0.050 1.567 83.760 Group 4

BS0060 23.992 0.914 50.507 20.227 33.493 Group 3A

BS0062 54.921 4.106 0.067 11.902 31.812 Group 1

BS0064 33.291 2.846 6.242 39.160 75.836 Group 4

BS0066 21.875 1.429 0.330 15.759 31.637 Group 4

BS0065 14.051 0.496 1.439 25.717 20.664 Group 3B

--------- -------- -------- -------- -------- -------- ----------

Membership probabilities(%) for samples from the group: Unassigned

ANID Group 1 Group 2 Group 3A Group 3B Group 4 Best Group

BS0009 7.154 0.071 18.550 35.040 2.770 Group 3B

BS0016 1.402 0.491 0.037 0.430 0.002 Group 1

BS0018 4.773 0.025 1.182 0.535 0.261 Group 1

BS0019 6.324 0.500 0.054 5.578 1.220 Group 1

BS0021 2.868 0.002 0.026 5.555 0.011 Group 3B

BS0027 6.492 0.012 0.099 23.746 0.017 Group 3B

BS0045 11.182 0.185 3.916 14.321 4.347 Group 3B

BS0046 5.689 1.017 0.092 6.351 2.596 Group 3B

BS0050 4.175 0.011 0.010 0.104 1.818 Group 1

--------- -------- -------- -------- -------- -------- ----------
